# Supplementary material for: Pan-RAF inhibitor exarafenib targets BRAF class II/III NSCLC and reveals ARAF-KSR1 resistance and combination strategies
Source: Nat Commun. 2026 Feb 7;17:2484. doi: 10.1038/s41467-026-69216-3 (PMC12992618; doi:10.1038/s41467-026-69216-3)

# Supplementary Information

# Supplementary Figures


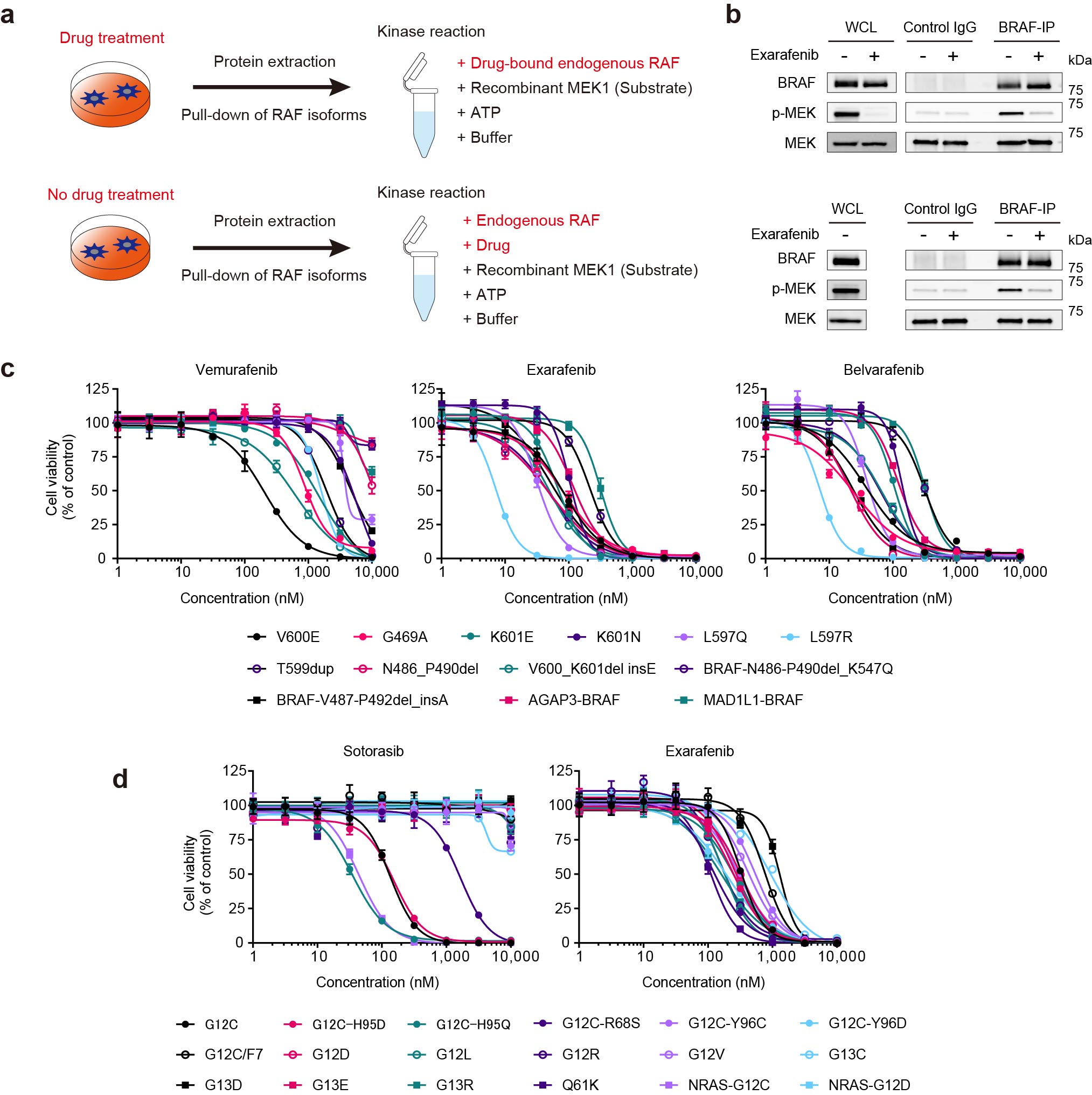


## Supplementary Figure 1. Exarafenib inhibits endogenous RAF activity and shows broad efficacy across RAF- and RAS-mutant Ba/F3 models.

**a,** Schematic representation of the experimental design for assessing the effects of RAF inhibitors on endogenous RAF kinase activity. Two approaches were used: drug administration to cells followed by protein extraction and IP-kinase assay (top), and drug treatment only at the ex vivo kinase reaction stage after BRAF-IP (bottom). **b,** Western blot analysis of BRAF kinase activity in the NCI-H2405 cell line treated with exarafenib using the two approaches described in (**a**). Data are representative of three independent experiments with similar results. **c**, Dose-response curves of vemurafenib, exarafenib, and belvarafenib in Ba/F3 models expressing various BRAF mutations. Cell viability was assessed after 72 hours of treatment at different drug concentrations. Data are mean ± s.d. of *n* = 3 biologically independent experiments. A heatmap summarizing the IC50 is shown in Fig. 2d. **d**, Cell viability curves for Fig. 2e. The models include multiple KRAS mutations and some NRAS mutations. Cell viability was measured following 72-hour exposure to varying concentrations of each inhibitor and expressed as a percentage relative to untreated controls. Data are mean ± s.d. of *n* = 3 biologically independent experiments. Source data are provided as a Source Data file.


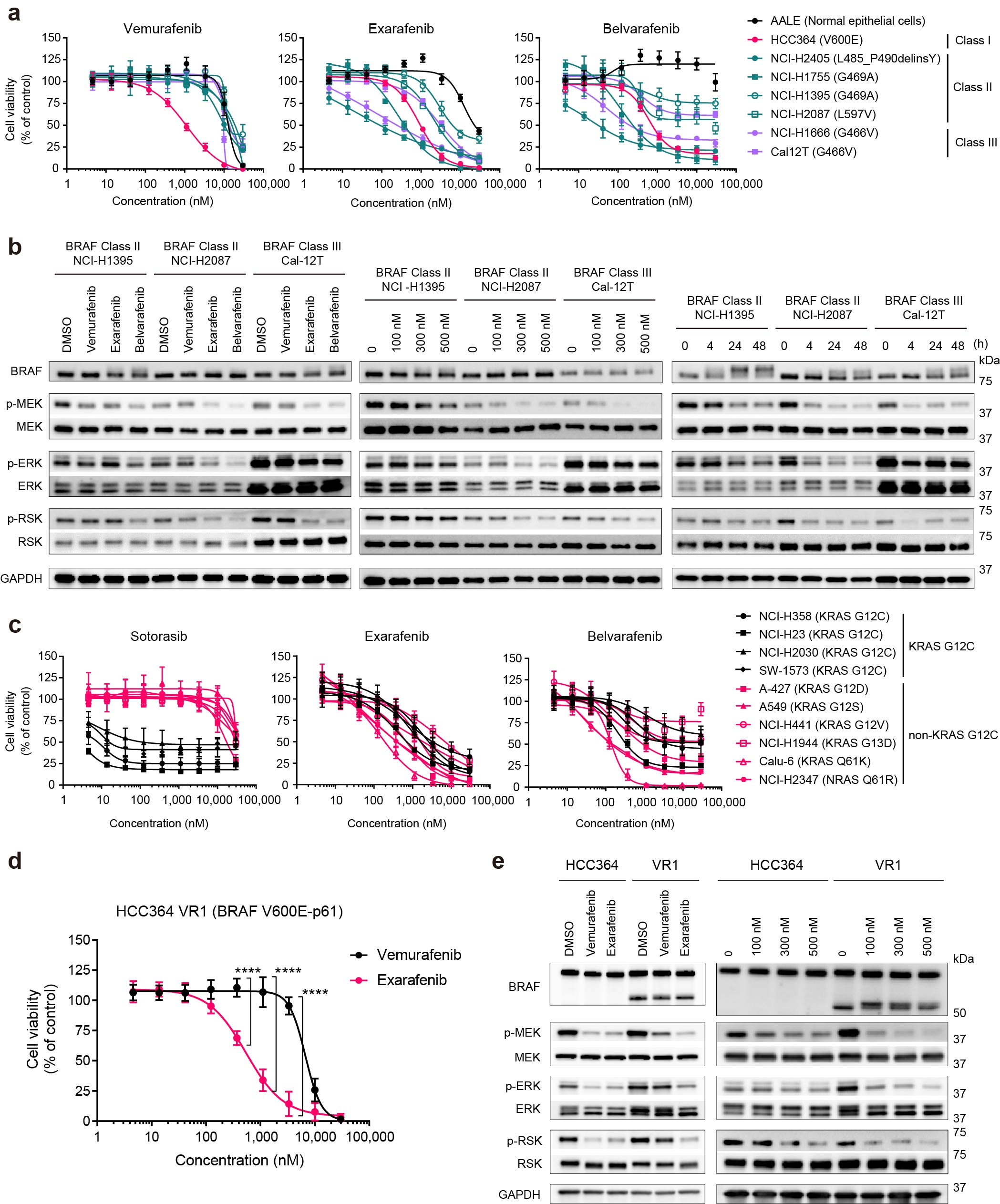


## Supplementary Figure 2. Exarafenib demonstrates broad-spectrum efficacy against diverse BRAF and KRAS mutations in lung cancer cell lines and overcomes vemurafenib resistance.

**a,** Dose-response curves showing cell viability of patient-derived lung cancer cell lines harboring BRAF mutations from various functional classes, and the non-cancerous bronchial epithelial cell line AALE, when treated with vemurafenib, exarafenib, or belvarafenib. Data are mean ± s.d. of *n* = 3 biologically independent experiments. Heatmap depicting the IC50 value is shown in Fig. 3a. **b,** Western blot analysis of MAPK signaling components in lung cancer cell lines with low sensitivity to exarafenib. Left: Various RAF inhibitors tested at a single dose (300 nM) for 4 hours for comparison. Middle: Dose-dependent effects of exarafenib at 4 hours treatment. Right: Time-dependent effects of exarafenib at 300 nM. Data are representative of three independent experiments with similar results. **c,** Cell viability assay results comparing the effects of sotorasib, exarafenib, and belvarafenib in lung cancer cell lines harboring various KRAS and NRAS mutations. Data are mean ± s.d. of *n* = 3 biologically independent experiments. **d,** Cell viability assay comparing vemurafenib and exarafenib sensitivity in parental HCC364 and HCC364-VR1 cells. Exarafenib demonstrates higher potency against resistant cells compared to vemurafenib. Data are mean ± s.d. of *n* = 3 biologically independent experiments. *P*-values at selected points of interest (two-sided Student’s *t*-test): 1.3 × 10^-9^***, 6.2 × 10^-10^***, 1.0 × 10^-11^*** (from left to right). **P <* 0.05, ***P <* 0.01, ****P <* 0.001, *****P <* 0.0001. **e,** Western blot analysis of p-MEK, p-ERK and p-RSK in HCC364 and HCC364-VR1 cells treated with vemurafenib or exarafenib. Exarafenib robustly inhibits MAPK signaling in resistant cells. Data are representative of three independent experiments with similar results. Source data are provided as a Source Data file.


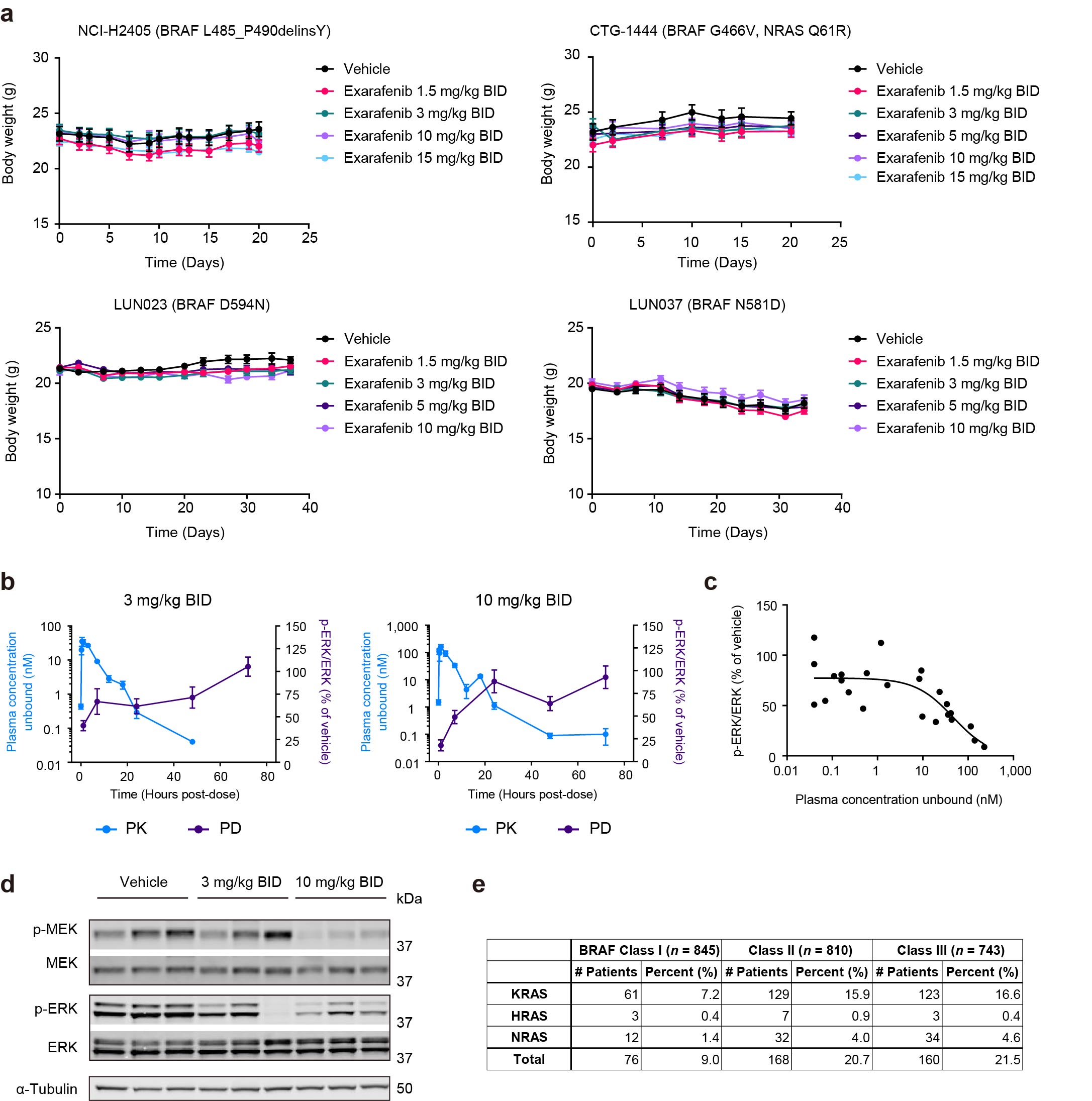


## Supplementary Figure 3. Exarafenib demonstrates efficacy in lung cancer models and exhibits dose- and time-dependent pharmacokinetics and pharmacodynamics.

**a,** In vivo tolerability of exarafenib in BRAF-mutant NSCLC xenograft models NCI-H2405 (BRAF L485_P490delinsY), CTG-1444 (BRAF G466V), LUN023 (BRAF D594N), and LUN037 (BRAF N581D). Mice were treated with exarafenib at various dosages twice daily (BID). Body weights were measured over time. Data represent mean ± s.e.m. (*n* = 9 mice per group). **b,** Pharmacokinetic (PK) and pharmacodynamic (PD) profiles of exarafenib in mice bearing NCI-H2405 xenografts treated with 3 or 10 mg/kg BID for 72 hours. Plasma concentrations confirm linear PK (left y-axis), and p-ERK inhibition in tumor lysates is observed for approximately 24 hours following the final dose (right y-axis). **c,** In vivo EC_50_ determination (14.67 nM) based on the relationship between p-ERK inhibition and plasma concentration of unbound exarafenib in NCI-H2405 xenografts. **d,** Western blot analysis of NCI-H2405 tumor samples treated with vehicle, 3 mg/kg BID, or 10 mg/kg BID doses of exarafenib. Three independent samples from each group were analyzed. The results show dose-dependent inhibition of p-MEK and p-ERK levels. Total MEK (MEK), total ERK (ERK), and α-Tubulin were used as loading controls. **e,** Frequency of concurrent RAS (KRAS, HRAS, and NRAS) mutations in patients with BRAF Class I, II, and III NSCLC. Data indicate the number of patients and percentage within each class. Specific mutations and their frequencies are provided in Supplementary Data 2. Source data are provided as a Source Data file.


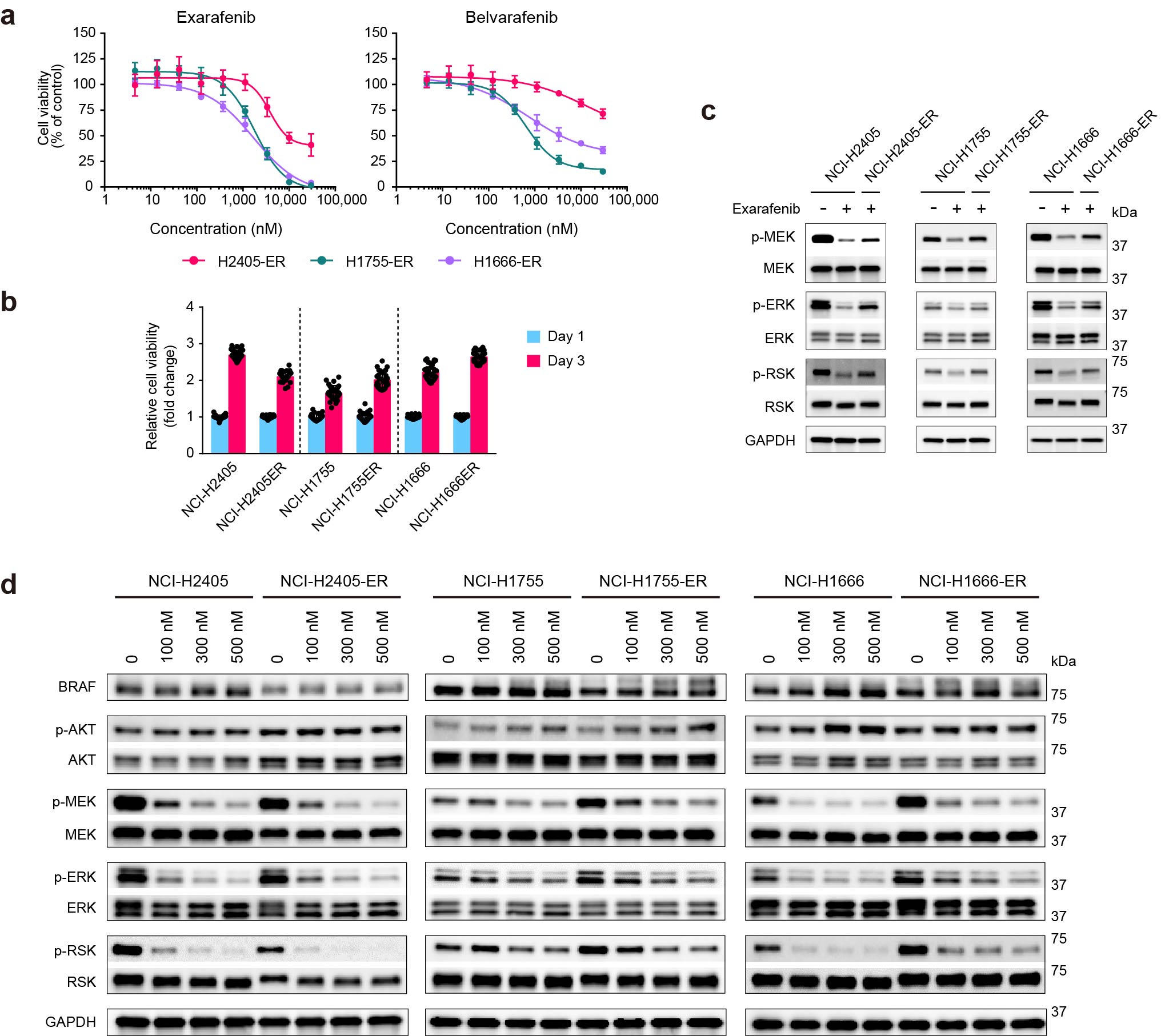


## Supplementary Figure 4. Characterization of exarafenib-resistant lung cancer cell lines and their status on MAPK and AKT signaling pathways.

**a,** Cell viability curves for exarafenib (left) and belvarafenib (right) in exarafenib-resistant cell lines (NCI-H2405-ER, NCI-H1755-ER, and NCI-H1666-ER). Data are mean ± s.d. of *n* = 3 biologically independent experiments. **b,** Relative cell viability (fold change) of parental cells and their corresponding exarafenib-resistant derivatives. Resistant cells were cultured in the presence of 1 μM exarafenib, while parental cells were grown without drug exposure. Measurements were taken on day 1 (blue bars) and day 3 (red bars). Data are presented as fold change relative to day 1. Data are mean ± s.d. of *n* = 3 biologically independent experiments with individual data points overlaid. The results demonstrate that exarafenib-resistant cell lines maintain proliferation capabilities comparable to their parental counterparts, even under continuous exarafenib exposure. **c,** Analysis of parental and exarafenib-resistant NCI-H2405, NCI-H1755, and NCI-H1666 cell lines by western blot. Parental cells were treated with either DMSO or 1 μM exarafenib for 4 hours, while resistant cell lines were cultured in 1 μM exarafenib. The levels of p-MEK, p-ERK, and p-RSK were assessed. The resistant cell lines showed sustained levels of p-MEK, p-ERK, and p-RSK compared to their parental counterparts. Data are representative of three independent experiments with similar results. **d,** Western blot analysis of MAPK and AKT signaling in parental and exarafenib-resistant NCI-H2405, NCI-H1755, and NCI-H1666 cell lines treated with various doses of exarafenib for 4 hours. Data are representative of three independent experiments with similar results. Source data are provided as a Source Data file.


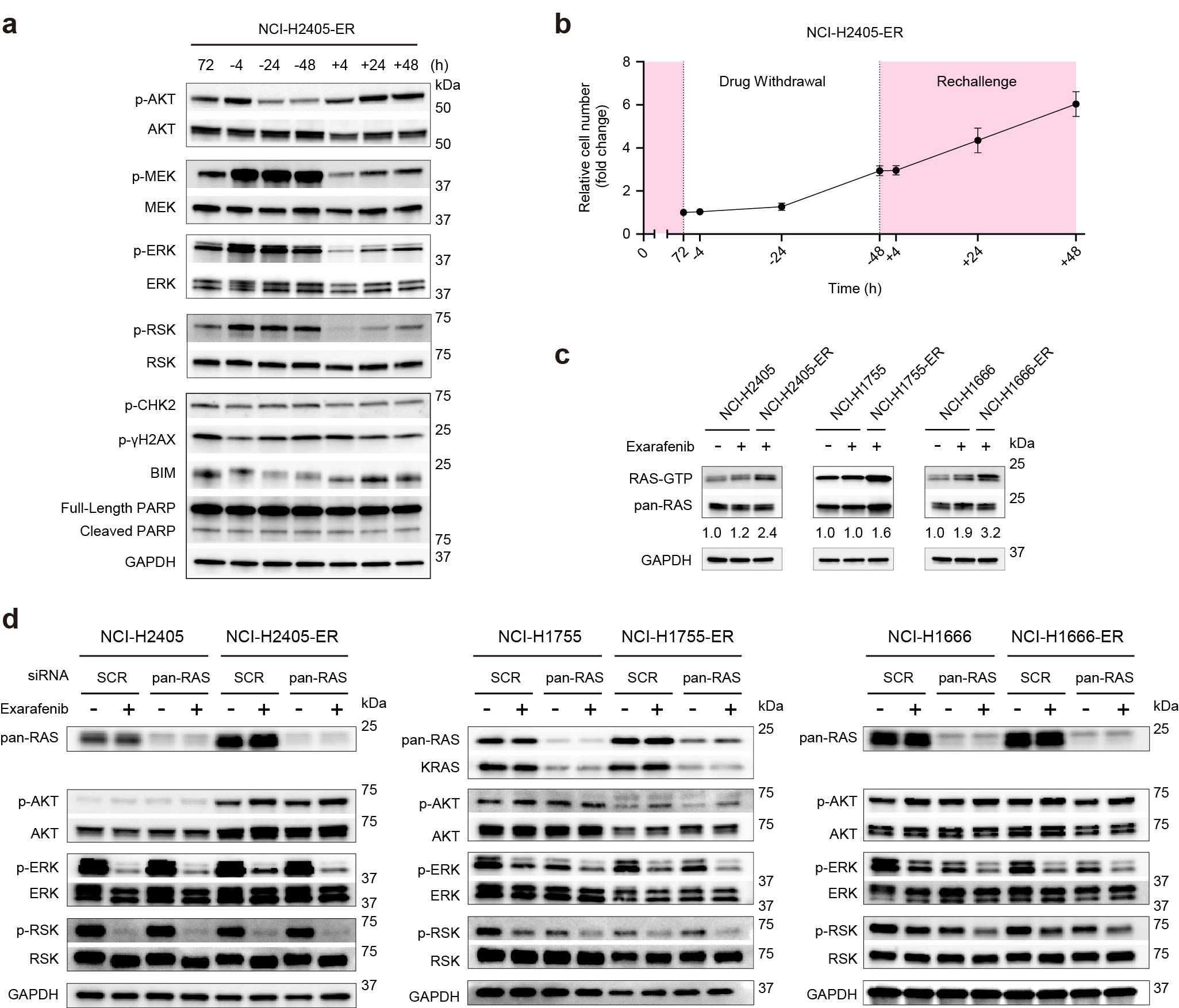


## Supplementary Figure 5. RAS-mediated pathway reactivation underlies sustained proliferation in exarafenib-resistant cells

**a,** Time-course analysis of MAPK pathway components, DNA damage markers, and apoptosis markers in NCI-H2405-ER cells during drug withdrawal and re-challenge. Cells were initially maintained under exarafenib treatment for 72 hours, then switched to drug-free medium (negative time points) followed by exarafenib re-treatment (positive time points). Data are representative of three independent experiments with similar results. **b,** Cell number fold change over time for exarafenib-resistant NCI-H2405-ER cells. Cells were initially grown in the presence of exarafenib, then subjected to drug withdrawal (white area), followed by drug re-challenge (red shaded area). Data points represent relative cell number normalized to the 72-hour time point (set as 1-fold). Data are shown as mean ± s.d. of *n* = 3 biologically independent experiments. **c,** RAS-RBD assay assessing RAS activation (RAS-GTP) in parental and resistant cells. Parental cells were treated with either DMSO or 1 μM exarafenib for 4 hours, while resistant cell lines were cultured in 1 μM exarafenib. Data are representative of three independent experiments with similar results. **d,** Western blot analysis of RAS knockdown effects on MAPK and AKT signaling in parental and resistant lines. Cells were treated with either DMSO or 500 nM exarafenib for 4 hours. Data are representative of three independent experiments with similar results. Source data are provided as a Source Data file.


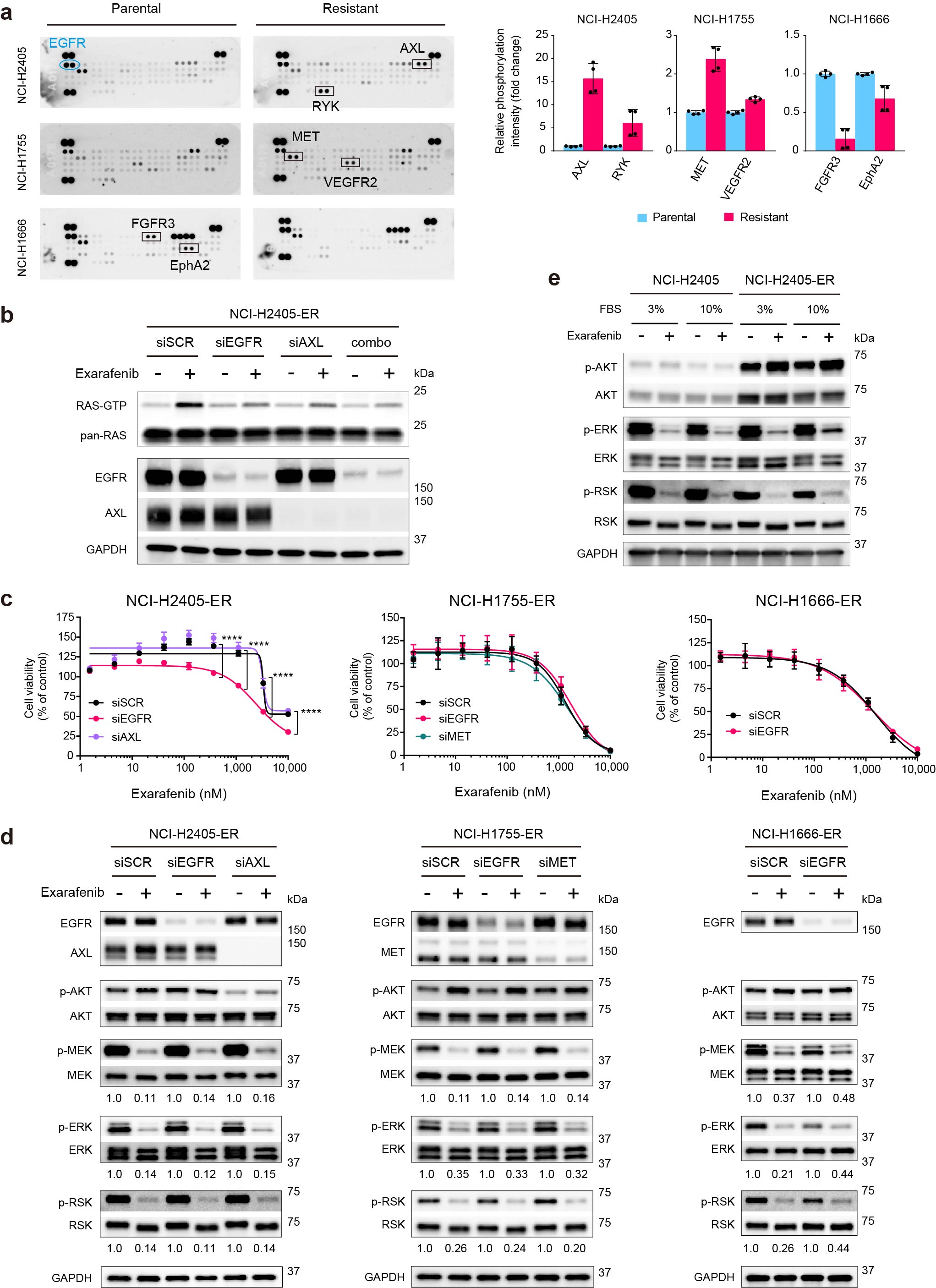


## Supplementary Figure 6. RTK profiles and effects of RTK knockdown in exarafenib-resistant lung cancer cell lines.

**a,** Phospho-RTK array comparing parental and resistant cell lines (left). Black boxes highlight the most significantly changed RTKs in each resistant cell line. The intensity of phosphorylation was quantified and shown as bar graphs (right). Each intensity was normalized using positive controls. Data are representative of two independent experiments with similar results. **b,** Western blot analysis of RAS activation in NCI-H2405-ER resistant cells following individual and combined RTK knockdown. Cells were transfected with scrambled control (siSCR), siEGFR, siAXL, or combination of both and treated with exarafenib (+) or vehicle (-). Data are representative of three independent experiments with similar results. **c,** Cell viability curves for exarafenib in NCI-H2405-ER cells transfected with siRNA targeting SCR, EGFR, or AXL (left), NCI-H1755-ER cells transfected with siRNA targeting SCR, EGFR, or MET (middle), and NCI-H1666-ER cells transfected with siRNA targeting SCR or EGFR (right). Data are mean ± s.d. of *n* = 3 biologically independent experiments. *P*-values at selected points of interest (two-sided Student’s *t*-test): 9.72 × 10^-13^****, 1.23 × 10^-16^****, 8.25 × 10^-16^****, 1.29 × 10^-18^**** (from left to right). **P <* 0.05, ***P <* 0.01, ****P <* 0.001, *****P <* 0.0001. **d,** Western blot analysis of EGFR, AXL, MET, p-AKT, AKT, p-MEK, MEK, p-ERK, ERK, p-RSK, RSK, and GAPDH in exarafenib-resistant cell lines transfected with indicated siRNA and treated with 500 nM exarafenib for 4 hours. Data are representative of three independent experiments with similar results. **e,** MAPK pathway analysis in NCI-H2405 parental and resistant cells treated with exarafenib (500 nM) for 4 hours under low (3%) or high (10%) FBS conditions. Data are representative of three independent experiments with similar results. Source data are provided as a Source Data file.


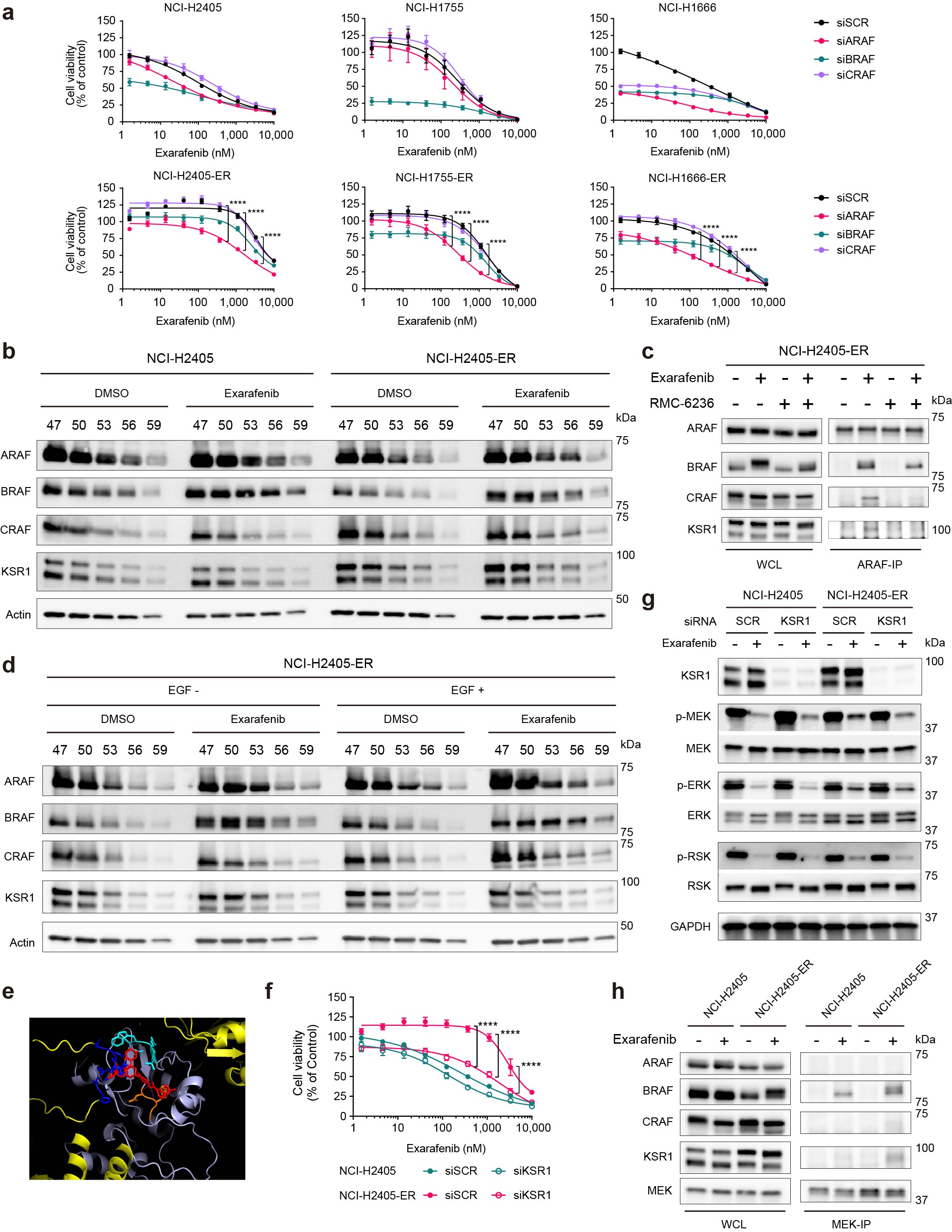


## Supplementary Figure 7. Molecular characterization of ARAF-KSR1 complex-mediated resistance mechanism and functional validation of RAF isoform dependencies

**a,** Dose-response curves for RAF isoform knockdown experiments. Cell viability was measured in parental (top) and exarafenib-resistant (bottom) cell lines following transfection with scrambled control (siSCR, black), siARAF (red), siBRAF (green), or siCRAF (purple) and treatment with increasing concentrations of exarafenib. Data are mean ± s.d. of *n* = 3 biologically independent experiments. *P*-values at selected points of interest (two-sided Student’s *t*-test). For NCI-H2405-ER, *P =* 1.04 × 10^-13^****, 1.66 × 10^-14^****, 5.5 × 10^-15^**** (from left to right). For NCI-H1755-ER, *P =* 1.21 × 10^-14^****, 2.4 × 10^-12^****, 8.39 × 10^-11^****. For NCI-H1666-ER, *P =* 1.25 × 10^-16^****, 5.24 × 10^-12^****, 8.74 × 10^-13^****. *****P <* 0.0001. Heatmap depicting the IC50 value is shown in Fig. 5a. **b,** CETSA analysis of RAF isoforms and KSR1 in parental and resistant cells treated with DMSO or exarafenib (500 nM) for 24 hours. Exarafenib enhanced thermal stabilization of ARAF and CRAF in resistant cells, while BRAF stabilization was largely maintained. KSR1 showed minimal stabilization. Data are representative of three independent experiments with similar results. **c,** Co-immunoprecipitation analysis investigating RAS-dependent regulation of ARAF-KSR1 complex formation. NCI-H2405-ER cells were treated with DMSO, exarafenib (500 nM), RMC-6236 (1 μM), or combination of both for 24 hours. ARAF immunoprecipitation demonstrates that the multi-RAS inhibitor RMC-6236 suppresses ARAF-KSR1 interaction, confirming RAS-dependent regulation of this protein complex. Data are representative of three independent experiments with similar results. **d,** CETSA analysis of exarafenib-resistant NCI-H2405-ER cells treated with DMSO or exarafenib (500 nM) for 24 hours, with or without EGF stimulation. EGF enhanced exarafenib-induced thermal stabilization of all RAF isoforms. KSR1 showed minimal stabilization regardless of EGF. Data are representative of three independent experiments with similar results. **e,** Docking analysis of exarafenib in the AlphaFold-predicted ARAF–KSR1 complex. Exarafenib binds the ATP pocket of ARAF, contacting the hinge region and DFG motif, with additional contacts to a short KSR1 segment. ARAF chain: gray, KSR1 chain: yellow, Exarafenib: red, ARAF gatekeeper and hinge region (Thr382–Cys385): Cyan, ARAF DFG motif (Asp447–Phe448–Gly449): orange, KSR1 segment (Pro408–Asn413): blue, Chemical bonds: magenta. **f,** Cell viability dose-response curves showing the effect of KSR1 knockdown on exarafenib sensitivity in NCI-H2405 parental and resistant cells. Cells were transfected with scrambled control (SCR) or KSR1 siRNA (siKSR1) and treated with increasing concentrations of exarafenib. Data are mean ± s.d. of *n* = 3 biologically independent experiments. *P*-values at selected points of interest (two-sided Student’s *t*-test). *P =* 2.43 × 10^-14^****, 3.9 × 10^-13^****, 5.67 × 10^-6^**** (from left to right). *****P <* 0.0001. Heatmap depicting the IC50 value is shown in Fig. 5h. **g,** Western blot analysis of MAPK pathway components in NCI-H2405 and NCI-H2405-ER cells following KSR1 knockdown and exarafenib treatment. Cells were transfected with scrambled control (SCR) or KSR1 siRNA and treated with exarafenib (+) or vehicle (-). Data are representative of three independent experiments with similar results. **h,** Co-immunoprecipitation analysis of MEK complexes in parental (NCI-H2405) and resistant (NCI-H2405-ER) cells treated with DMSO or exarafenib (500 nM) for 24 hours. Data are representative of three independent experiments with similar results. Source data are provided as a Source Data file.


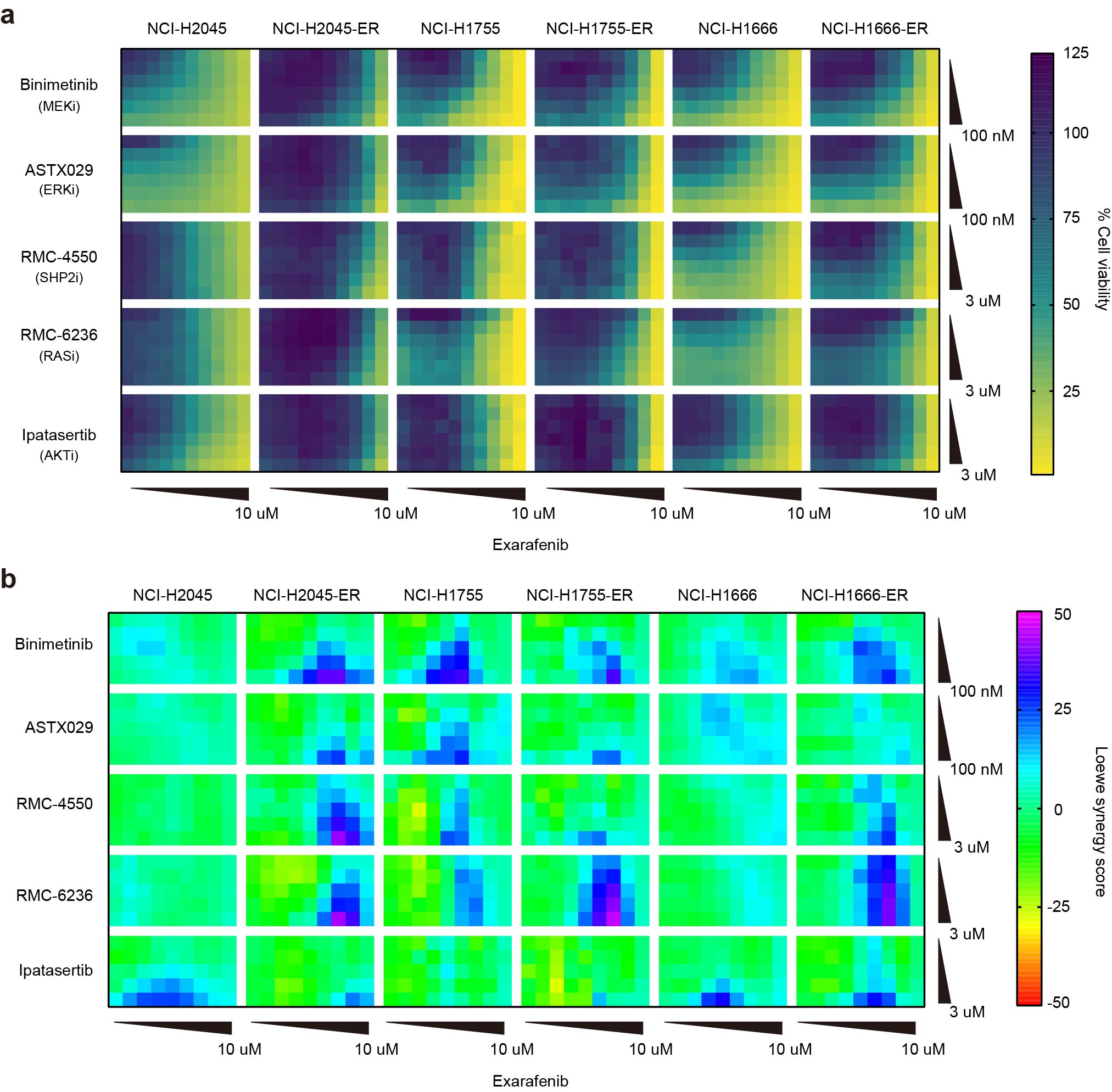


## Supplementary Figure 8. Effects of exarafenib combined with binimetinib, ASTX029, RMC-4550, RMC-6236 and ipatasertib in parental and exarafenib-resistant lung cancer cell lines.

**a,** Heatmap of cell viability for exarafenib combined with binimetinib, ASTX029, RMC-4550, RMC-6236 or ipatasertib in parental and resistant NCI-H2405, NCI-H1755, and NCI-H1666 cells. Lighter shades represent more potent inhibition. Data represent the mean of *n* = 3 biologically independent experiments. **b,** Heatmap of synergy scores for exarafenib combined with binimetinib, ASTX029, RMC-4550, or ipatasertib in parental and exarafenib-resistant NCI-H2405, NCI-H1755, and NCI-H1666 cell lines. Synergy scores were calculated using the Loewe model on dataset from (**a**). Positive scores indicate synergy. Source data are provided as a Source Data file.

**
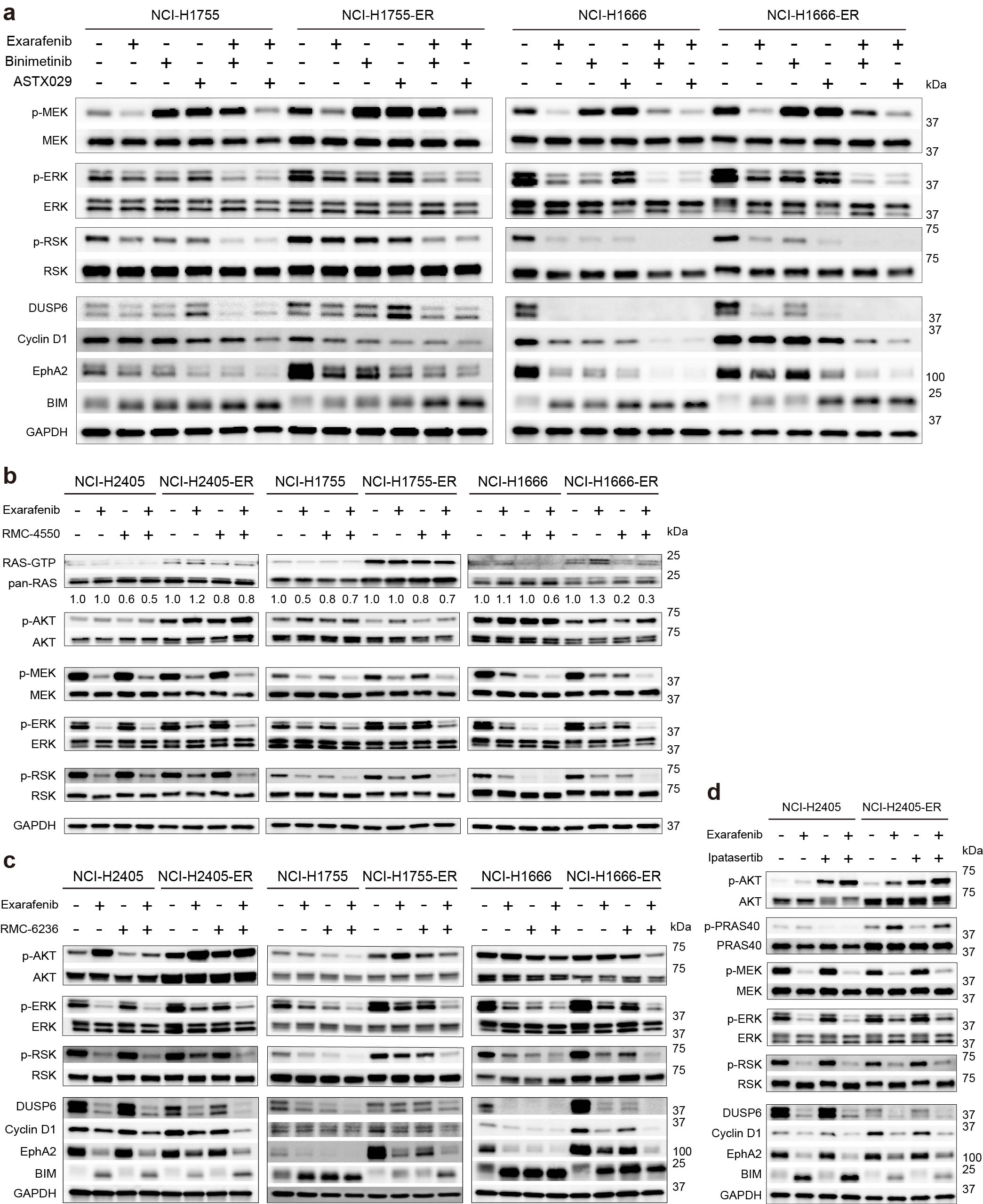
**

## Supplementary Figure 9. Mechanistic insights and combinatorial efficacy of exarafenib and MAPK pathway inhibitors in lung cancer cell lines.

**a,** Immunoblot analysis of MEK, ERK, and RSK phosphorylation, along with DUSP6, Cyclin D1, EphA2, BIM, and GAPDH expression in parental and exarafenib-resistant NCI-H1755 and NCI-H1666 cell lines treated with exarafenib (500 nM), binimetinib (50 nM), ASTX029 (50 nM), or a combination of exarafenib with either binimetinib or ASTX029 for 48 hours. Data are representative of two independent experiments with similar results. **b,** Analysis of RAS-GTP levels, total RAS, AKT activation, and key MAPK signaling components in parental and exarafenib-resistant NCI-H2405, NCI-H1755, and NCI-H1666 cell lines treated with exarafenib (500 nM), RMC-4550 (1 μM), or a combination of both for 4 hours. Drug concentrations were selected based on their high synergy scores in resistant cells, as calculated from the comprehensive drug screening (Supplementary Fig. 8b). Data are representative of two independent experiments with similar results. **c,** Immunoblot analysis of p-AKT, AKT, p-MEK, MEK, p-ERK, ERK, p-RSK, RSK, DUSP6, Cyclin D1, EphA2, BIM, and GAPDH levels in parental and exarafenib-resistant NCI-H2405, NCI-H1755 and NCI-H1666 cell lines treated with exarafenib (500 nM), RMC-6236 (1 μM), or a combination of both for 48 hours. Data are representative of two independent experiments with similar results. **d,** Examination of AKT and PRAS40 phosphorylation alongside MAPK signaling regulators, including DUSP6, Cyclin D1, EphA2, and BIM, in parental and exarafenib-resistant NCI-H2405 cells treated with exarafenib (500 nM), ipatasertib (1 μM), or a combination of both for 48 hours. The phosphorylation state of the PRAS40 protein, a downstream target of AKT, was included to verify the activity of ipatasertib. Data are representative of two independent experiments with similar results. Source data are provided as a Source Data file.


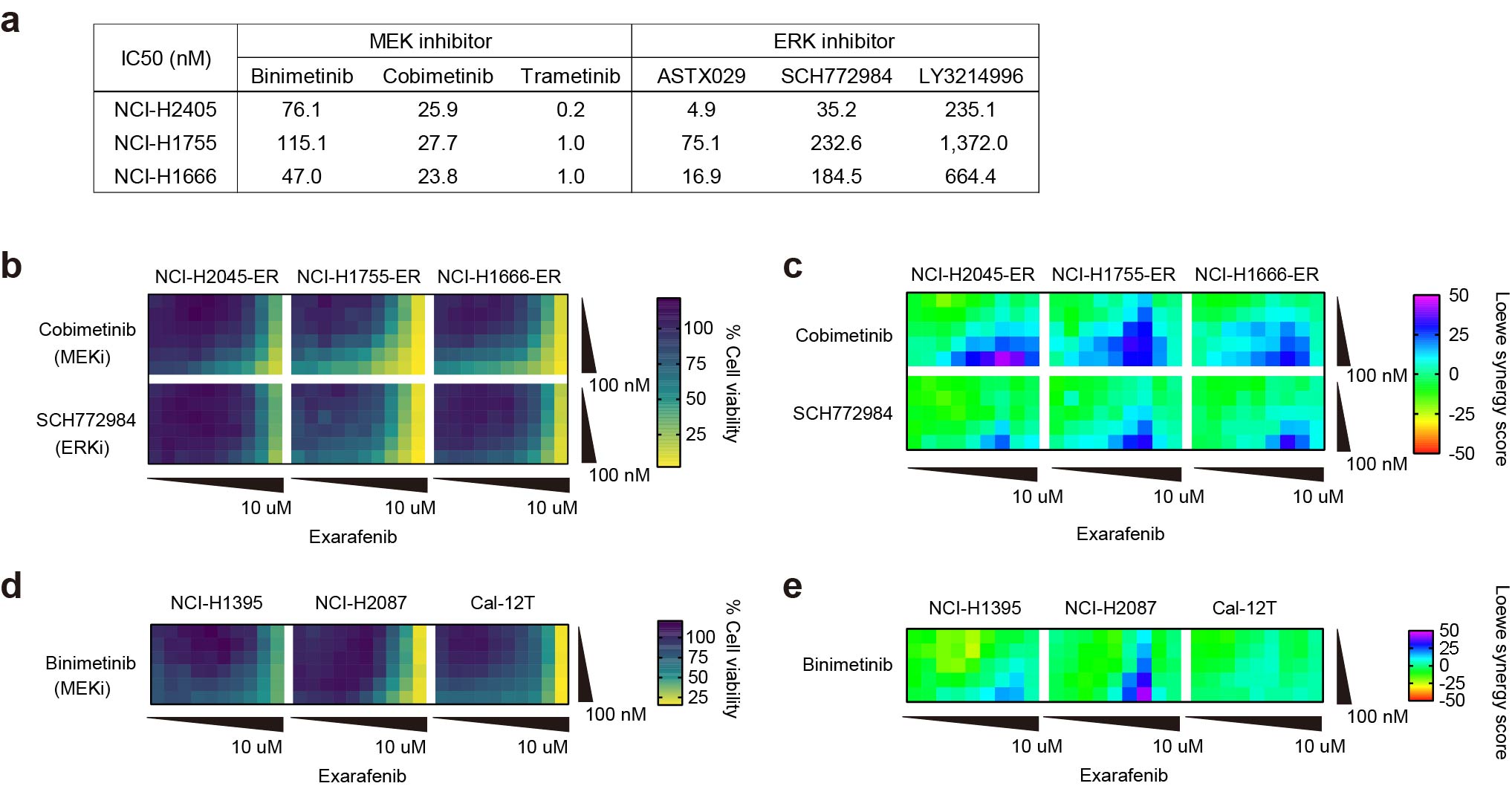


## Supplementary Figure 10. Extended analysis of exarafenib's efficacy in combination with MAPK pathway blockade.

**a,** IC50 values for MEK inhibitors (binimetinib, cobimetinib, trametinib) and ERK inhibitors (ASTX029, SCH772984, LY3214996) in NCI-H2405, NCI-H1755, and NCI-H1666 cell lines calculated by cell viability assay. Data represent the mean of *n* = 3 biologically independent experiments. **b, d**, Cell viability heatmaps for exarafenib combined with cobimetinib or SCH772984 in NCI-H2045-ER, NCI-H1755-ER, and NCI-H1666-ER cells (**b**), and exarafenib combined with binimetinib in NCI-H1395, NCI-H2087, and Cal-12T cells (**d**). Heatmaps represent the percentage of viable cells relative to untreated control. Data represent the mean of *n* = 3 biologically independent experiments. **c, e**, Synergy scores for exarafenib combinations in panels (**b**) and (**d**), respectively, calculated using the Loewe model. Positive scores indicate synergy. Source data are provided as a Source Data file.


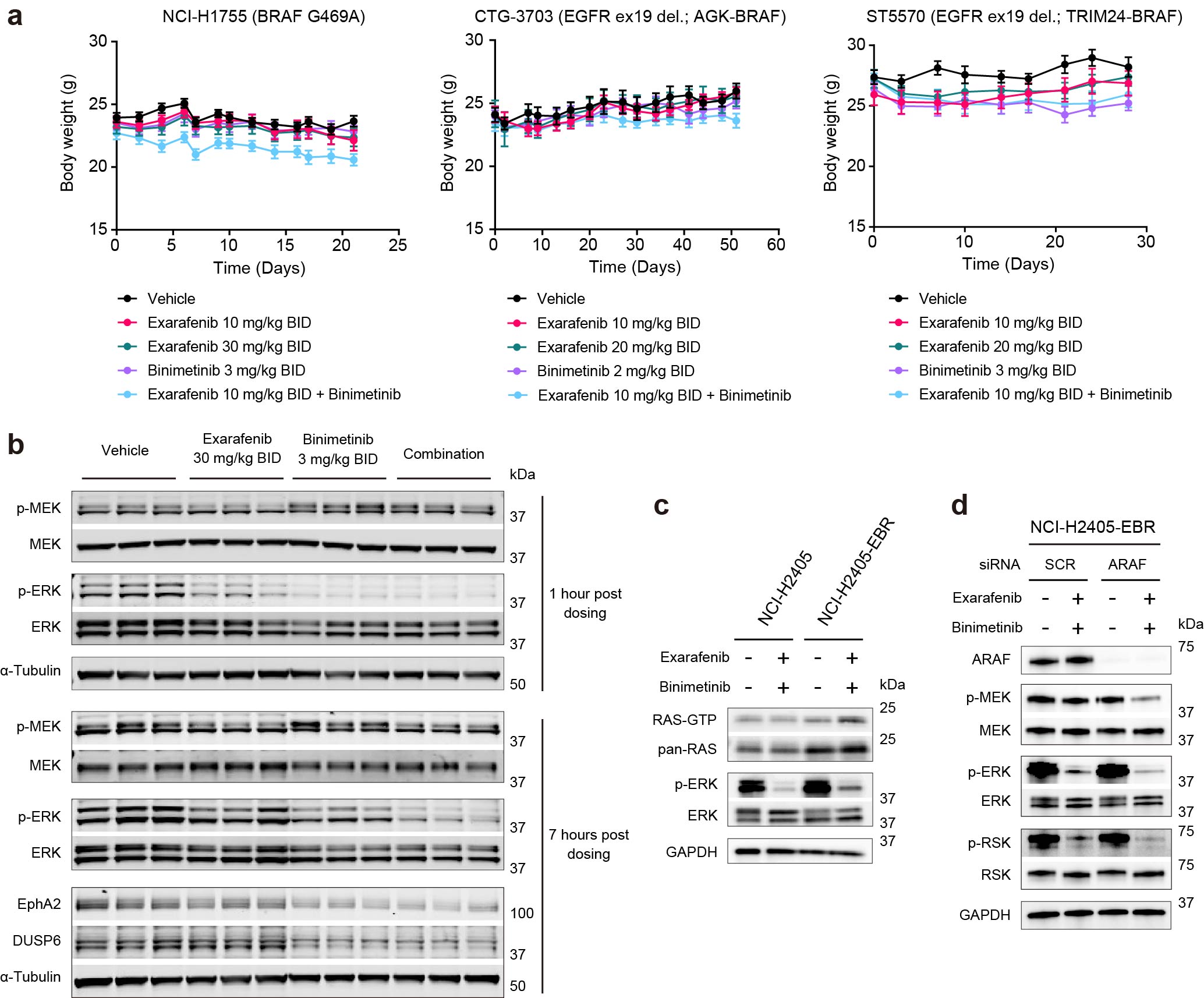


## Supplementary Figure 11. In vivo tolerability and pharmacodynamic effects of exarafenib plus binimetinib combination therapy, and ARAF-mediated resistance mechanisms.

**a,** Body weight curves for xenograft mouse models treated with vehicle, exarafenib (10, 20, or 30 mg/kg, BID), binimetinib (2 or 3 mg/kg, BID), or their combination. Models: NCI-H1755 (BRAF G469A), CTG-3703 (AGK-BRAF), and ST5570 (TRIM24-BRAF). Data: mean ± s.e.m. (*n* = 9 for NCI-H1755; *n* = 6 for CTG-3703 and ST5570). **b,** Western blot analysis of H1755 xenograft tumor samples at 1-hour and 7-hour post-dosing with vehicle, exarafenib (30 mg/kg BID), binimetinib (3 mg/kg BID), and their combination. Three independent samples from each group were analyzed. Levels of p-MEK, MEK, p-ERK, ERK, EphA2, DUSP6, and α-Tubulin were assessed. **c,** RAS-GTP pulldown assays in NCI-H2405 parental and combination-resistant (EBR) cells. Cells were treated with exarafenib (500 nM) in combination with binimetinib (50 nM) for 24 hours. EBR cells exhibit elevated RAS-GTP levels under combination drug treatment compared to parental cells. Data are representative of two independent experiments with similar results. **d,** Western blot analysis of MAPK pathway components in NCI-H2405-EBR cells following ARAF knockdown. Cells were transfected with scrambled control (SCR) or ARAF siRNA and treated with exarafenib (500 nM) and binimetinib (50 nM) combination for 24 hours. Data are representative of two independent experiments with similar results. Source data are provided as a Source Data file.

# Supplementary Tables

## Supplementary Table 1. Classification of BRAF Alterations.

| **BRAF Class** | **BRAF Alterations** |
| --- | --- |
| **Class I** | V600^E/K/R/G/M/V/L/A/D^ |
| **Class II** | Q257^R^, P367^L/S^, E451^Q^, I463^S^, G464^V/E/R/A^, G469^A/R/V/S^, V471^F^, L485^F/W^, N486_A489^delinsK^, N486_P490^del^, T488_P492^del^, K499^E^, L505^H/F^, L525^R^, E586^K^, L597^R/V/Q/S^, T599^I/R^, T599^dup^, V600_K601^delinsE^, K601^E/N/Q/T^ |
| **Class III** | F247^L^, D287^H^, G466^V/E/A/R^, S467^L^, G469^E^, K483^E^, R558^Q^, N581^I/Y/S/T/K^, D594^N/E/G/H/A/Y/V^, F595^L^, G596^R/C/D/V^, T599^A^ |

Classification of BRAF mutations into Class I, II, and III based on their functional and structural characteristics. Class I includes the common V600 mutations. Class II comprises non-V600 activating mutations in the kinase domain. Class III includes RAS-dependent, kinase-dead or kinase-impaired mutations.

## Supplementary Table 2. Number of NSCLC Patients by BRAF Class.

| **BRAF Class** | **No. of NSCLC Patients with BRAF Alteration** | **% of ctDNA Positive NSCLC Patients** | **% of BRAF Positive NSCLC Patients** |
| --- | --- | --- | --- |
| **BRAF Class I Only** | 845 | 1.63 | 34.9 |
| **BRAF Class II Only** | 810 | 1.56 | 33.46 |
| **BRAF Class III Only** | 743 | 1.43 | 30.69 |
| **Multiple Classes** | 23 | 0.04 | 0.95 |
| **Total** | **2,421** | **4.68** | **100** |

Number and percentage of NSCLC patients (*n* = 2,421) harboring BRAF alterations belonging to Class I only (*n* = 845), Class II only (*n* = 810), Class III only (*n* = 743), or multiple classes (*n* = 23).

## Supplementary Table 3. Frequency of Specific BRAF Class I Alterations in NSCLC.

| **Frequency of BRAF Class I mutations, NSCLC** | | |
| --- | --- | --- |
| **Class I BRAF Alterations** | **# Patients** | **Percent (%)** |
| V600E | 827 | 97.87 |
| V600K | 8 | 0.95 |
| V600G | 4 | 0.47 |
| V600M | 3 | 0.36 |
| V600R | 3 | 0.36 |
| V600V | 3 | 0.36 |
| V600A | 1 | 0.12 |
| V600L | 1 | 0.12 |
| Total (distinct) | 845 | 100 |

Frequency and percentage of patients with specific BRAF Class I alterations, with V600E being the most prevalent (97.87%).

## Supplementary Table 4. Frequency of Specific BRAF Class II Alterations in NSCLC.

| **Frequency of BRAF Class II mutations, NSCLC** | | |
| --- | --- | --- |
| **Class II BRAF Alterations** | **# Patients** | **Percent (%)** |
| G469A | 261 | 32.22 |
| G469V | 113 | 13.95 |
| K601E | 106 | 13.09 |
| G469R | 66 | 8.15 |
| G464V | 52 | 6.42 |
| K601N | 33 | 4.07 |
| L485F | 22 | 2.72 |
| L597Q | 21 | 2.59 |
| Q257R | 19 | 2.35 |
| L597R | 17 | 2.1 |
| E586K | 15 | 1.85 |
| L485W | 10 | 1.23 |
| G464E | 8 | 0.99 |
| G469S | 8 | 0.99 |
| L597V | 8 | 0.99 |
| T599dup | 8 | 0.99 |
| L505F | 6 | 0.74 |
| N486_P490del | 6 | 0.74 |
| V471F | 6 | 0.74 |
| G464A | 5 | 0.62 |
| G464R | 4 | 0.49 |
| L525R | 4 | 0.49 |
| T599I | 4 | 0.49 |
| K499E | 3 | 0.37 |
| K601Q | 3 | 0.37 |
| L505H | 3 | 0.37 |
| T599R | 2 | 0.25 |
| V600_K601delinsE | 2 | 0.25 |
| E451Q | 1 | 0.12 |
| I463S | 1 | 0.12 |
| K601T | 1 | 0.12 |
| Total (distinct) | 810 | 100 |

Frequency and percentage of patients with specific BRAF Class II alterations, with G469A (32.22%) and G469V (13.95%) being the most common.

## Supplementary Table 5. Frequency of Specific BRAF Class III Alterations in NSCLC.

| **Frequency of BRAF Class III mutations, NSCLC** | | |
| --- | --- | --- |
| **Class III BRAF Alterations** | **# Patients** | **Percent (%)** |
| D594N | 117 | 15.75 |
| G466V | 111 | 14.94 |
| D594G | 93 | 12.52 |
| N581S | 93 | 12.52 |
| N581I | 63 | 8.48 |
| G596R | 48 | 6.46 |
| G466A | 37 | 4.98 |
| G466E | 34 | 4.58 |
| G466R | 29 | 3.9 |
| N581Y | 27 | 3.63 |
| G469E | 13 | 1.75 |
| K483E | 13 | 1.75 |
| N581T | 12 | 1.62 |
| D594H | 11 | 1.48 |
| D594E | 9 | 1.21 |
| D594Y | 8 | 1.08 |
| G596C | 8 | 1.08 |
| G596V | 6 | 0.81 |
| F595L | 5 | 0.67 |
| D594A | 3 | 0.4 |
| F247L | 3 | 0.4 |
| G596D | 2 | 0.27 |
| S467L | 2 | 0.27 |
| D287H | 1 | 0.13 |
| D594V | 1 | 0.13 |
| N581K | 1 | 0.13 |
| Total (distinct) | 743 | 100 |

Frequency and percentage of patients with specific BRAF Class III alterations, with D594N (15.75%) and G466V (14.94%) being the most frequent.

## Supplementary Table 6. Clinical Characteristics

| Parameter | | BRAF Class | | | Pairwise Chi-squared test with Holm adj. *P*-value | | |
| --- | --- | --- | --- | --- | --- | --- | --- |
|  |  | Class I | Class II | Class III | I vs II | I vs III | II vs III |
|  | *N* | 845 | 810 | 743 |  |  |  |
| Age |  |  |  |  |  |  |  |
|  | Mean | 68.61 | 70.1 | 69.37 |  |  |  |
|  | Std Deviation | 11.01 | 9.7 | 10.47 |  |  |  |
|  | Median | 70 | 70 | 70 |  |  |  |
|  | Min | 23 | 42 | 41 |  |  |  |
|  | Max | 85 | 85 | 85 |  |  |  |
| Sex |  |  |  |  | * | * | ns |
| Female | Frequency | 476 | 410 | 371 | 0.0451 | 0.0373 | 0.827 |
|  | Percent (%) | 56.33 | 50.62 | 49.93 |  |  |  |
| Male | Frequency | 369 | 400 | 372 |  |  |  |
|  | Percent (%) | 43.67 | 49.38 | 50.07 |  |  |  |
| Tobacco Use |  |  |  |  | **** | **** | ns |
| Ever Tobacco Product User | Frequency | 462 | 559 | 509 | 8.18×10^-9^ | 4.52×10^-8^ | 0.873 |
|  | Percent (%) | 54.67 | 69.01 | 68.51 |  |  |  |
| Unknown | Frequency | 383 | 251 | 234 |  |  |  |
|  | Percent (%) | 45.33 | 30.99 | 31.49 |  |  |  |

Patients with multiple BRAF alterations across functional classes were excluded from the summary of characteristics. Two-sided pairwise Chi-squared test with Holm *P*-value adjustment was used to compare frequencies across BRAF Classes. **P <* 0.05, ***P <* 0.01, ****P <* 0.001, *****P <* 0.0001.

## Supplementary Table 7. Residues contacting exarafenib in the AlphaFold-predicted ARAF–KSR1 complex

| Chain | Residue name | Residue number | Min Distance (Å) |
| --- | --- | --- | --- |
| ARAF | VAL | 324 | 3.86 |
| ARAF | ALA | 334 | 2.6 |
| ARAF | VAL | 335 | 3.4 |
| ARAF | LYS | 336 | 2.57 |
| ARAF | GLU | 354 | 2.99 |
| ARAF | VAL | 357 | 3.6 |
| ARAF | LEU | 358 | 2.2 |
| ARAF | THR | 361 | 2.37 |
| ARAF | ILE | 366 | 3.11 |
| ARAF | LEU | 367 | 3.68 |
| ARAF | ILE | 380 | 2.94 |
| ARAF | ILE | 381 | 4.77 |
| ARAF | THR | 382 | 2.75 |
| ARAF | GLN | 383 | 1.62 |
| ARAF | TRP | 384 | 2.19 |
| ARAF | CYS | 385 | 2.14 |
| ARAF | LEU | 420 | 2.72 |
| ARAF | ILE | 425 | 4.47 |
| ARAF | PHE | 436 | 2.62 |
| ARAF | ILE | 445 | 4.41 |
| ARAF | GLY | 446 | 4.12 |
| ARAF | ASP | 447 | 3.41 |
| ARAF | PHE | 448 | 0.7 |
| ARAF | GLY | 449 | 3.57 |
| ARAF | ALA | 451 | 4.23 |
| KSR1 | PRO | 408 | 4.21 |
| KSR1 | SER | 409 | 1.6 |
| KSR1 | ASP | 410 | 2.46 |
| KSR1 | ILE | 411 | 0.67 |
| KSR1 | ASN | 412 | 1.88 |
| KSR1 | ASN | 413 | 4.79 |

## Supplementary Table 8. Antibody List

| **Protein** |  | **Site** | **Product No.** | **Manufacturer** | **Species** |
| --- | --- | --- | --- | --- | --- |
| pan-RAS | total |  | AESA02 | Cytoskeleton | Mouse |
| ARAF | total |  | sc-166771 | Santa Cruz | Mouse |
|  | total |  | sc-408 | Santa Cruz | Rabbit |
| BRAF | total |  | sc-5284 | Santa Cruz | Mouse |
|  | total |  | 14814 | Cell Signaling Technology | Rabbit |
| CRAF | total |  | BD-610152 | BD Biosciences | Mouse |
| KSR1 | total |  | MA5-53956 | Invitrogen | Rabbit |
| MEK | phospho | Ser217/221 | 9154 | Cell Signaling Technology | Rabbit |
|  | phospho | Ser218/222 | sc-81503 | Santa Cruz | Mouse |
|  | total |  | BD-610122 | BD Biosciences | Mouse |
|  | total |  | 9122 | Cell Signaling Technology | Rabbit |
| ERK | phospho | Thr202/Tyr204 | 9101 | Cell Signaling Technology | Rabbit |
|  | total |  | 9102 | Cell Signaling Technology | Rabbit |
| RSK | phospho | Thr359/Ser363 | 9344 | Cell Signaling Technology | Rabbit |
|  | total |  | 9355 | Cell Signaling Technology | Rabbit |
| AKT | phospho | Ser473 | 4060 | Cell Signaling Technology | Rabbit |
|  | total |  | 4691 | Cell Signaling Technology | Rabbit |
| PRAS40 | phospho | Thr246 | 13175 | Cell Signaling Technology | Rabbit |
|  | total |  | 2610 | Cell Signaling Technology | Rabbit |
| DUSP6 | total |  | 39441 | Cell Signaling Technology | Rabbit |
| Cyclin D1 | total |  | 55506 | Cell Signaling Technology | Rabbit |
| EphA2 | total |  | 6997 | Cell Signaling Technology | Rabbit |
| BIM | total |  | 2933 | Cell Signaling Technology | Rabbit |
| EGFR | total |  | 4267 | Cell Signaling Technology | Rabbit |
| AXL | total |  | 8661 | Cell Signaling Technology | Rabbit |
| MET | total |  | 8198 | Cell Signaling Technology | Rabbit |
| GAPDH | total |  | sc-365062 | Santa Cruz | Mouse |
| CHK2 | phospho | Thr68 | 2661 | Cell Signaling Technology | Rabbit |
| H2A.X | phospho | Ser139 | 2577 | Cell Signaling Technology | Rabbit |
| PARP | total |  | 9542 | Cell Signaling Technology | Rabbit |

# Uncropped Blots

## Supplementary Figure 1b.


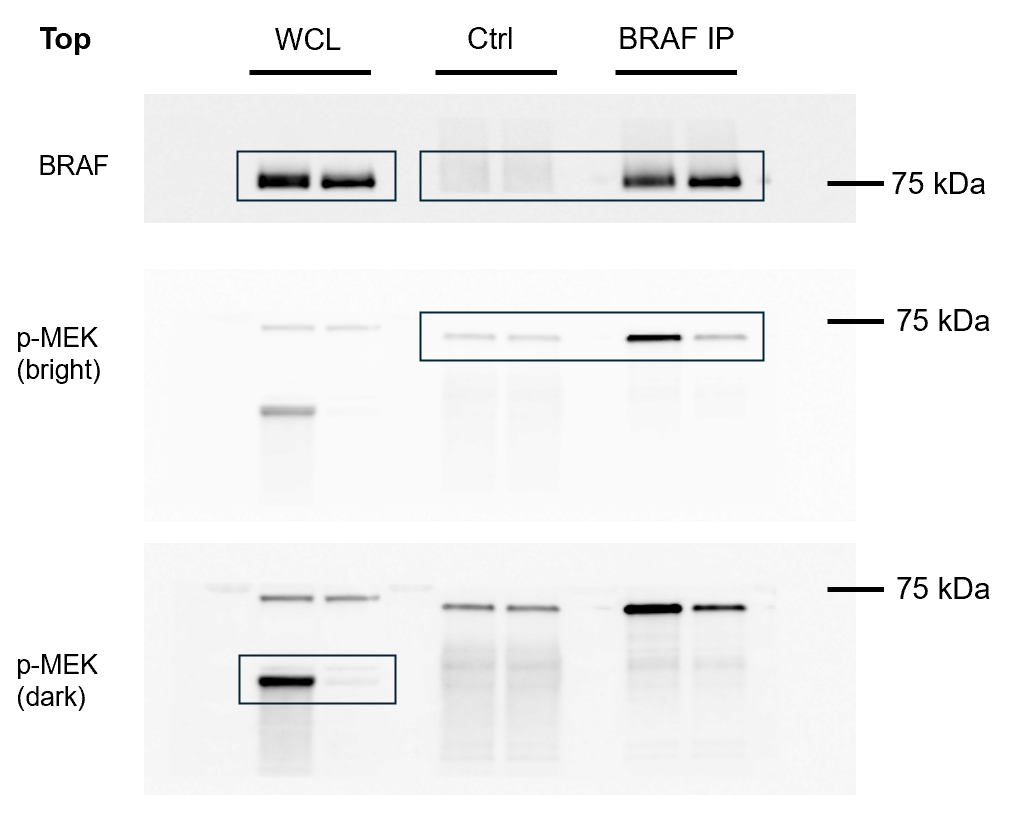

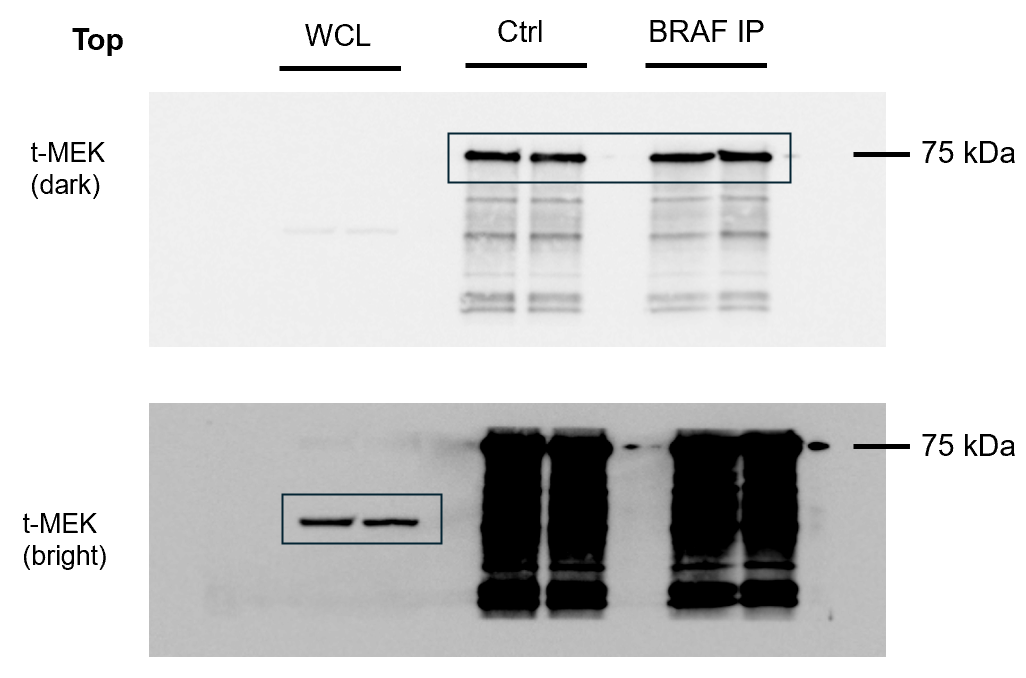


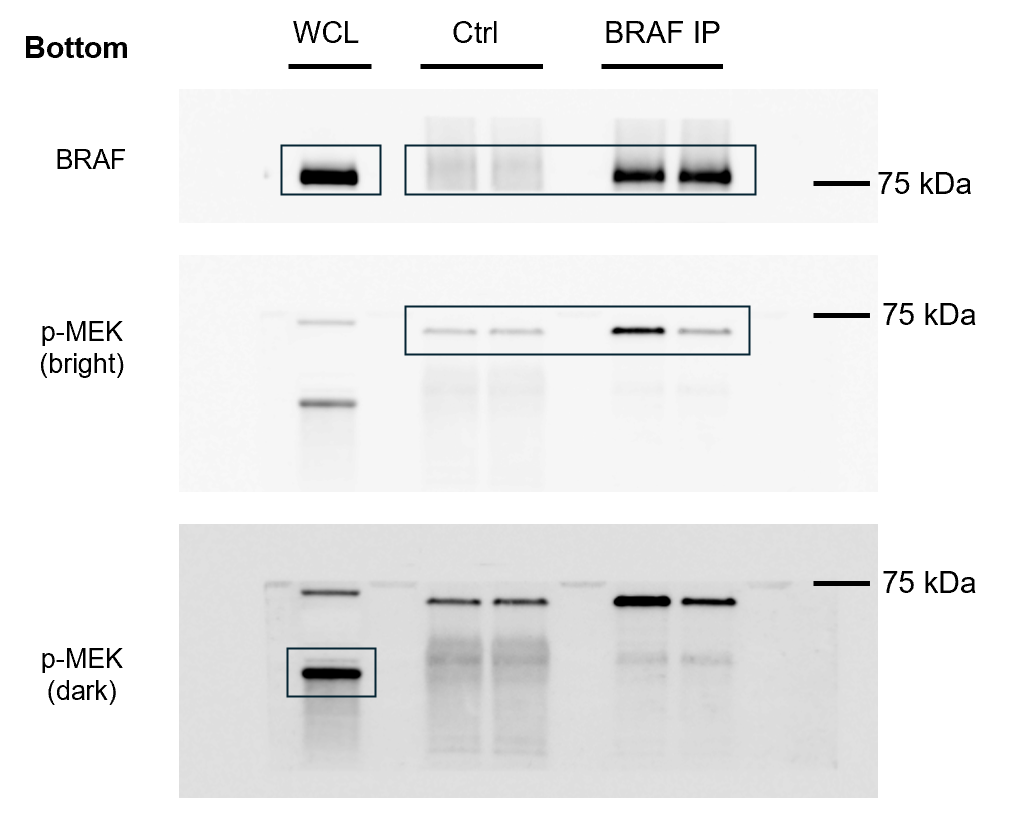

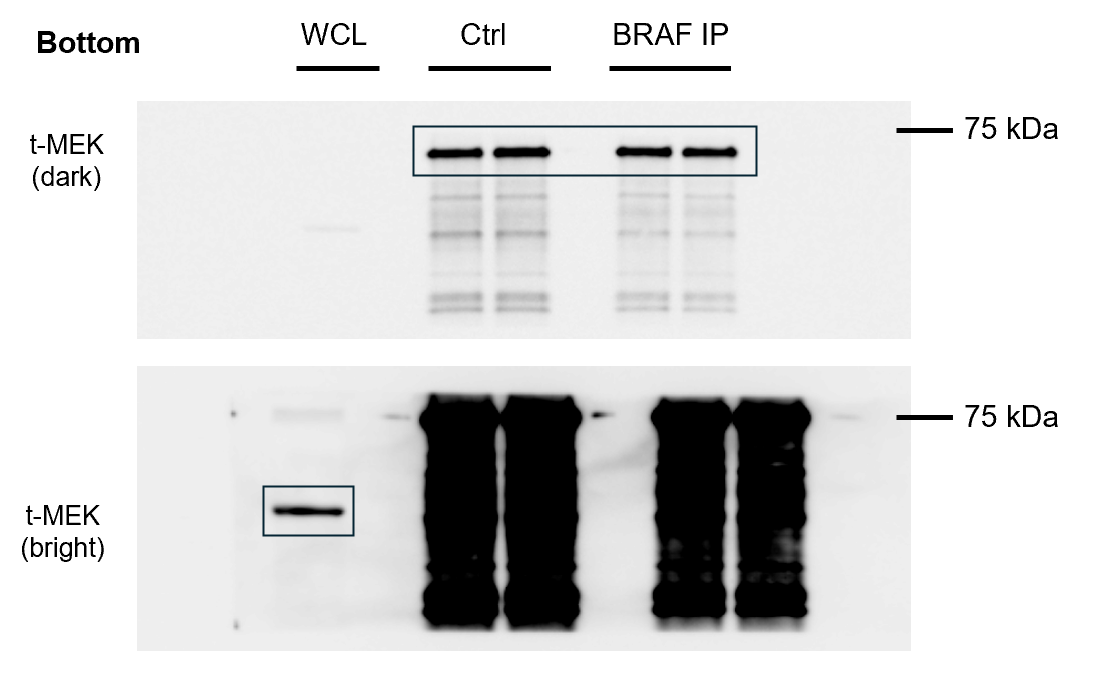


## Supplementary Figure 2b.


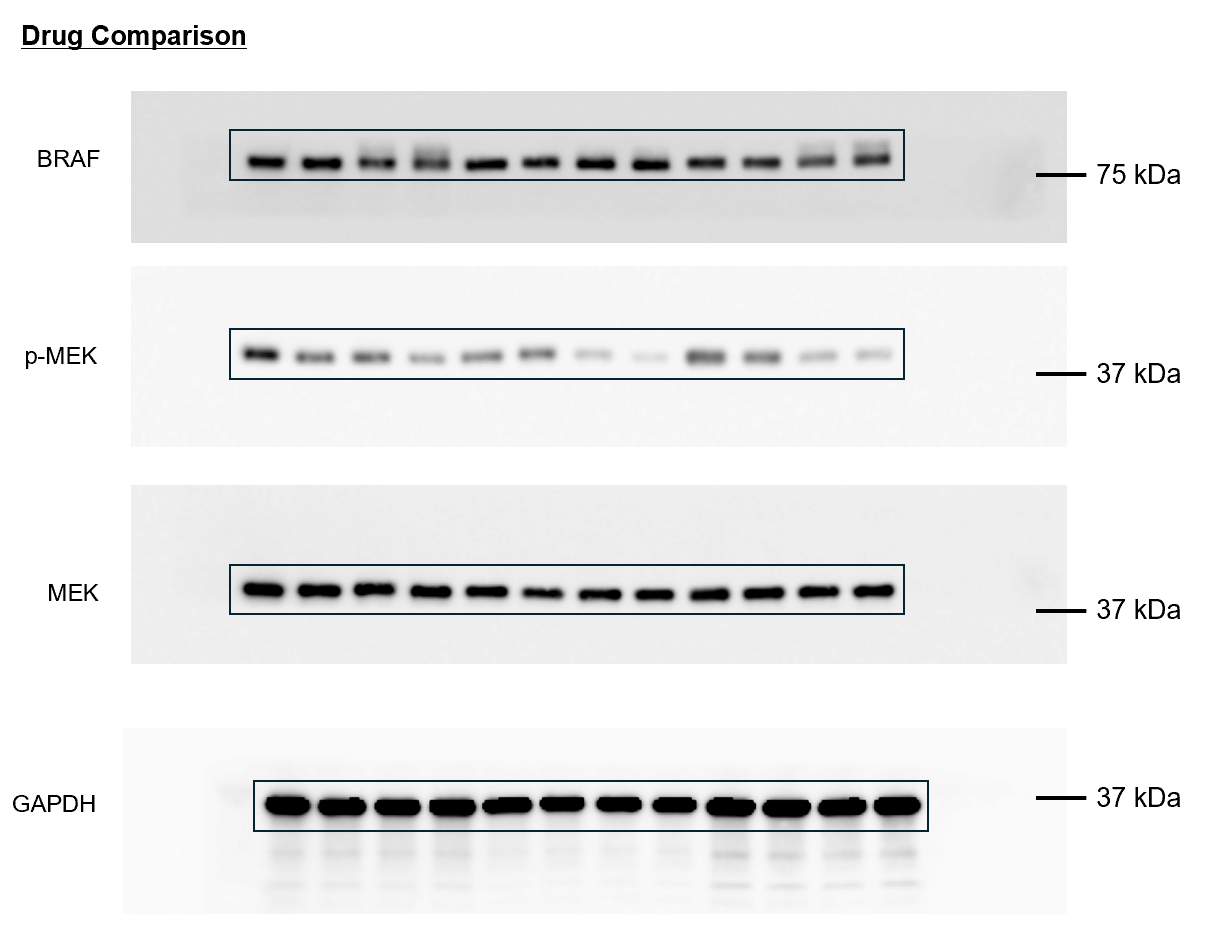

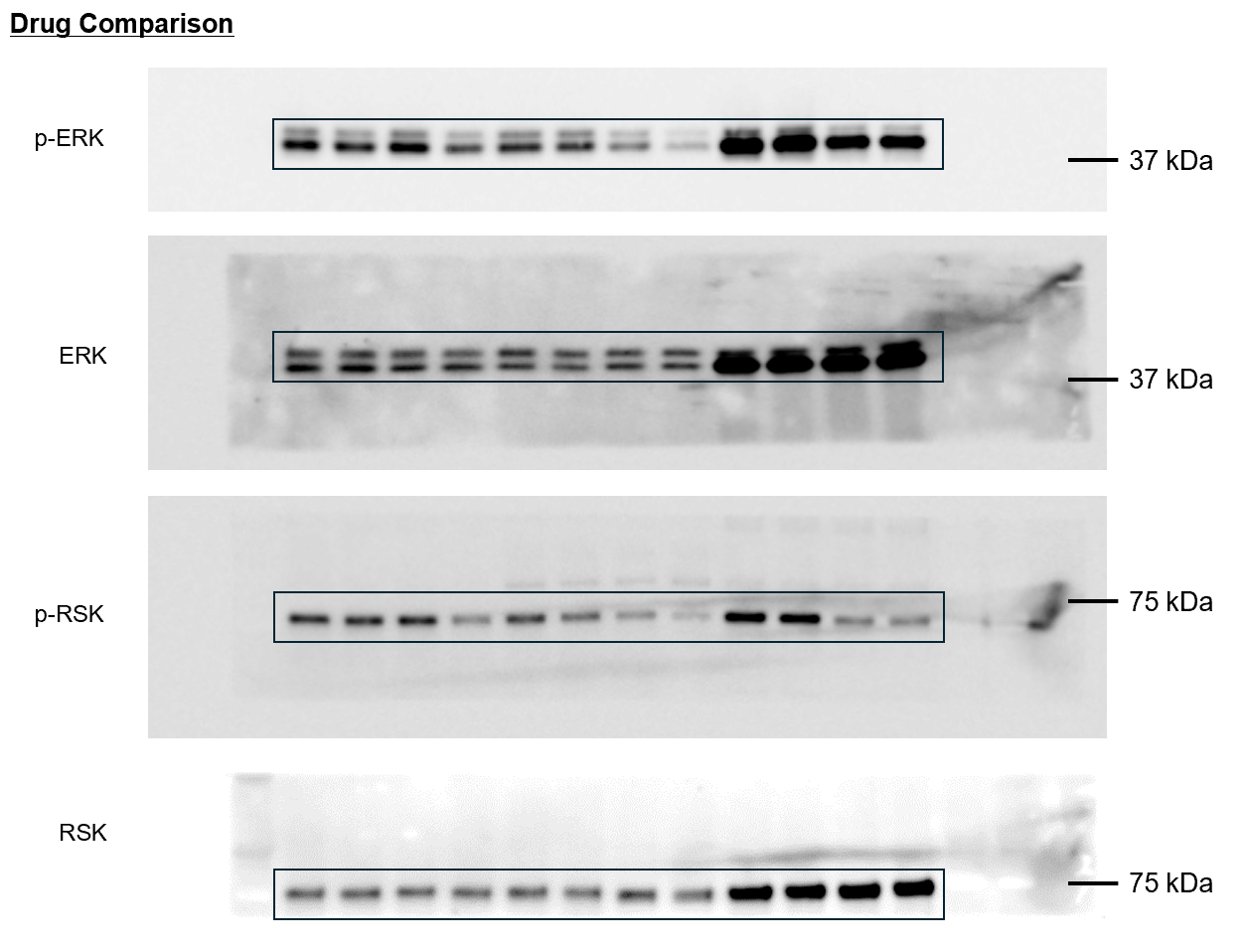


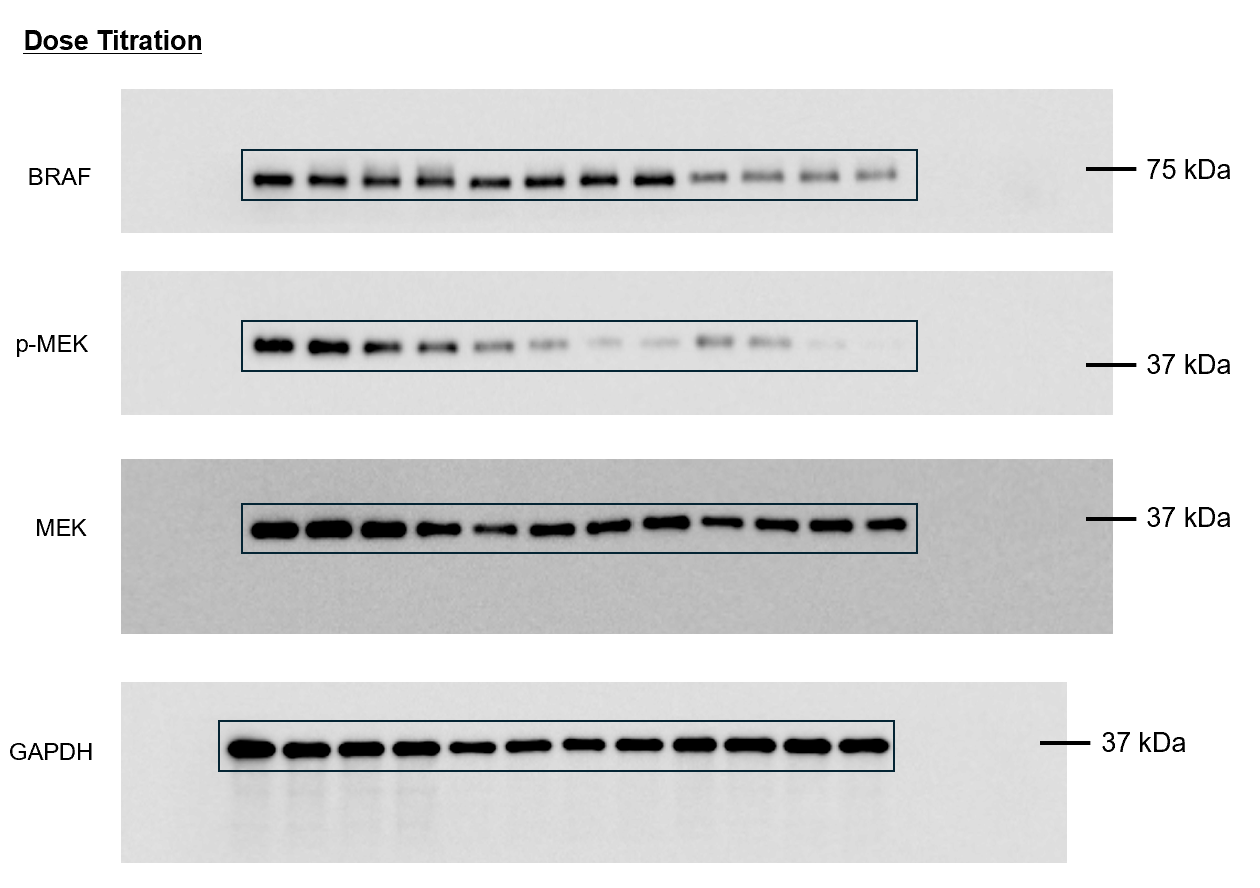

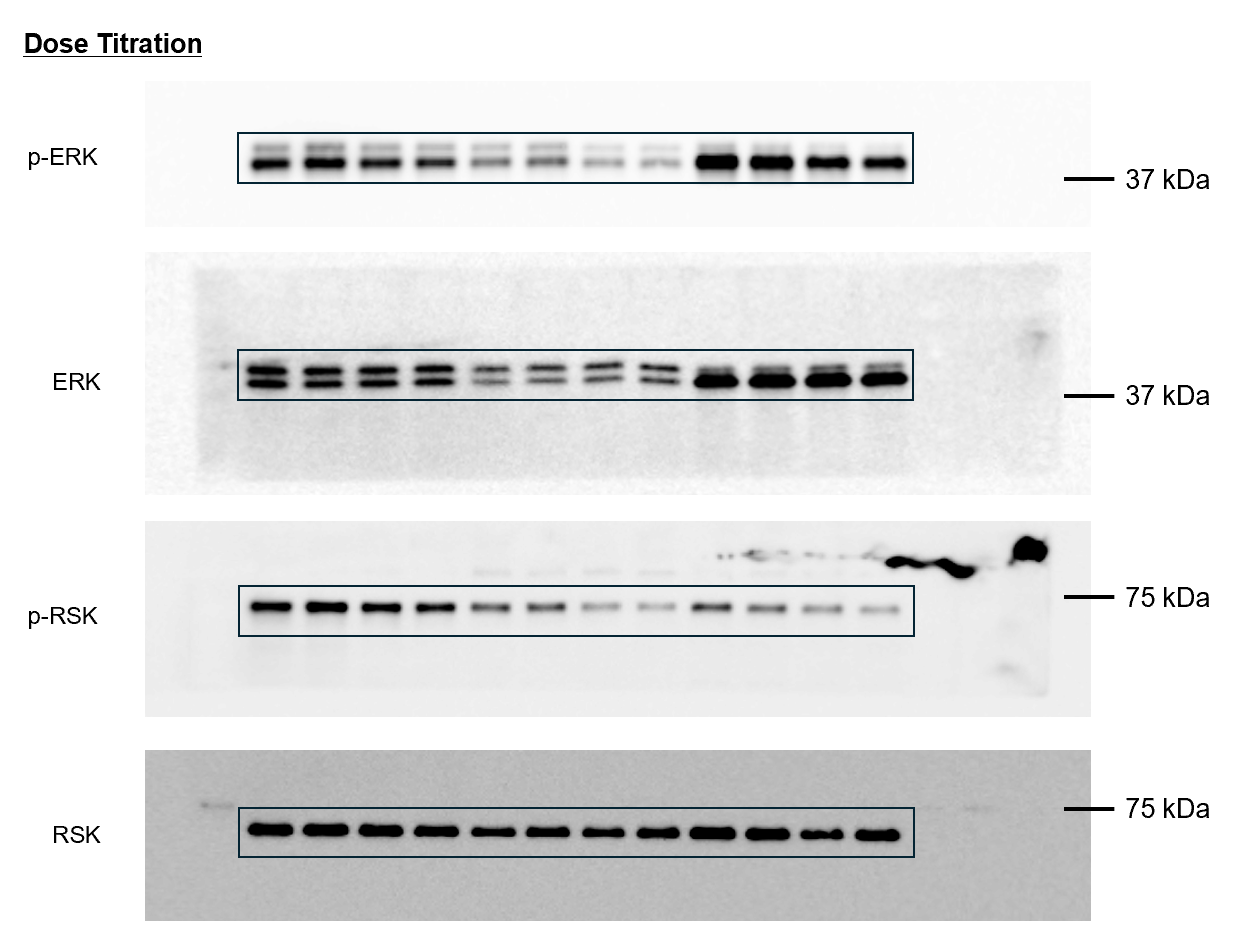


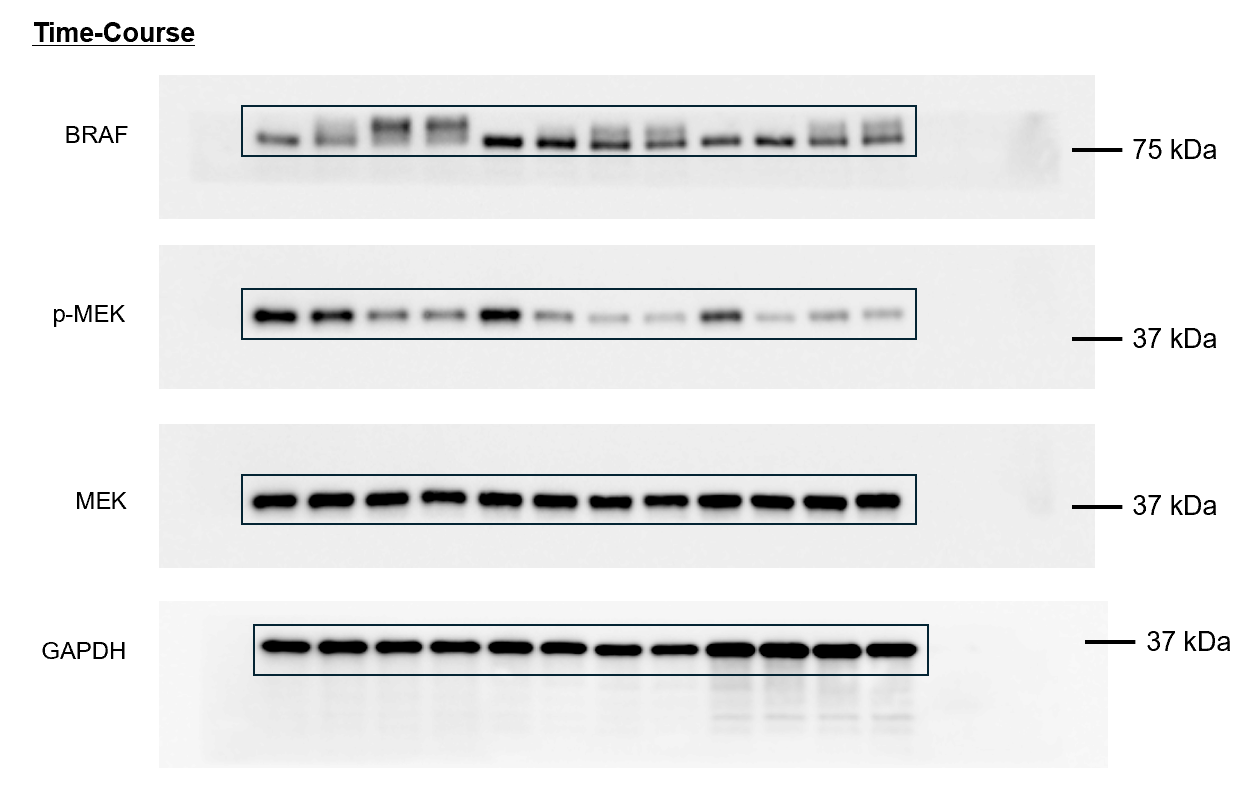

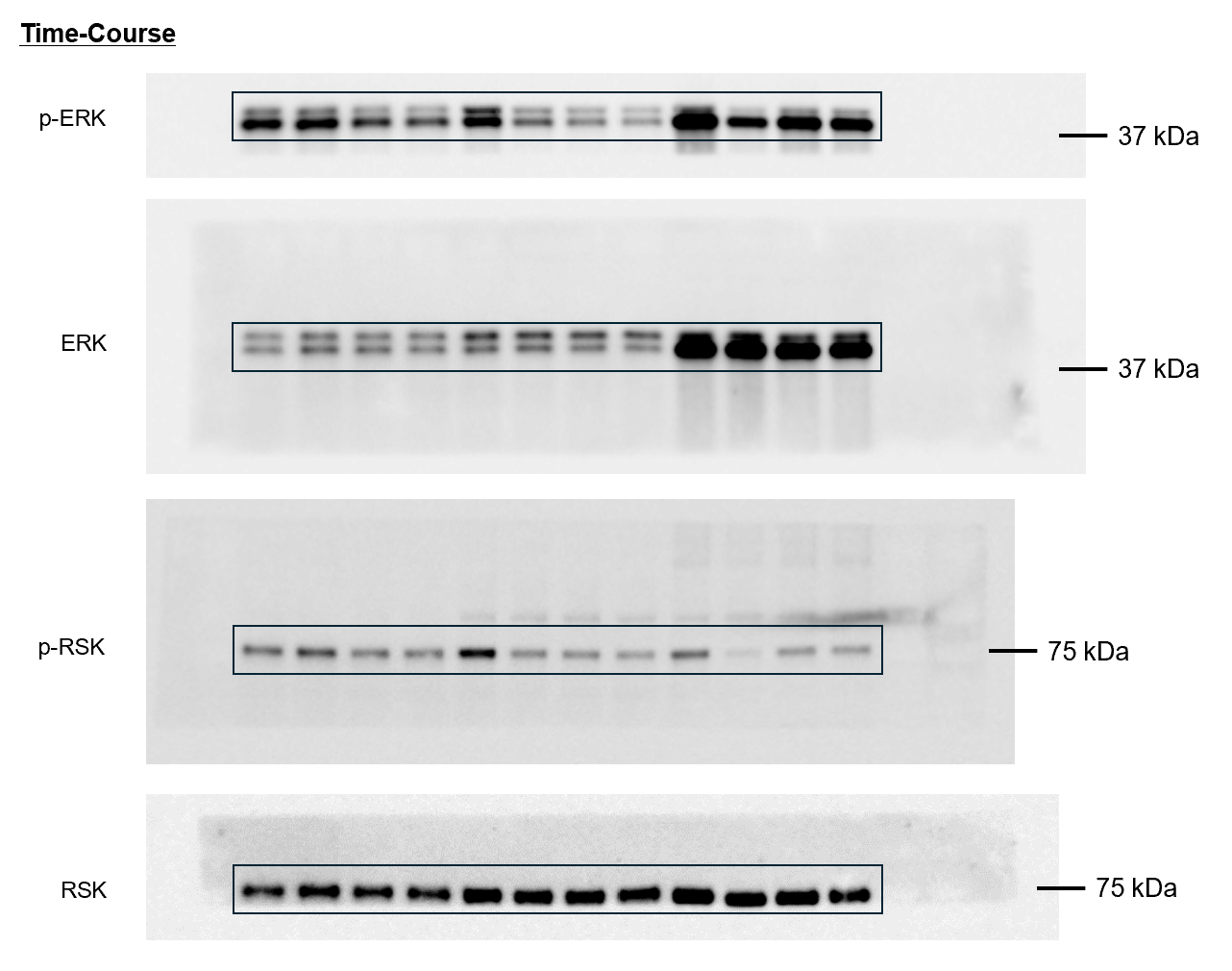


## Supplementary Figure 2e.


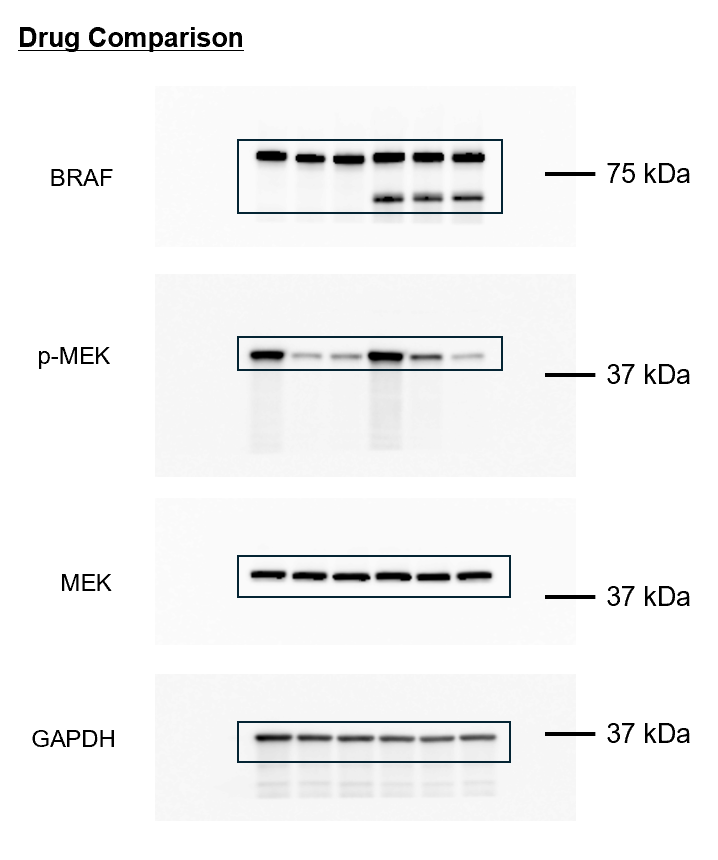

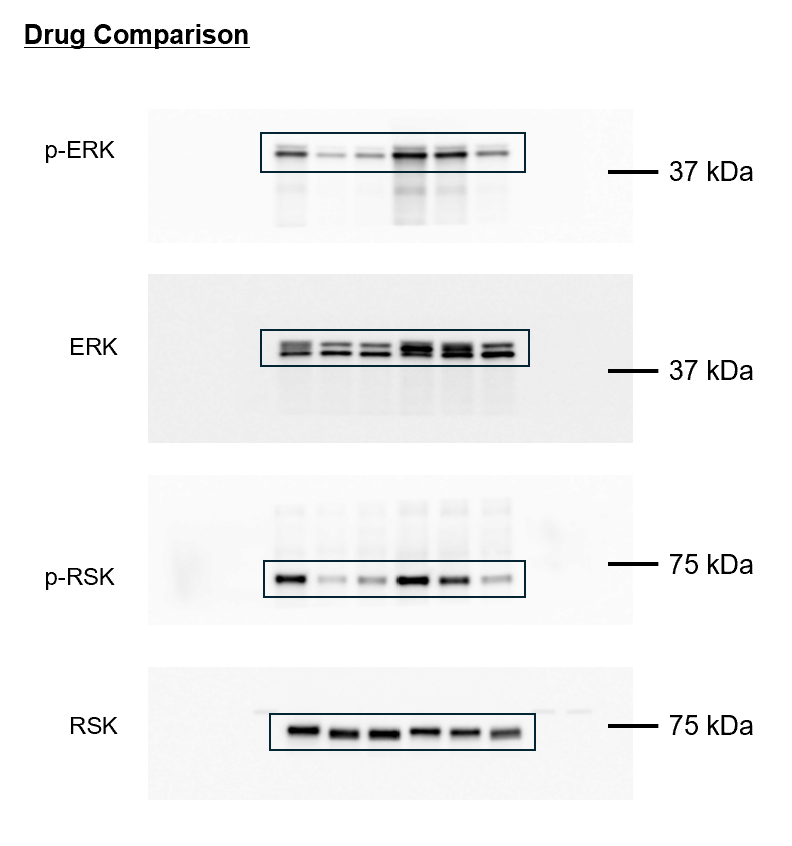


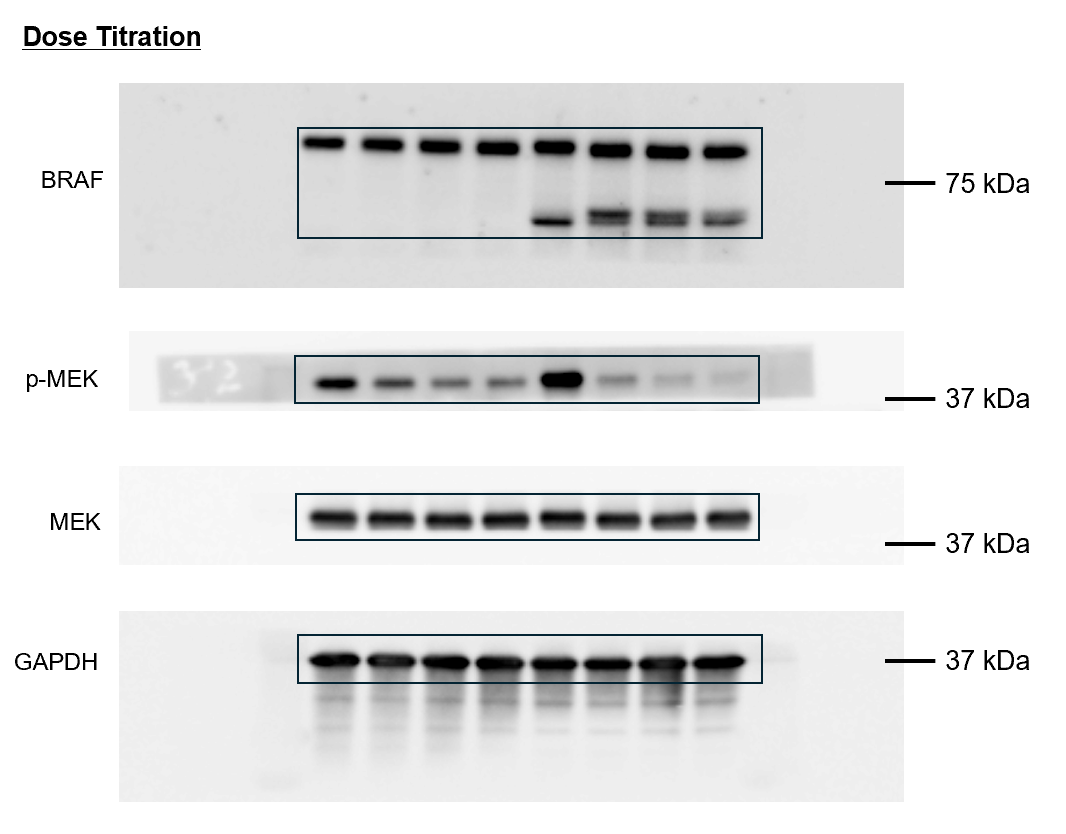
　
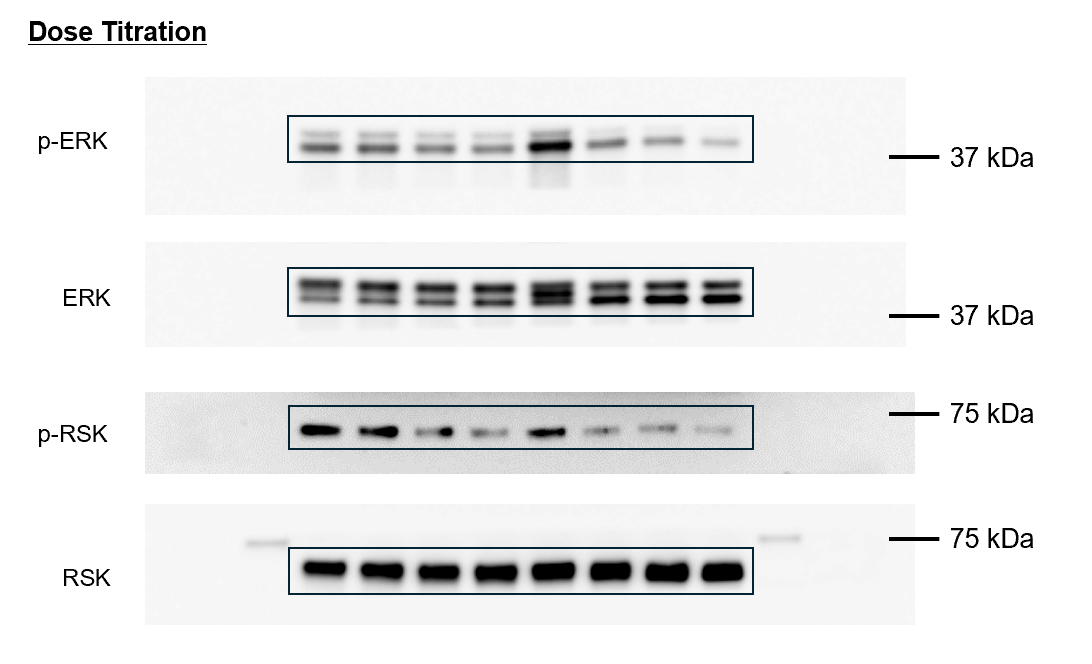


## Supplementary Figure 3d.


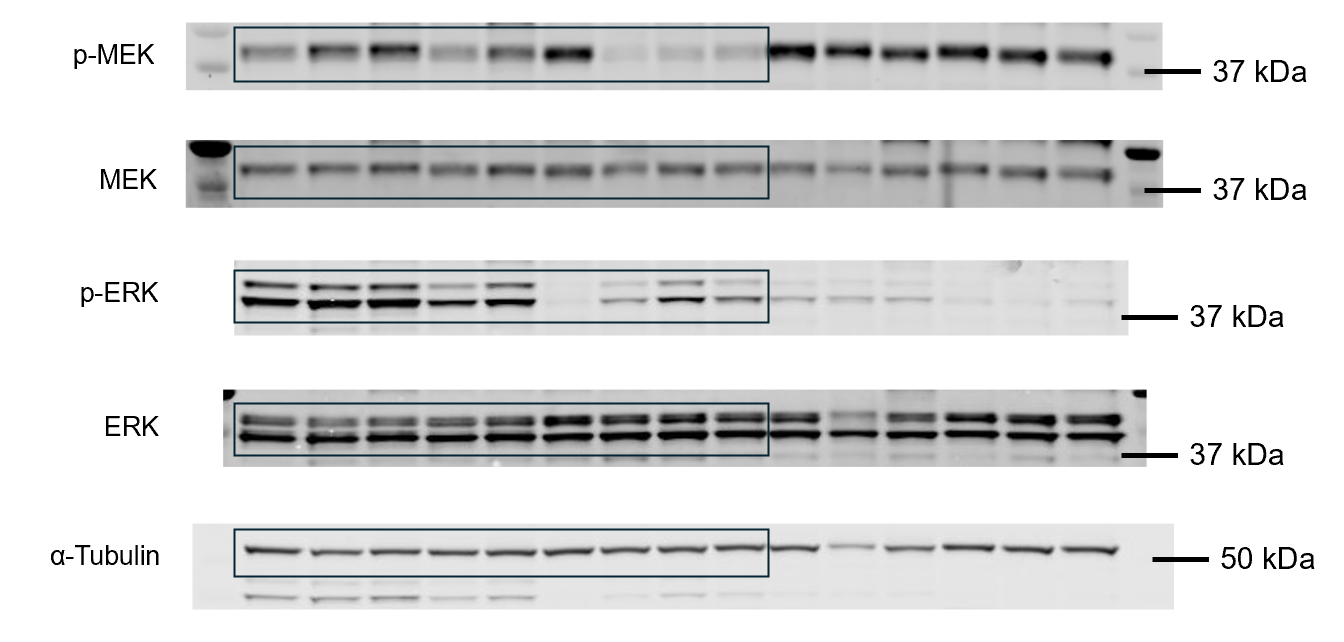


## Supplementary Figure 4c.


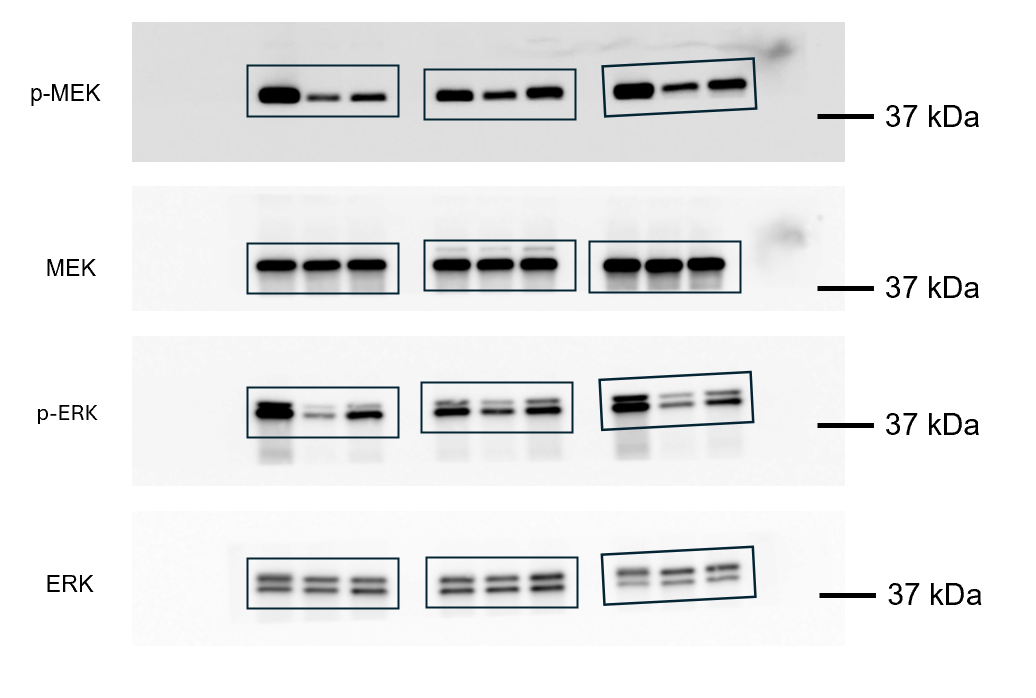

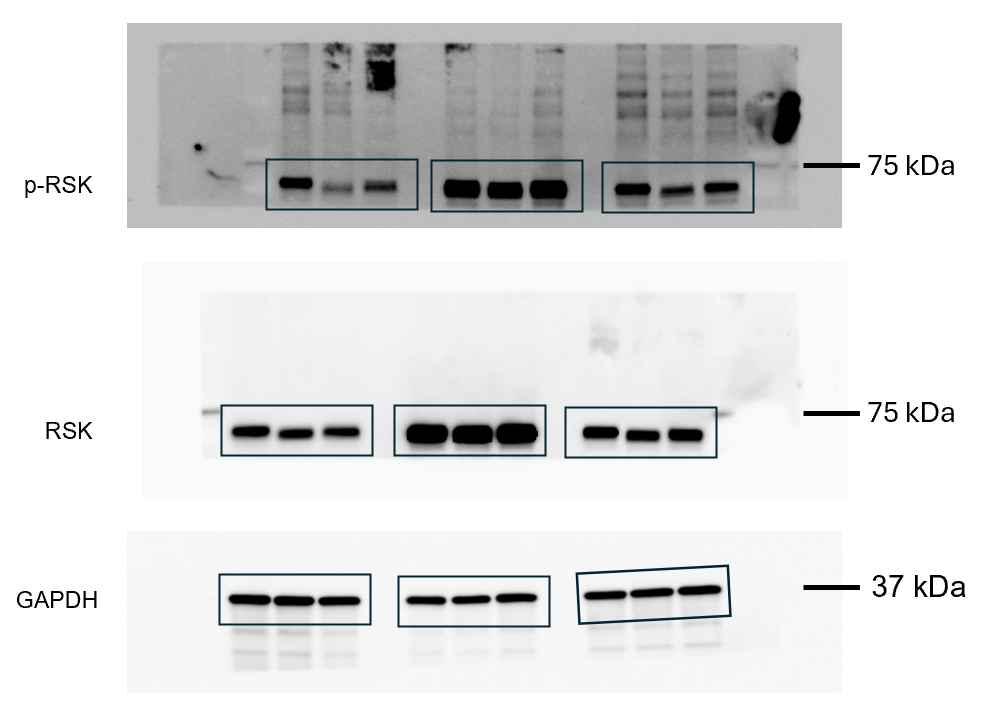


## Supplementary Figure 4d.


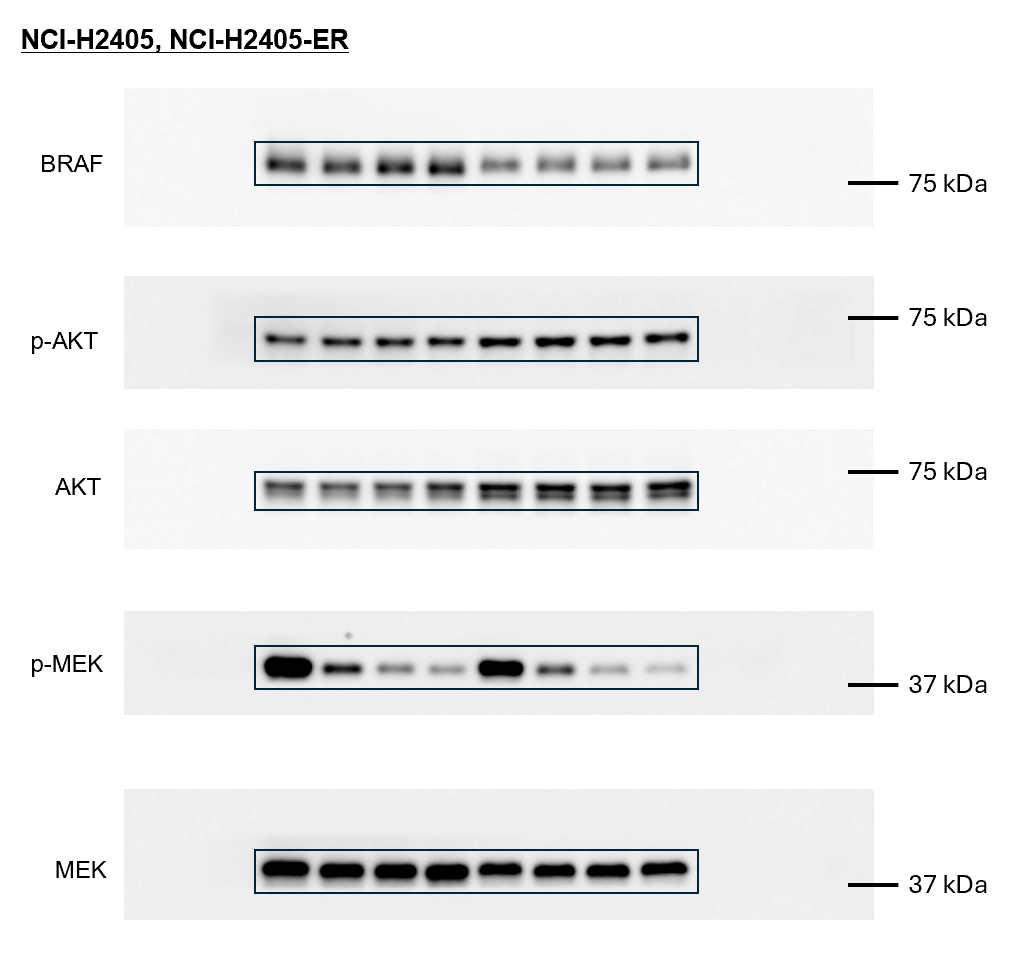

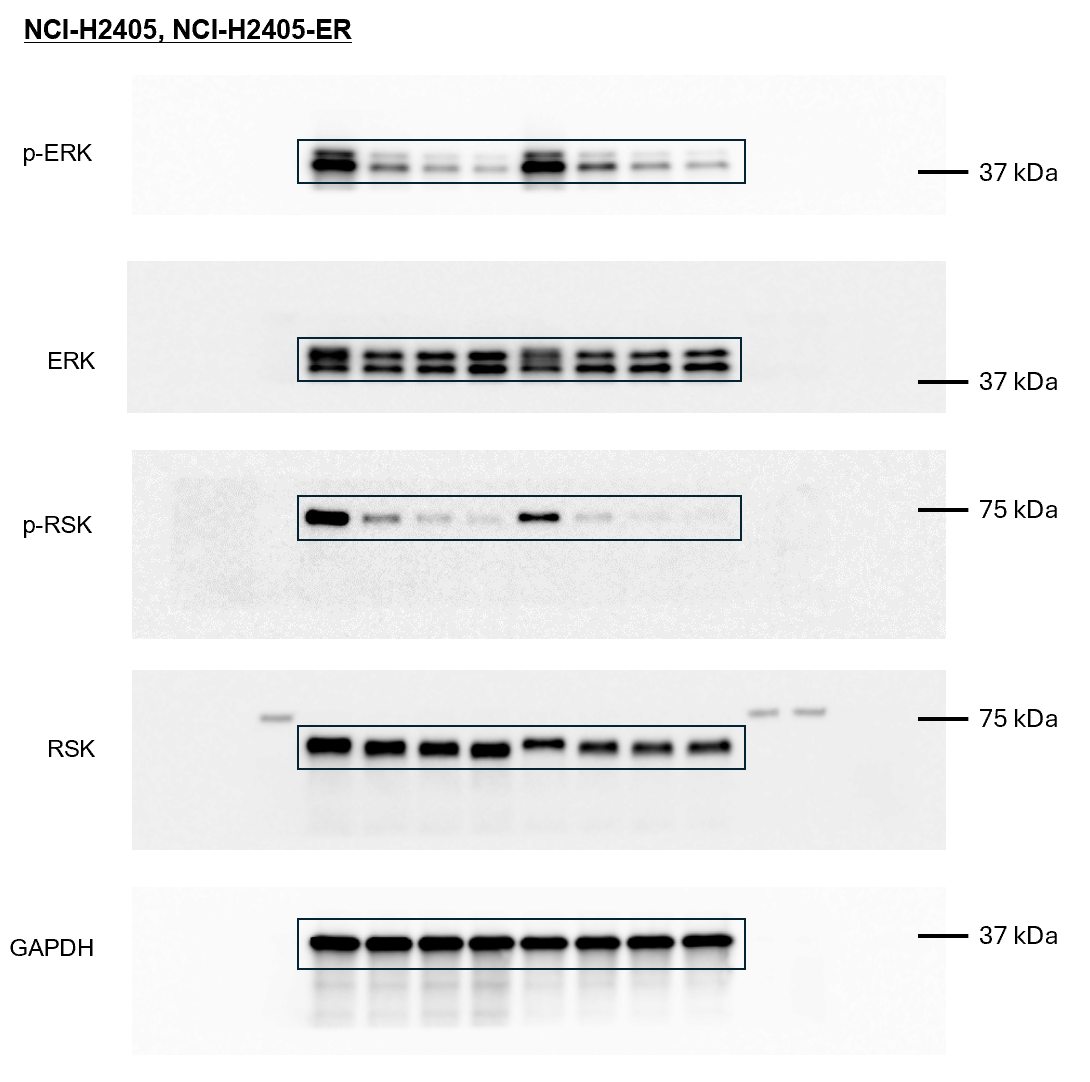


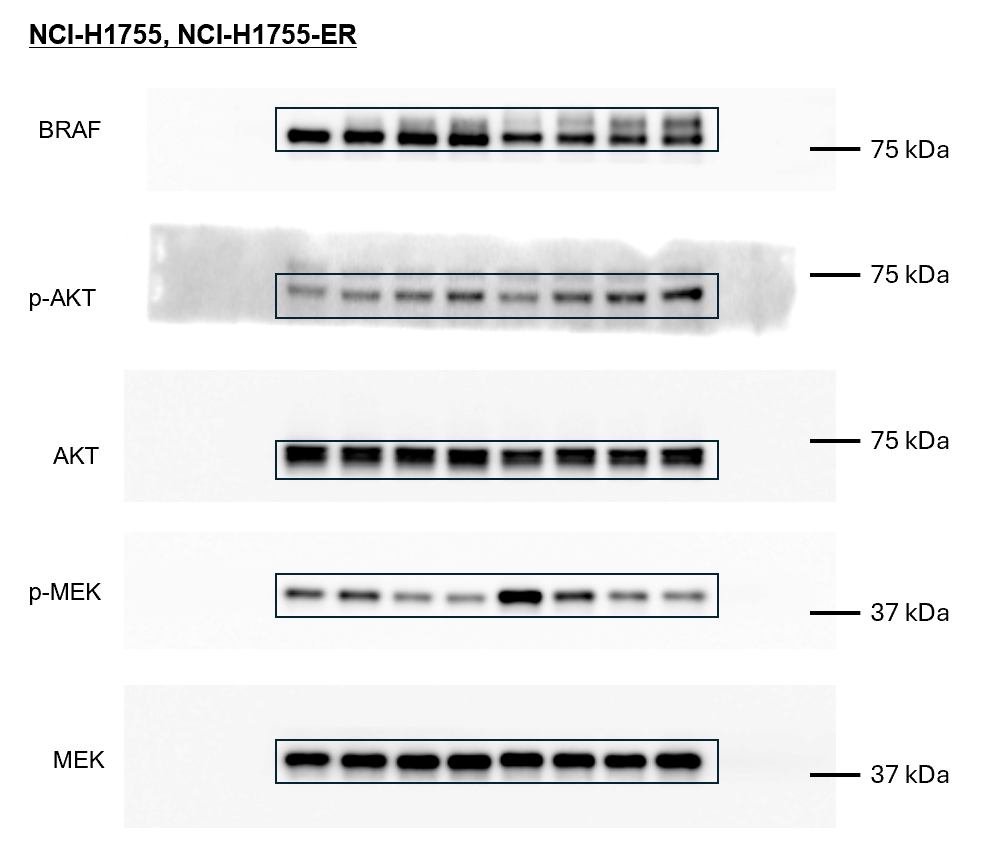

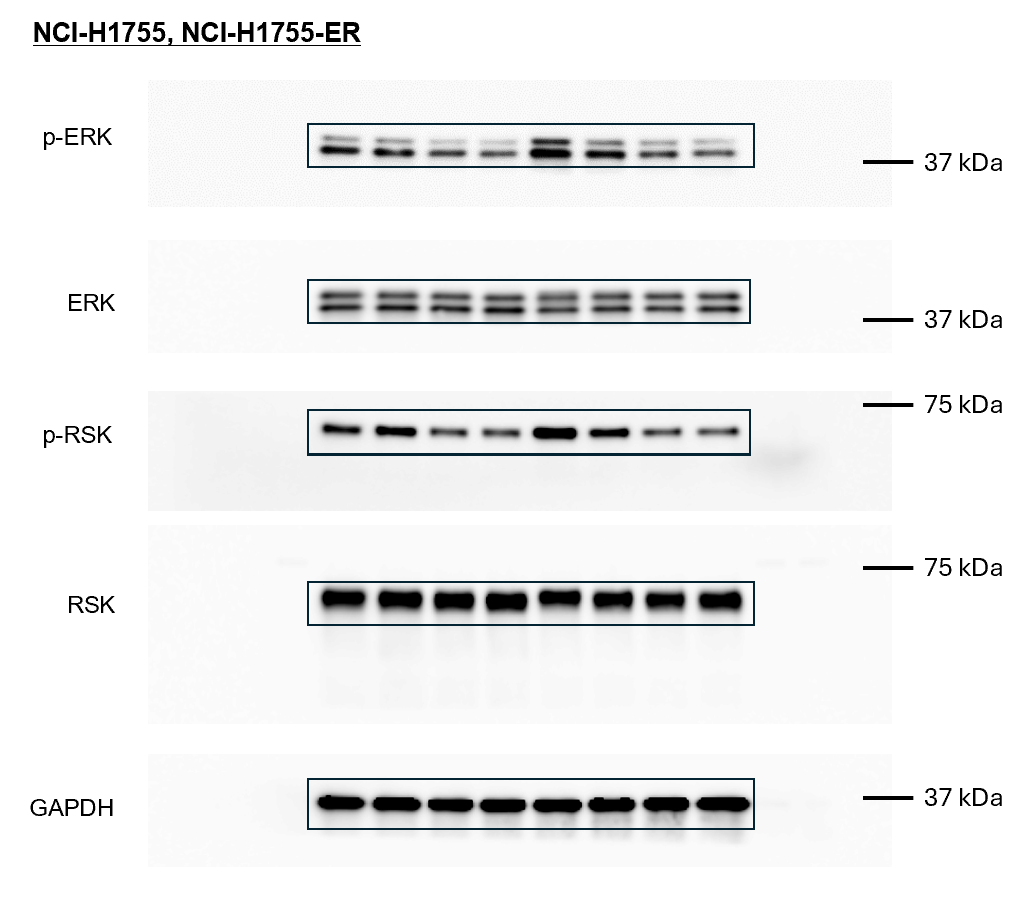


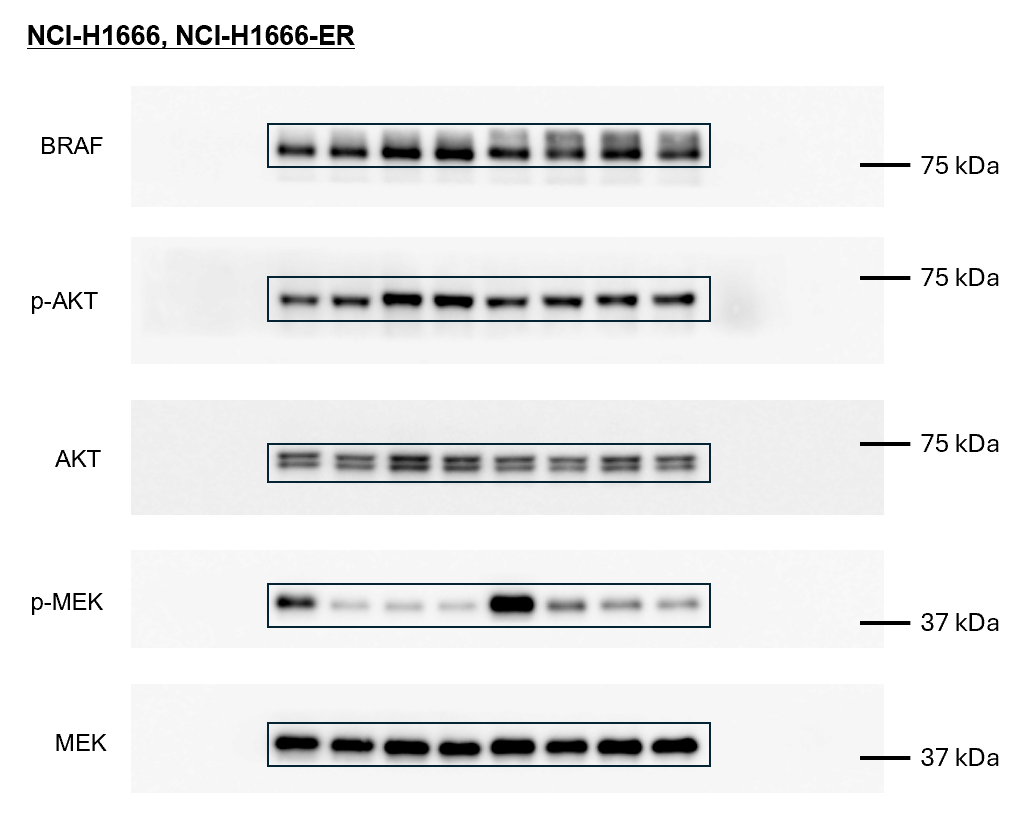

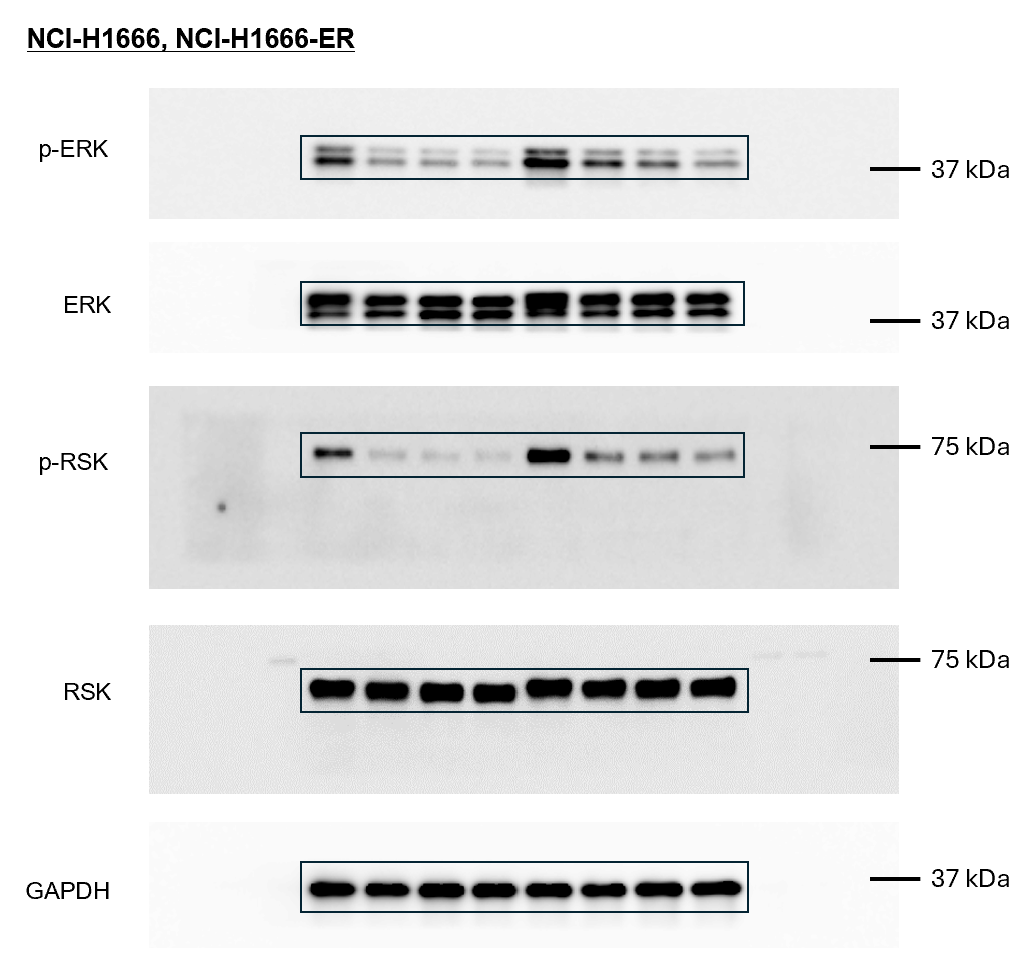


## Supplementary Figure 5a.


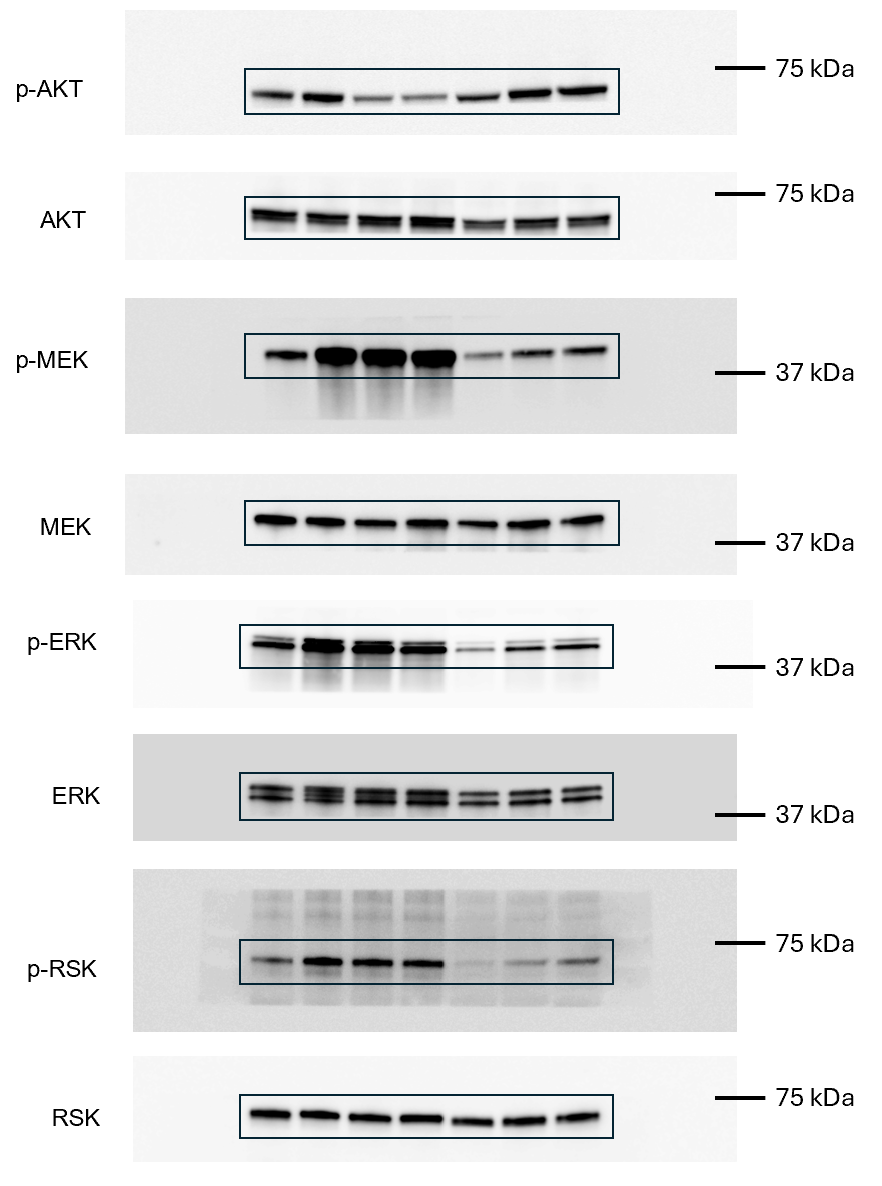

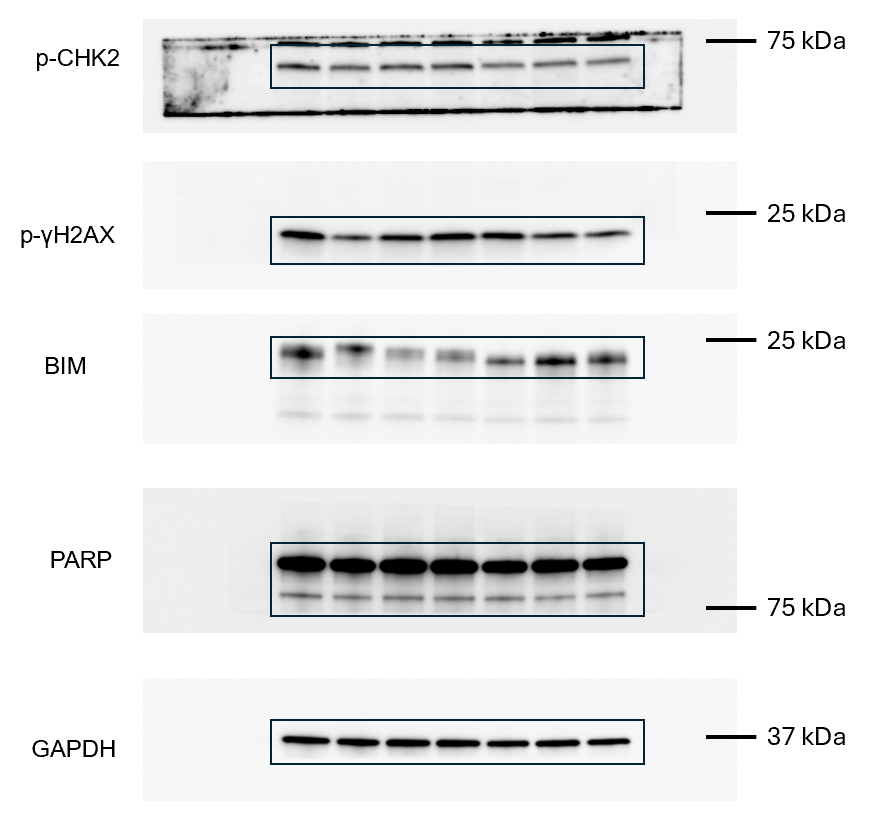


## Supplementary Figure 5c.


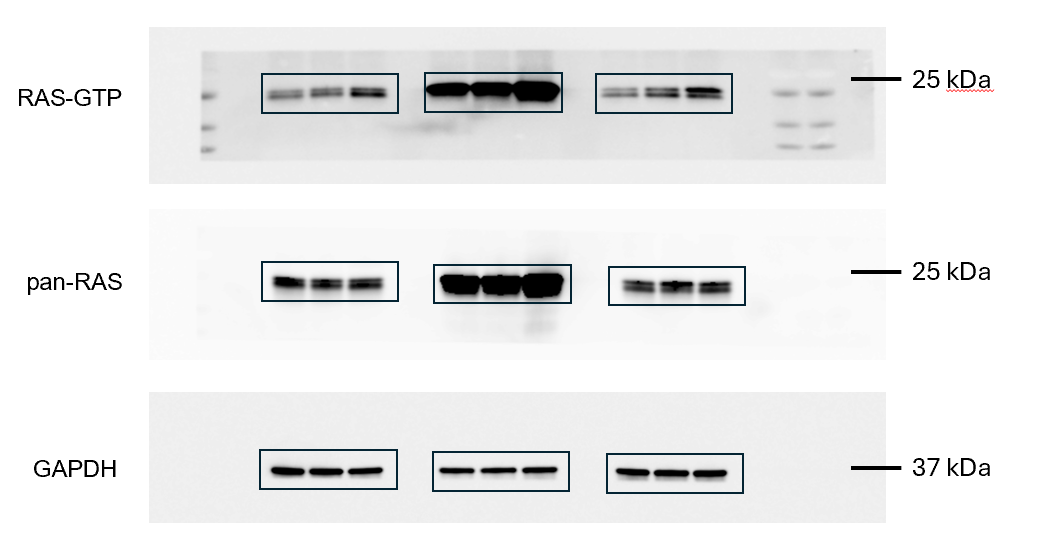


## Supplementary Figure 5d.


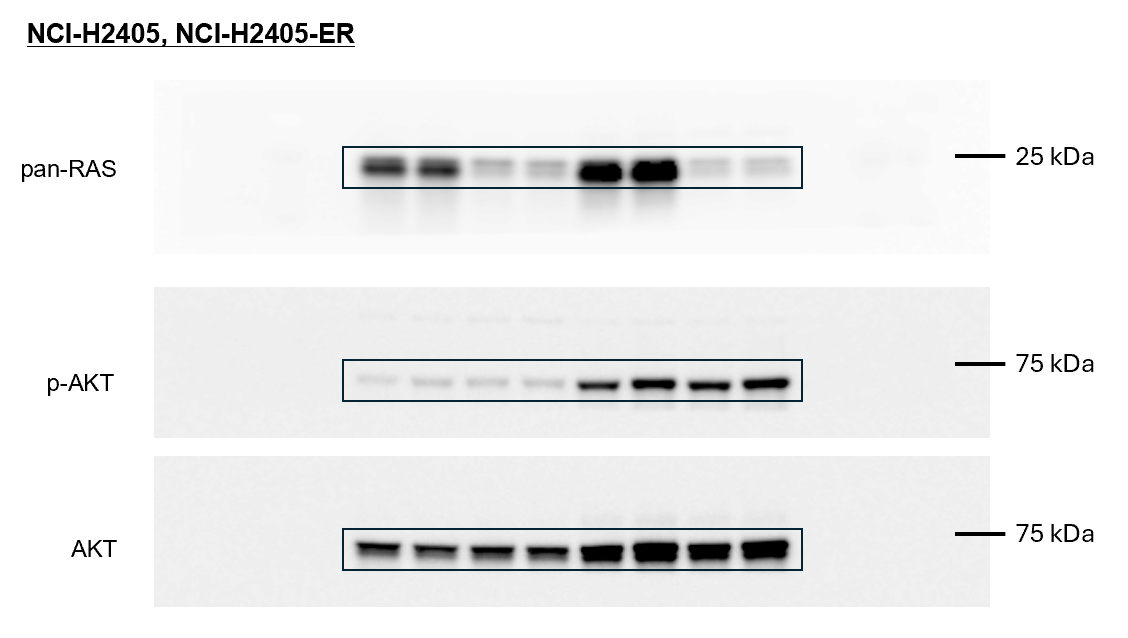

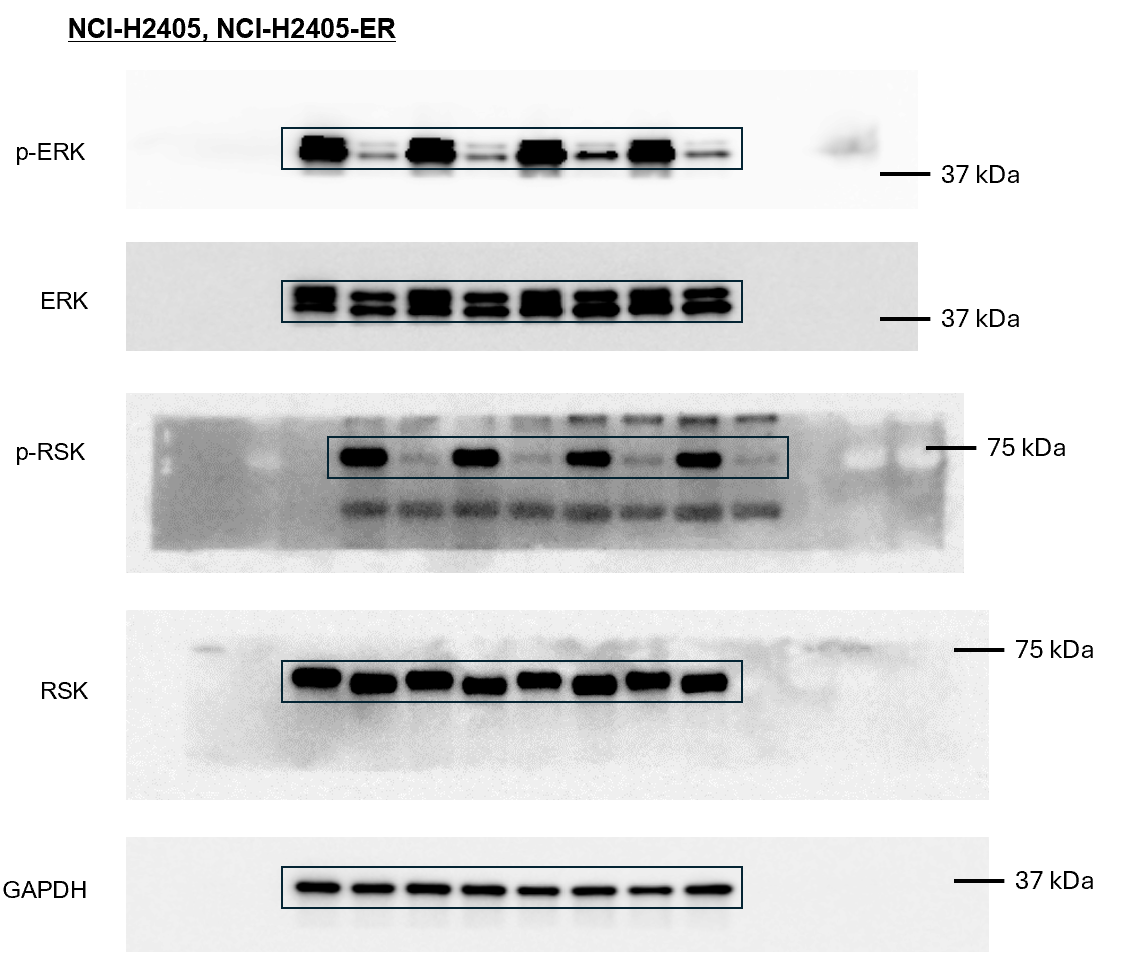


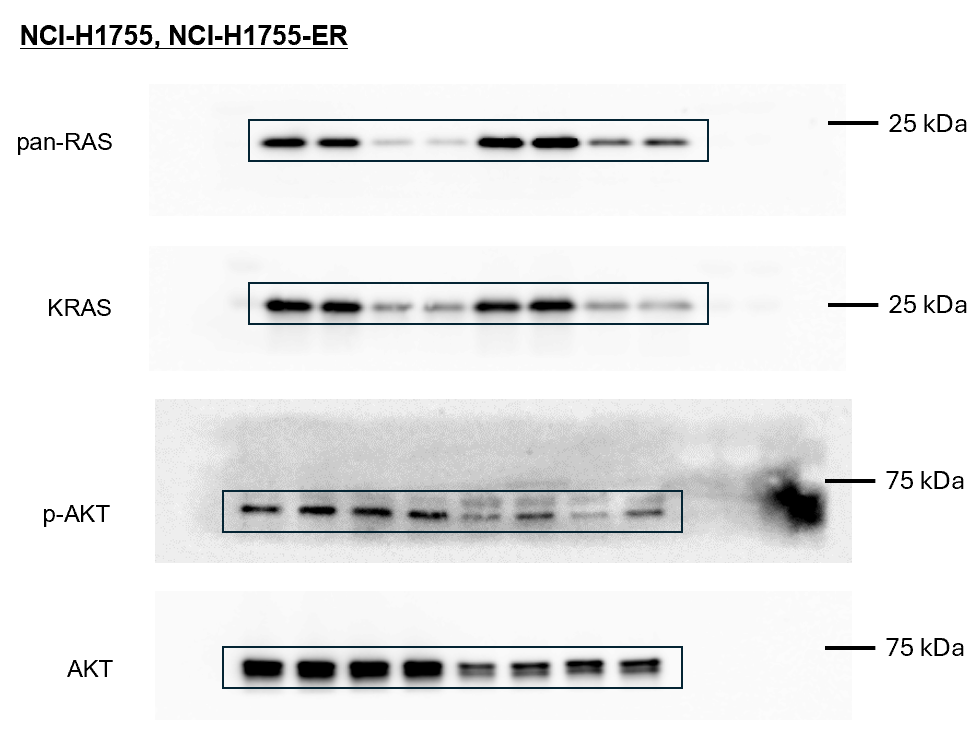

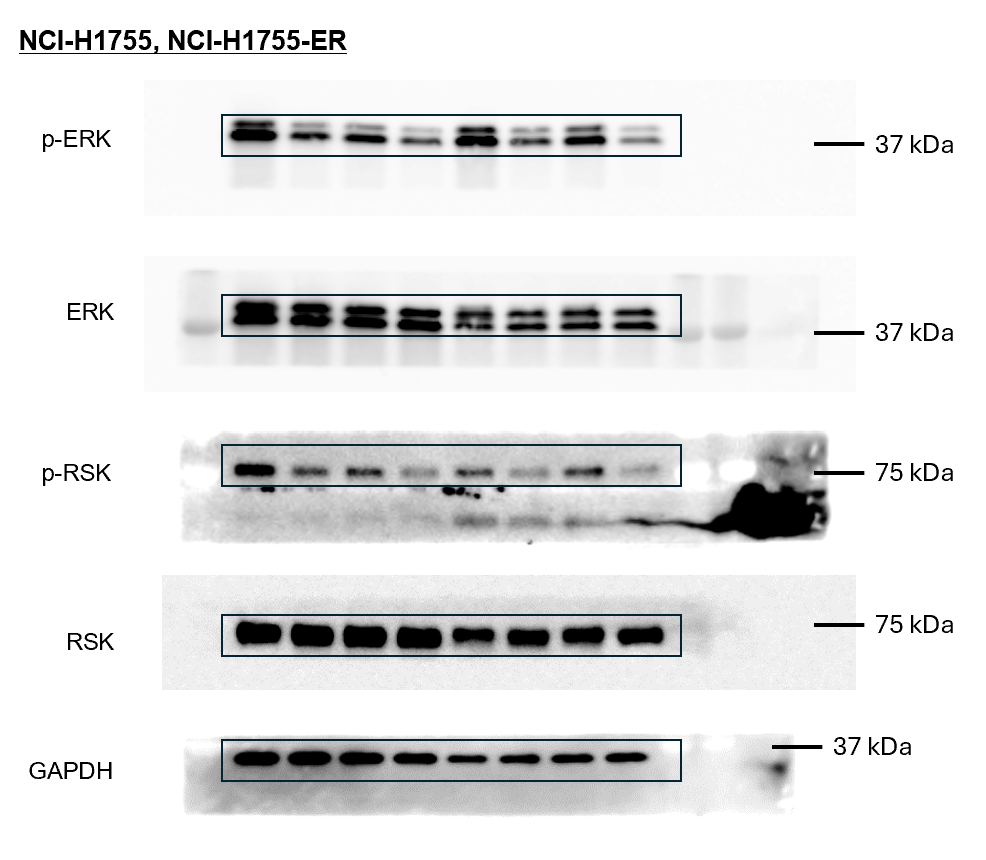


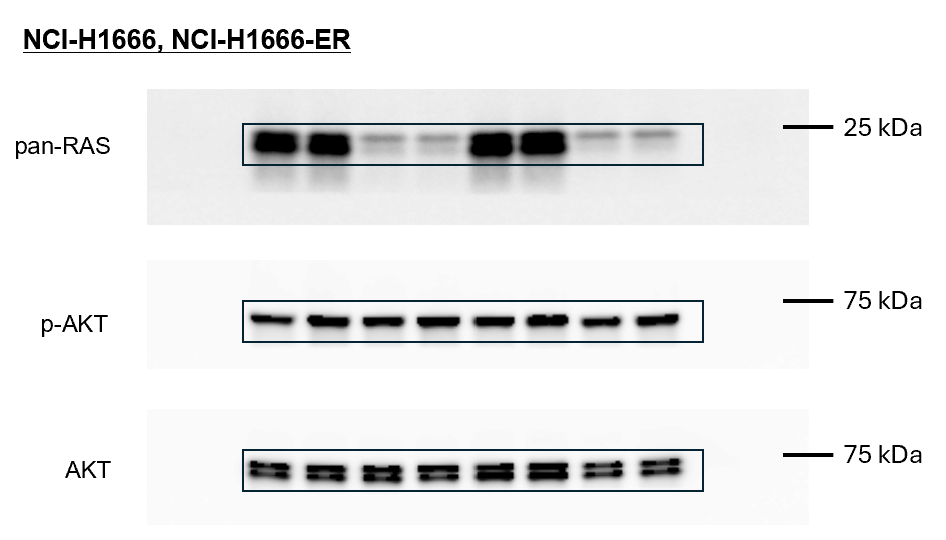

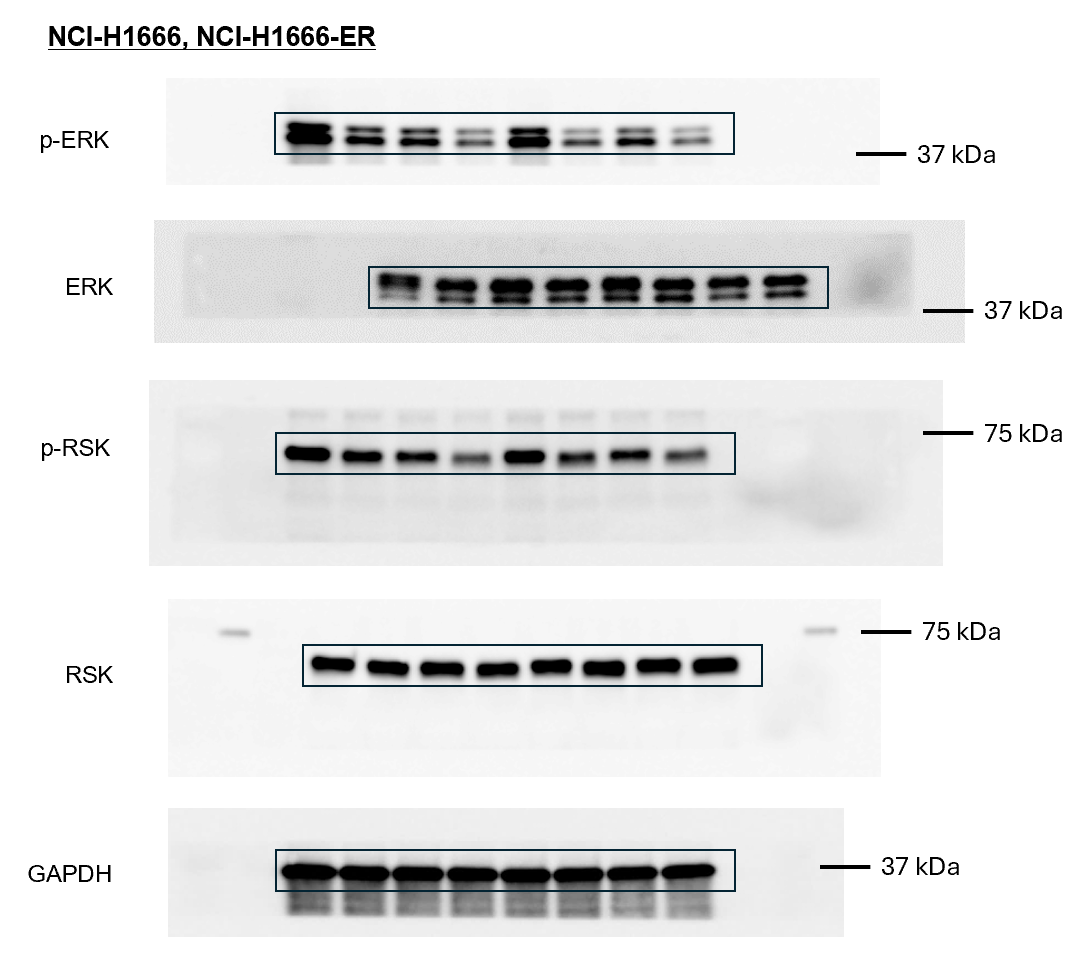


## Supplementary Figure 6b.


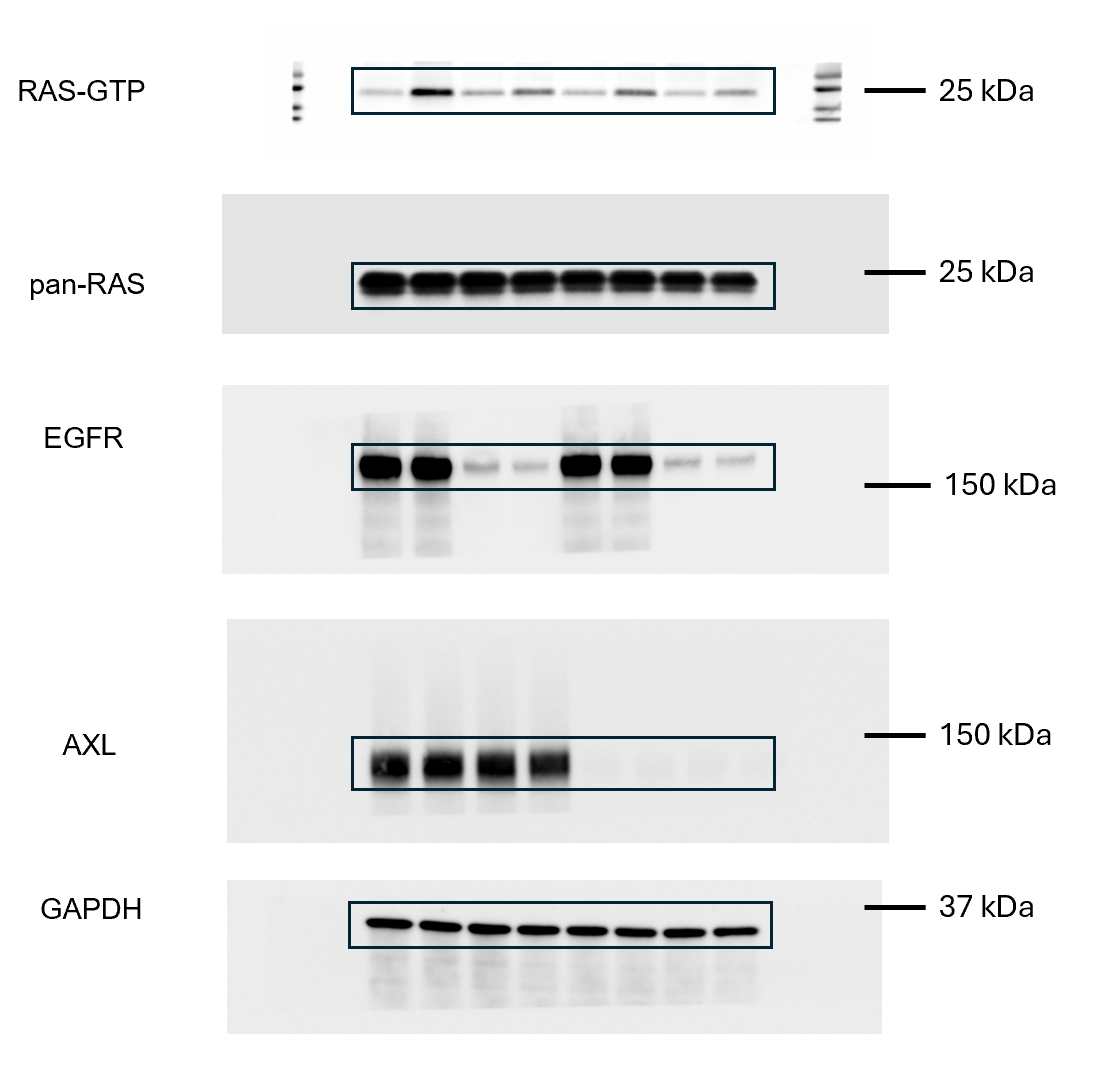


## Supplementary Figure 6d.


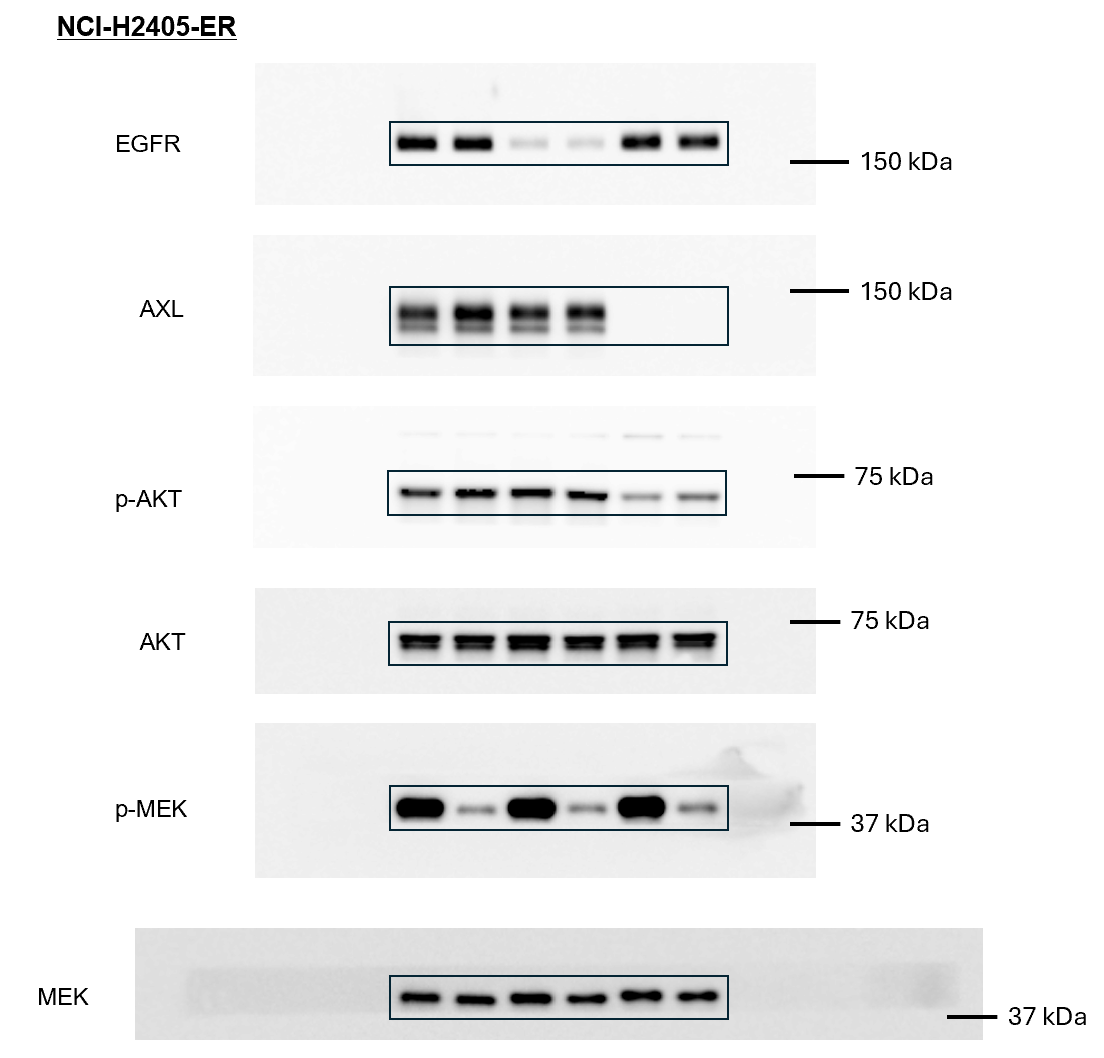

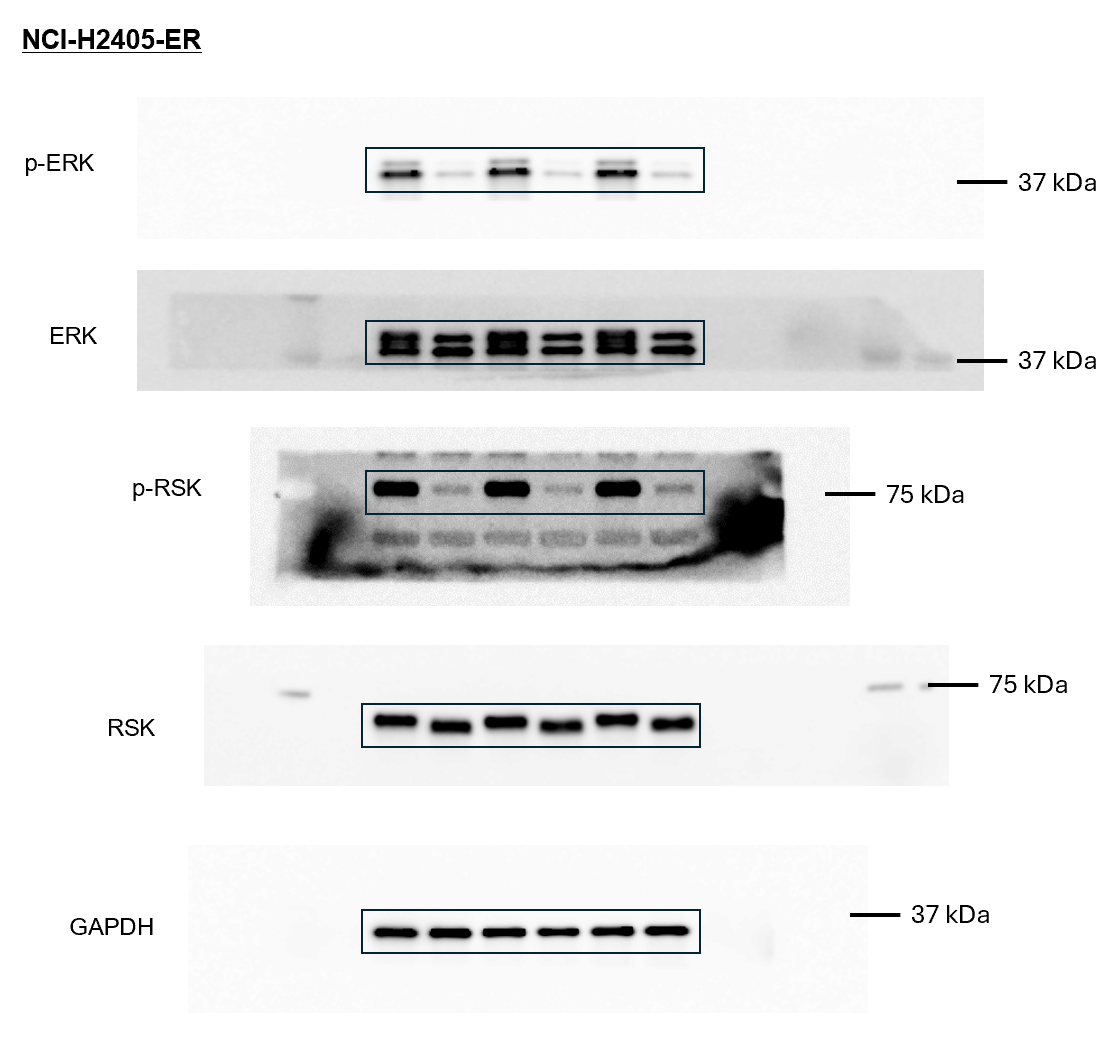


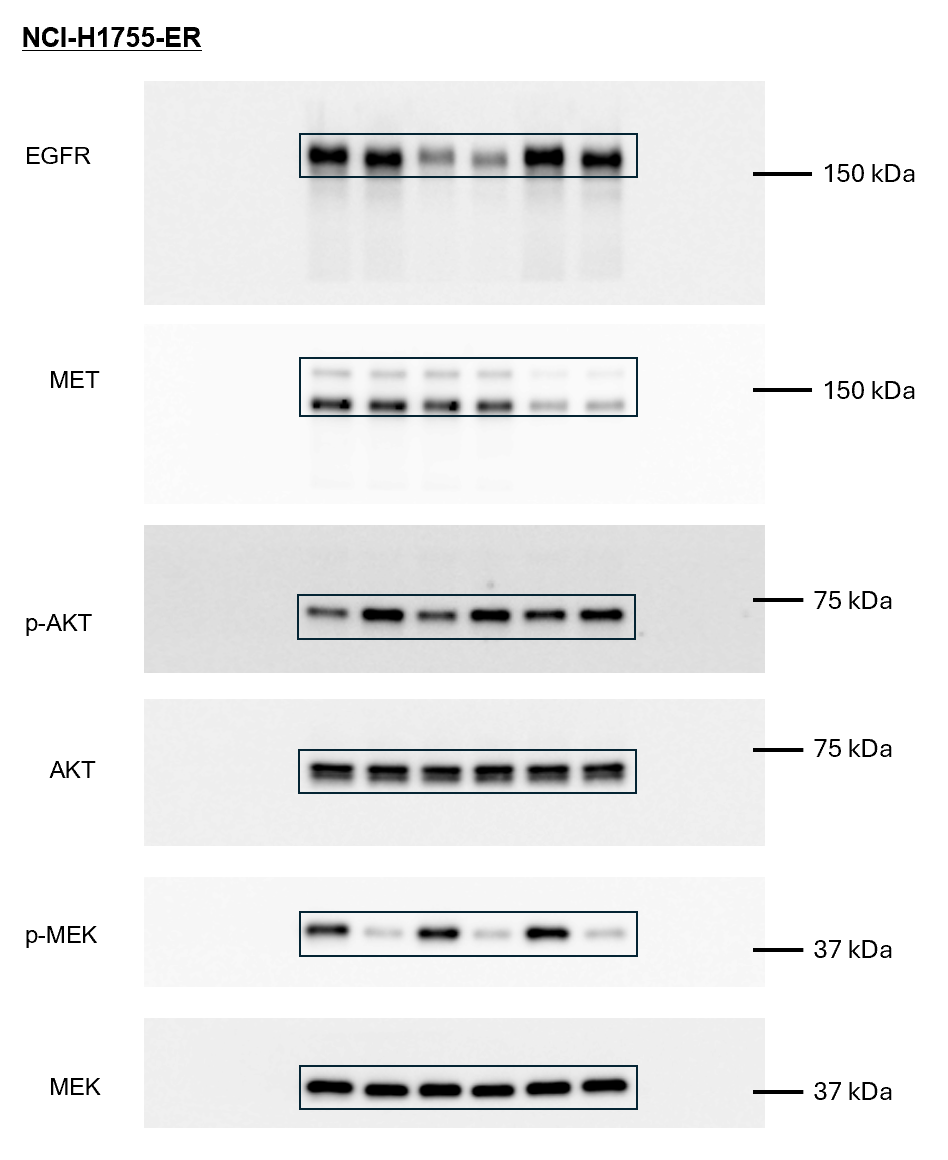

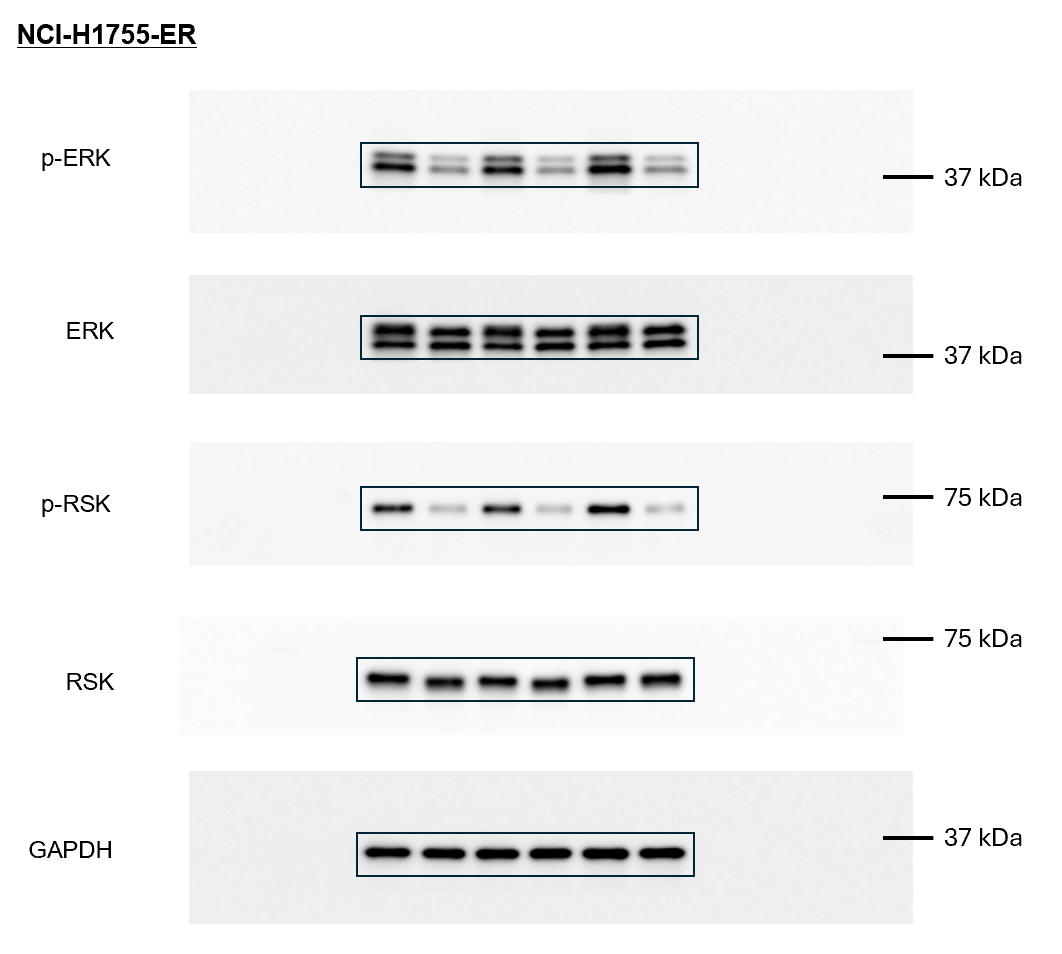


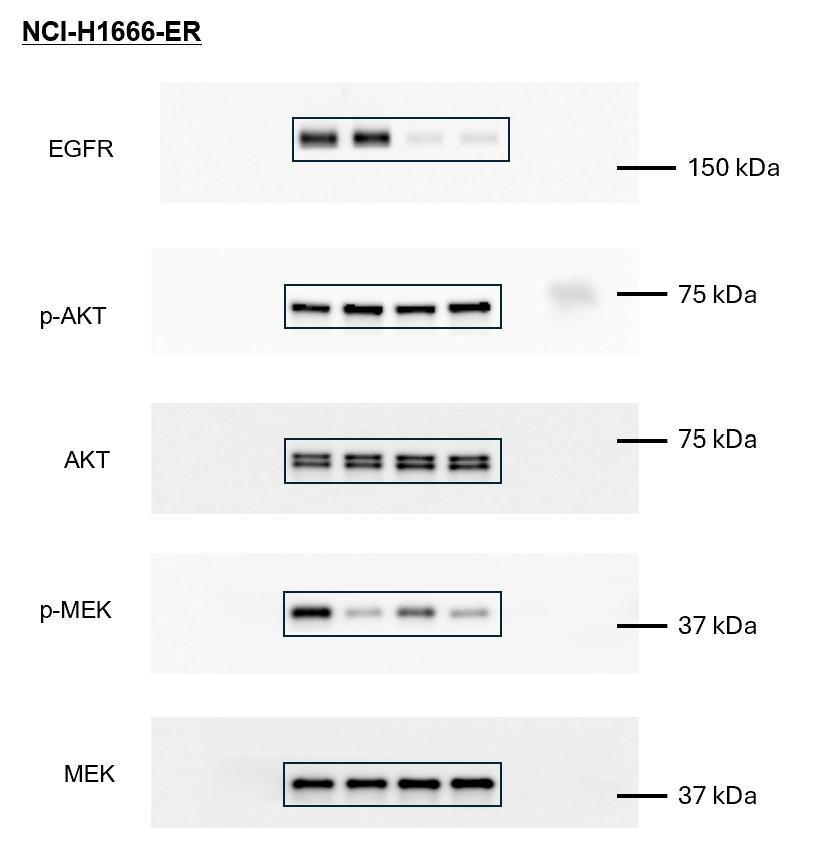

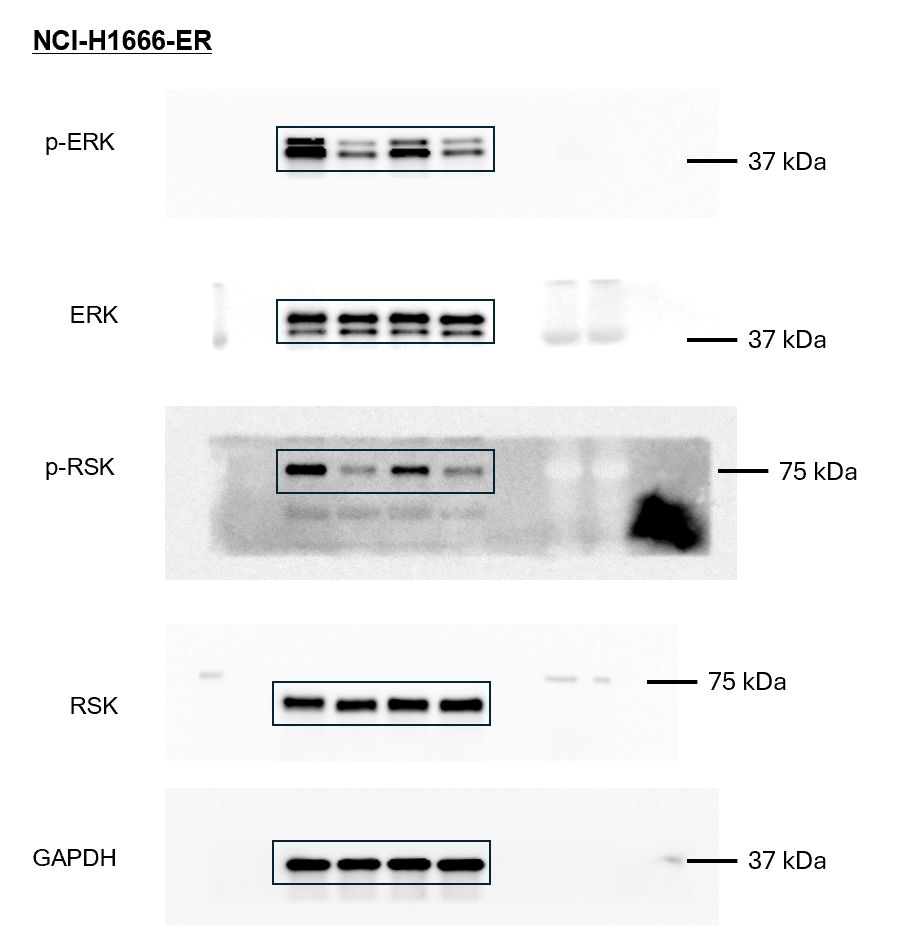


## Supplementary Figure 6e.


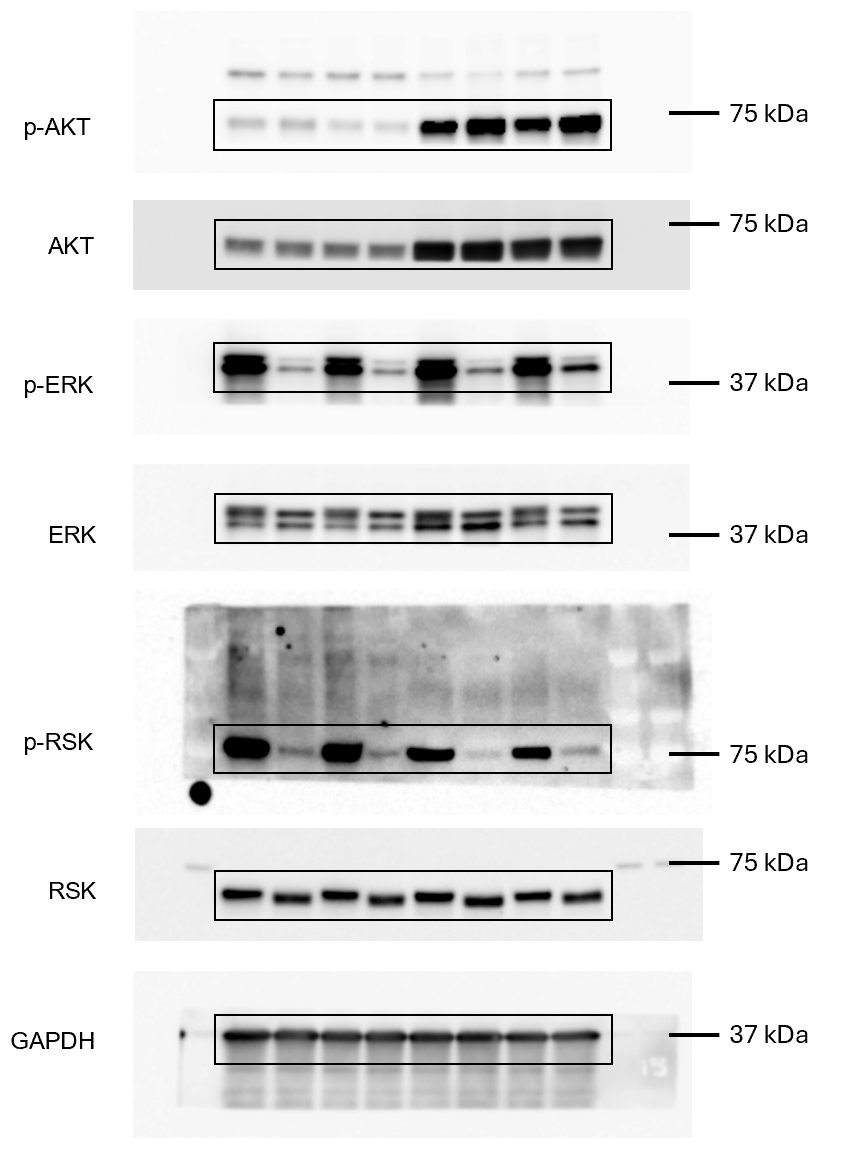


## Supplementary Figure 7b.


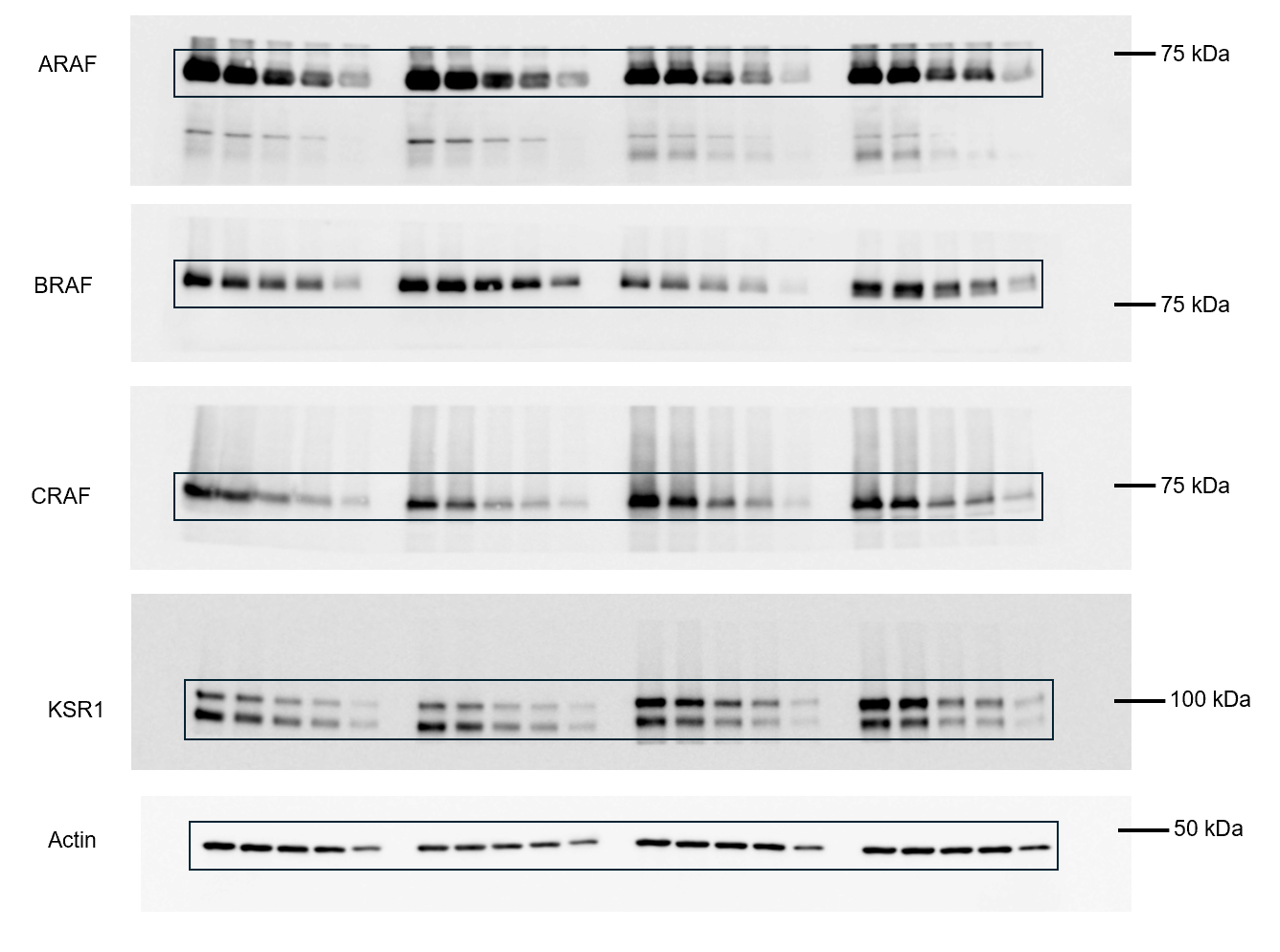


## Supplementary Figure 7c.


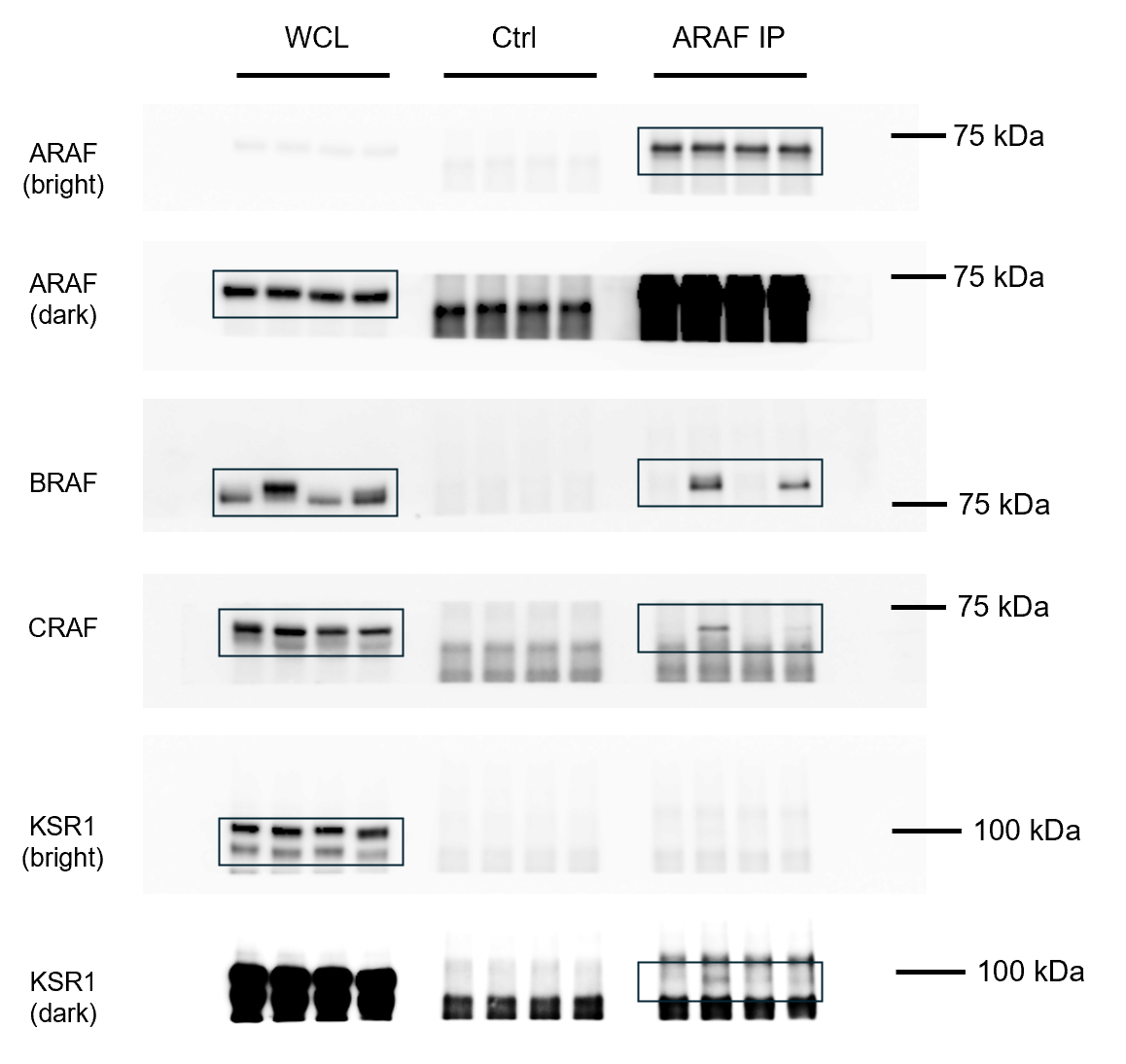


## Supplementary Figure 7d.


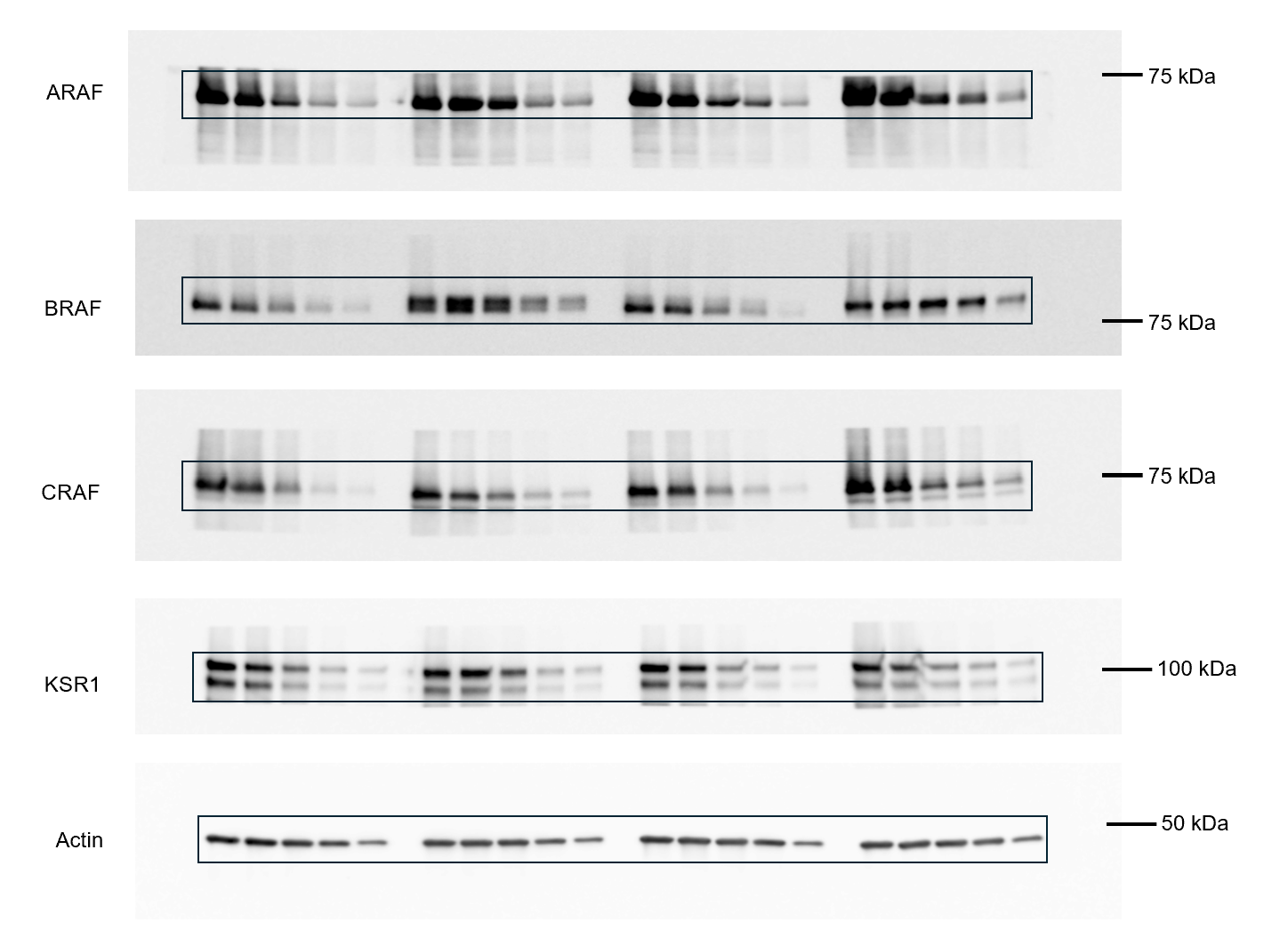


## Supplementary Figure 7g.


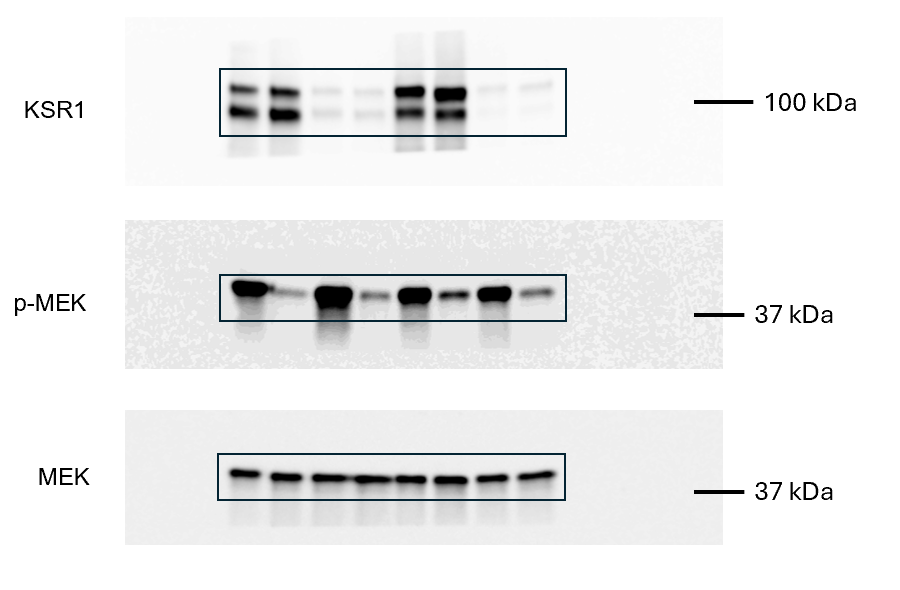

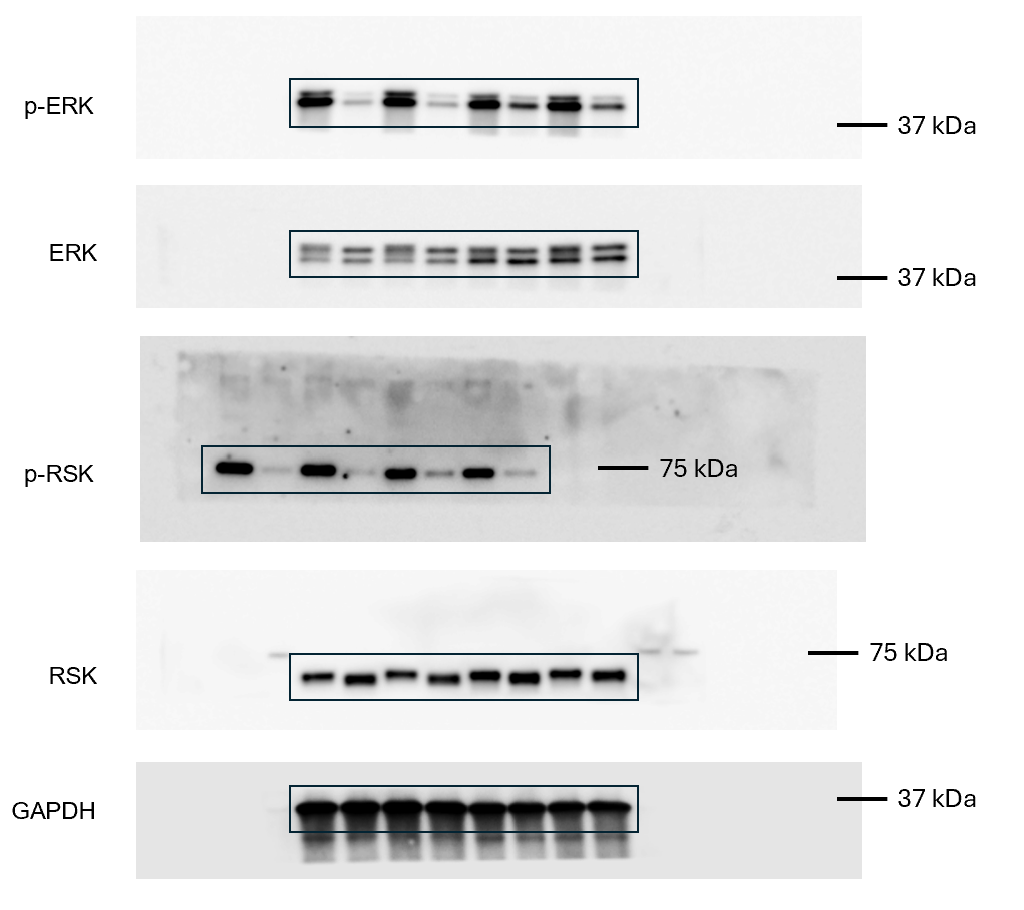


## Supplementary Figure 7h.


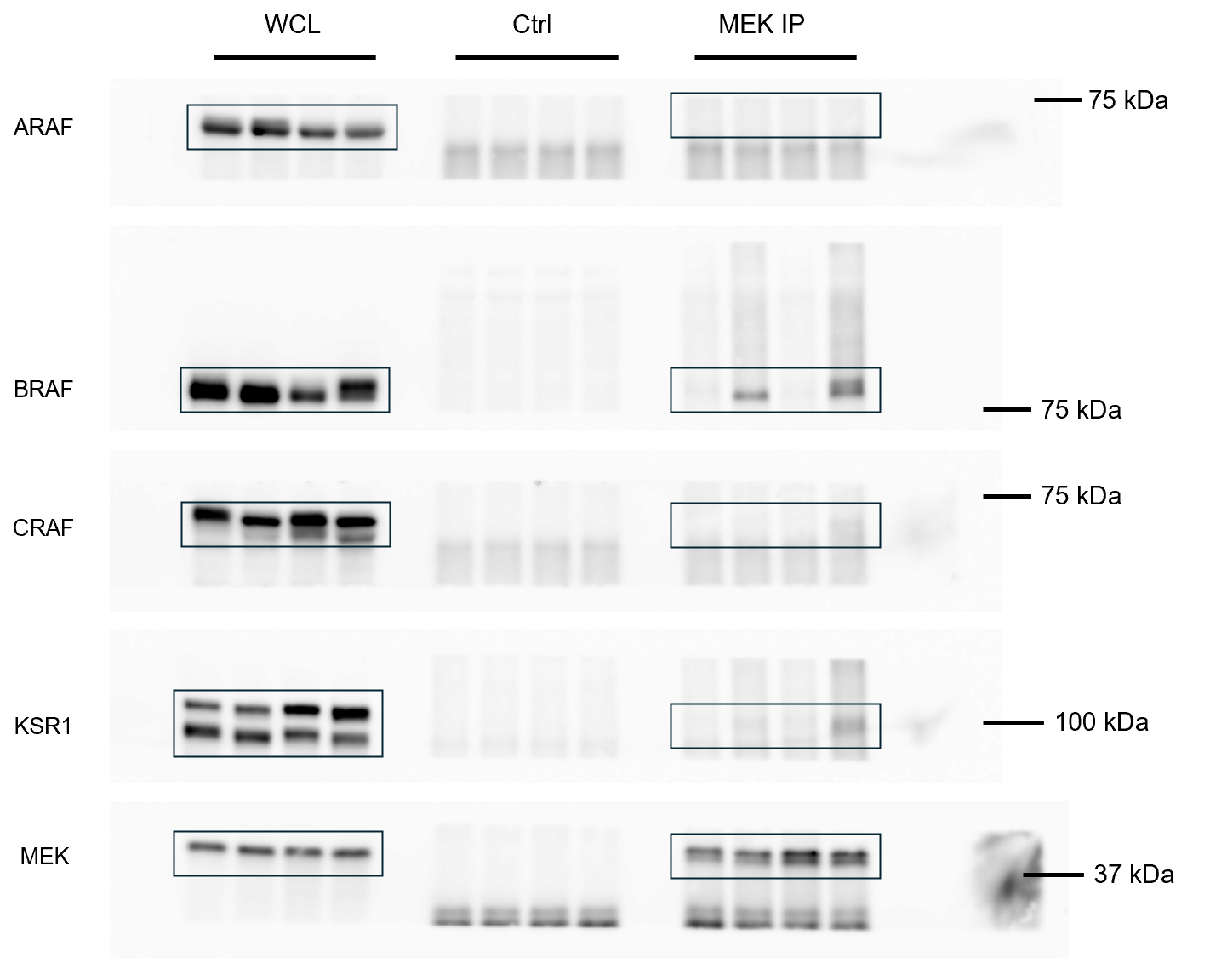


## Supplementary Figure 9a.


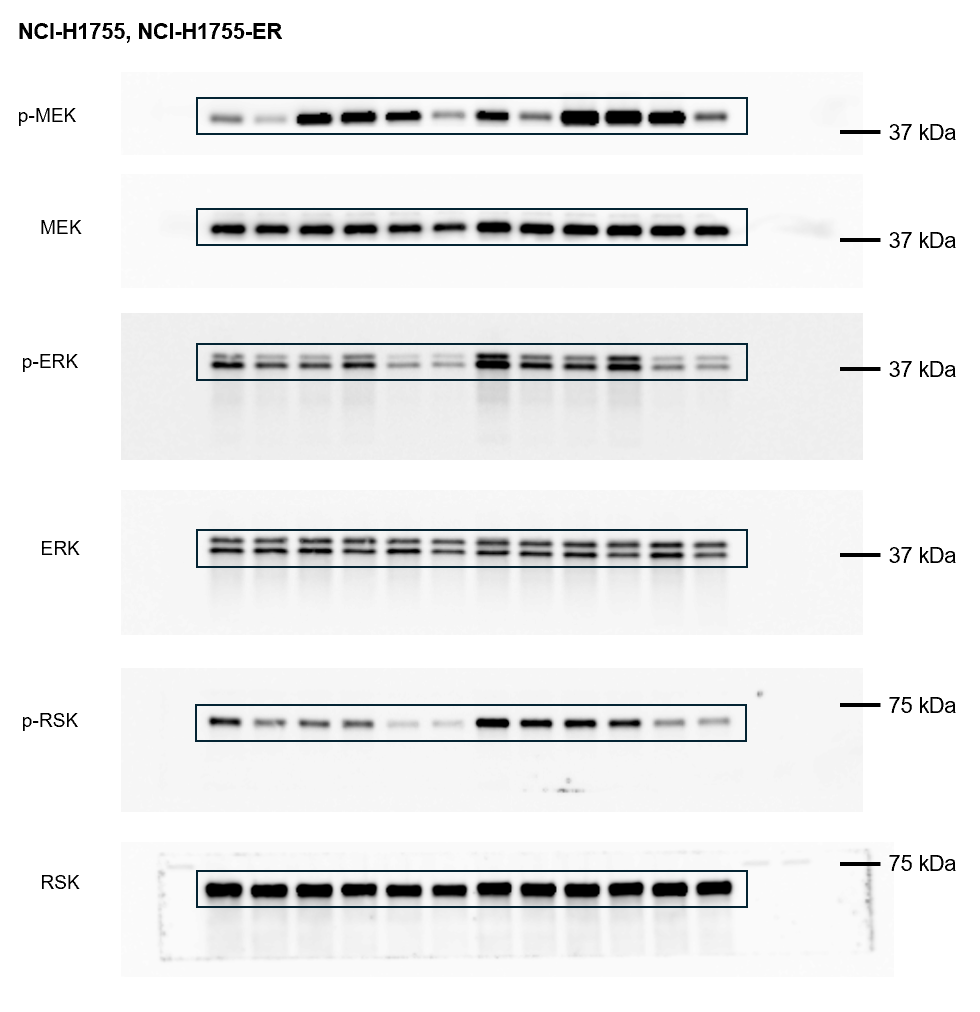

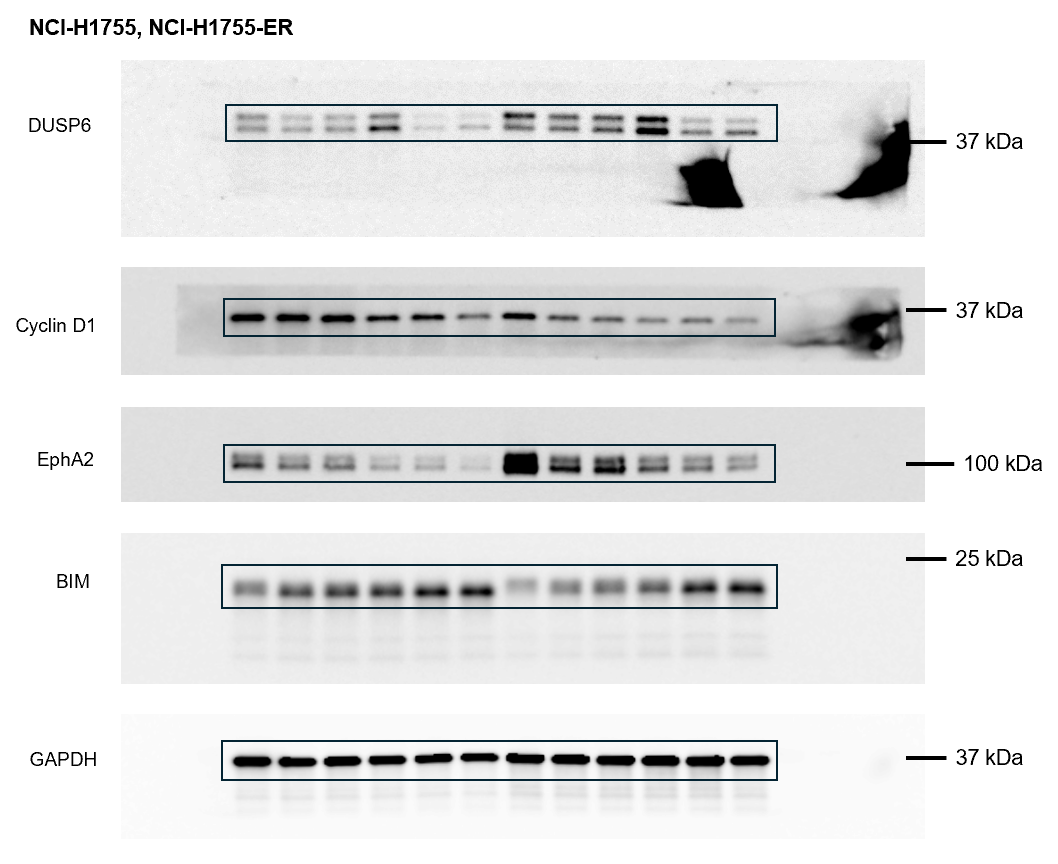


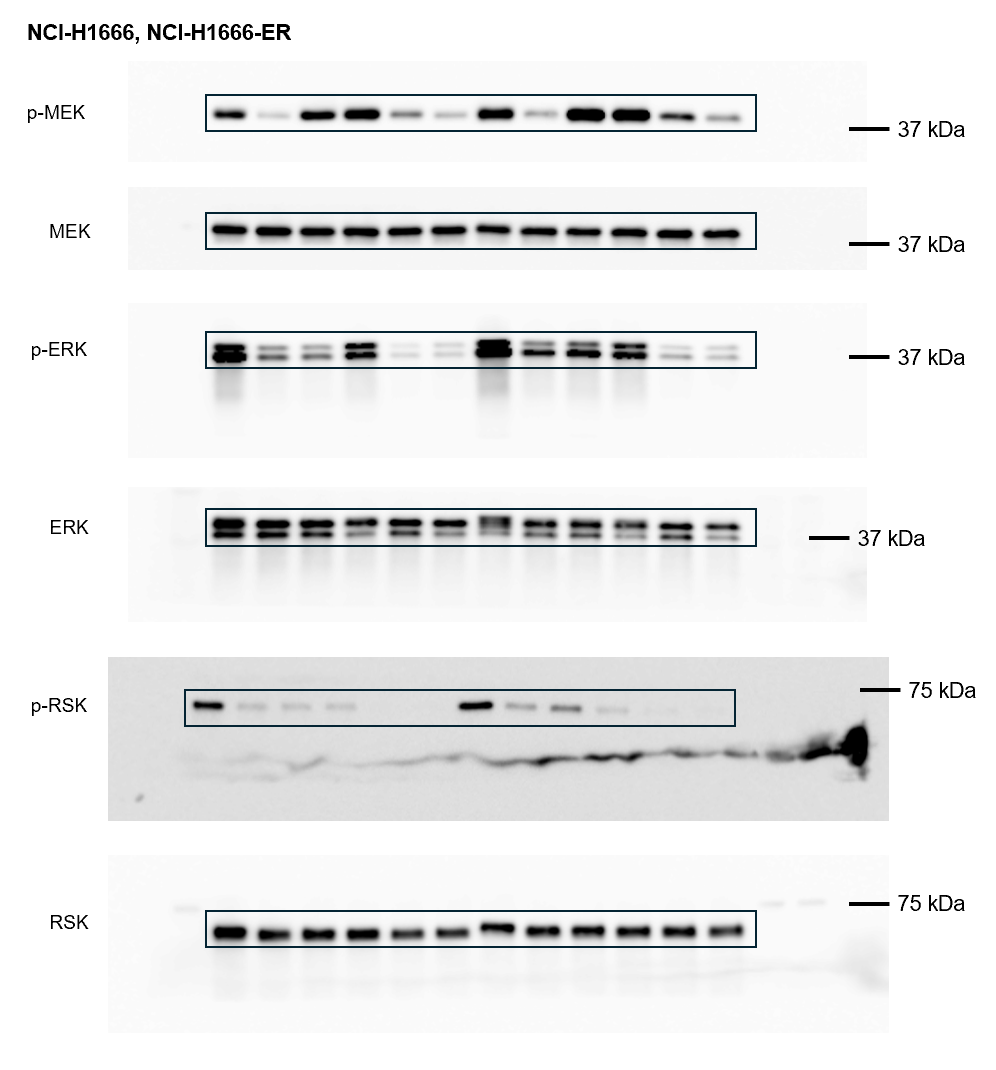

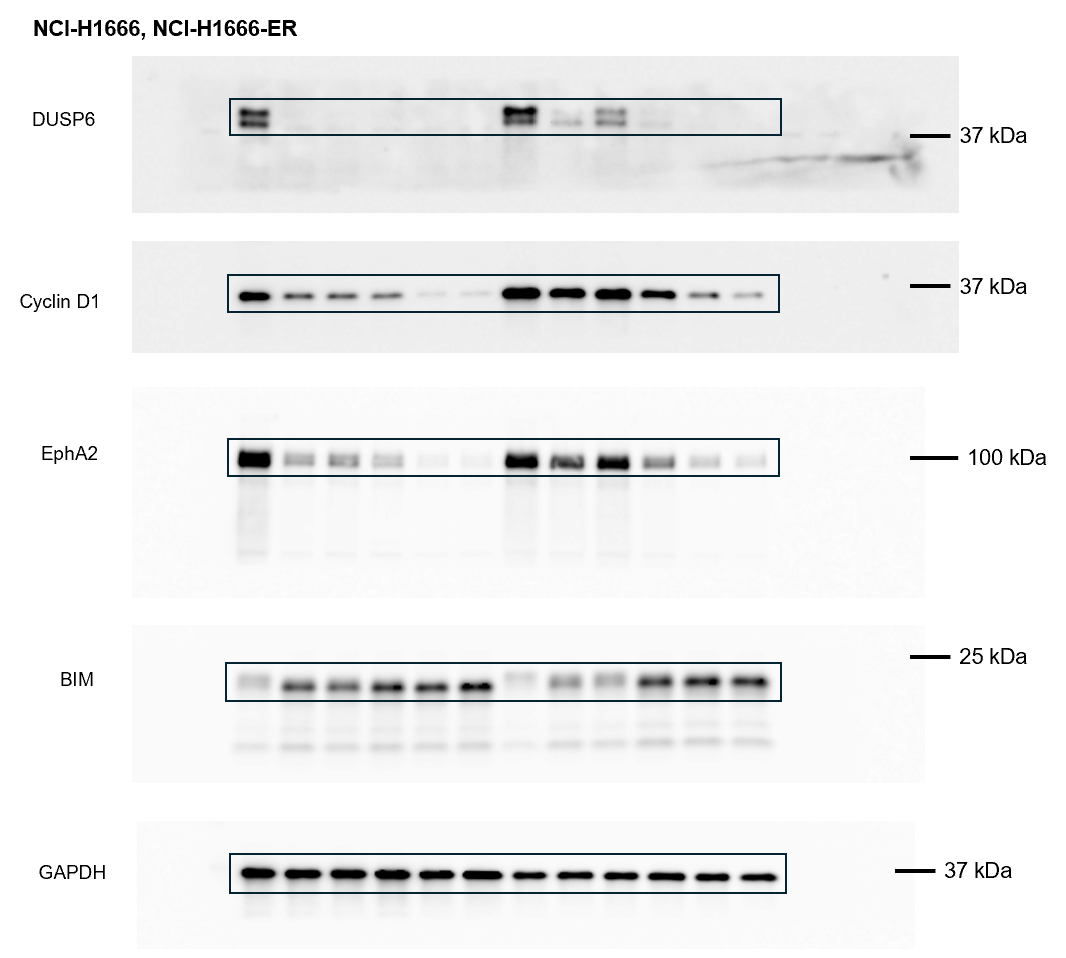


## Supplementary Figure 9b.


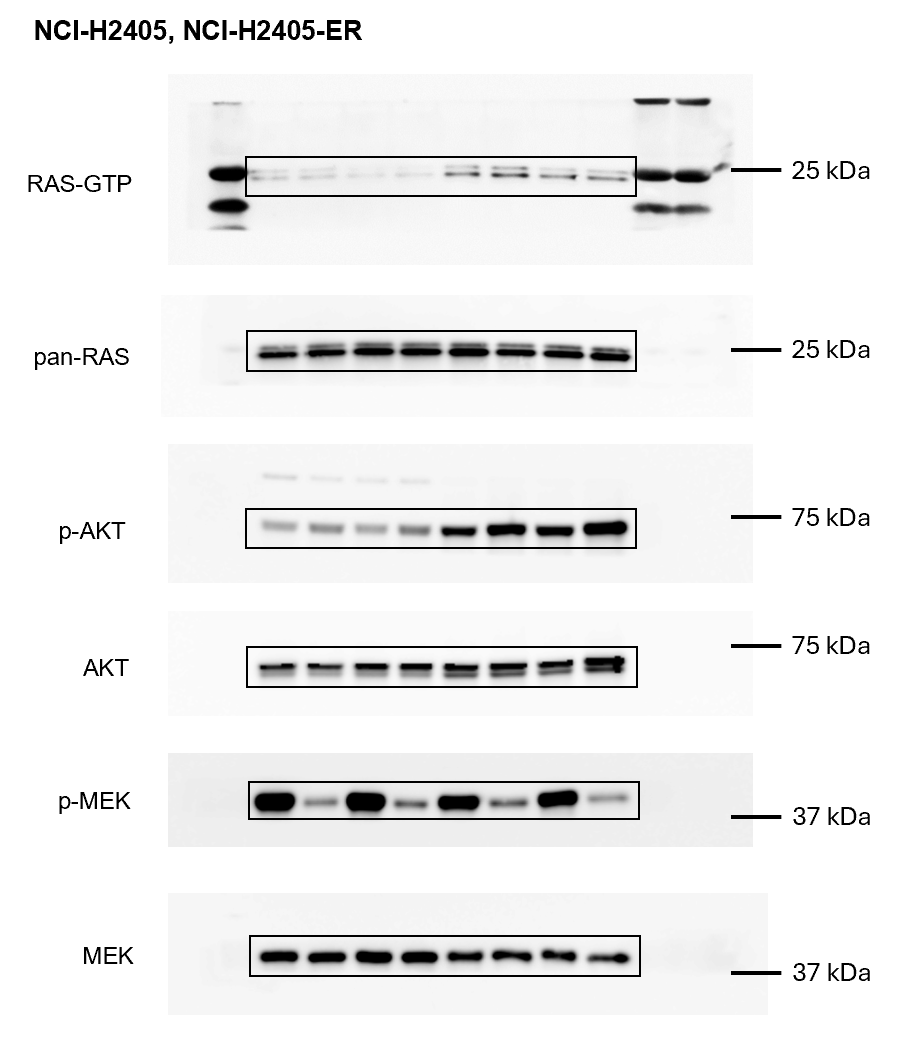

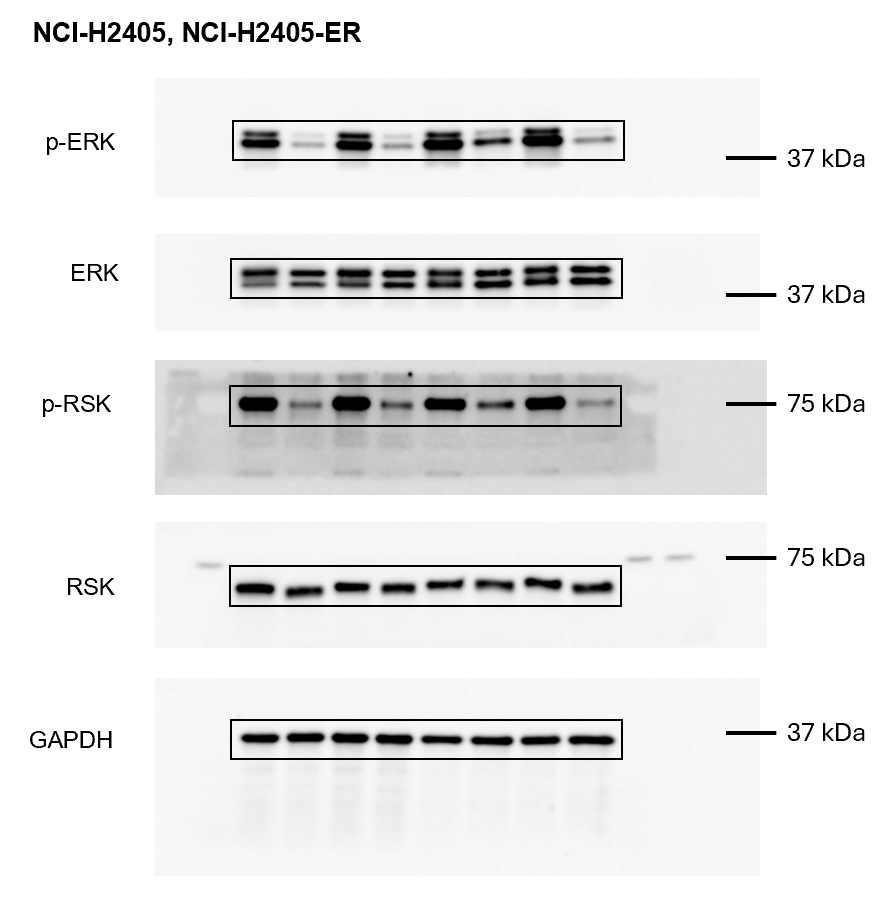


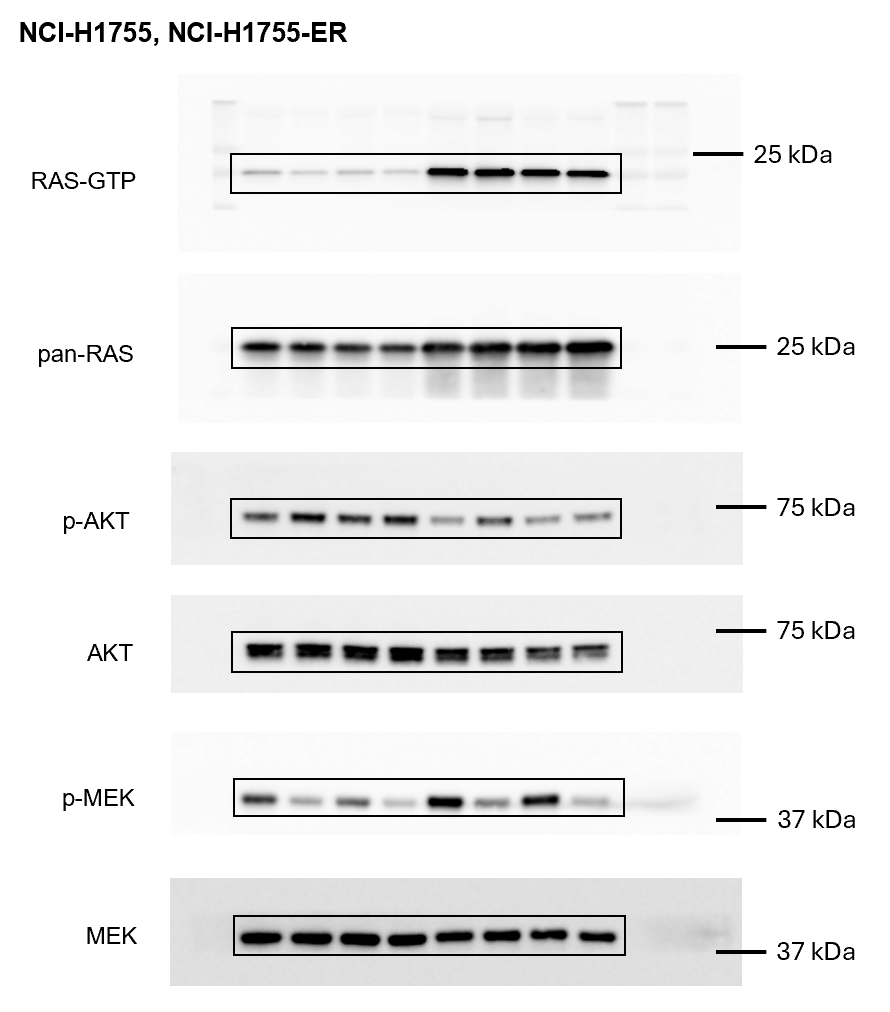

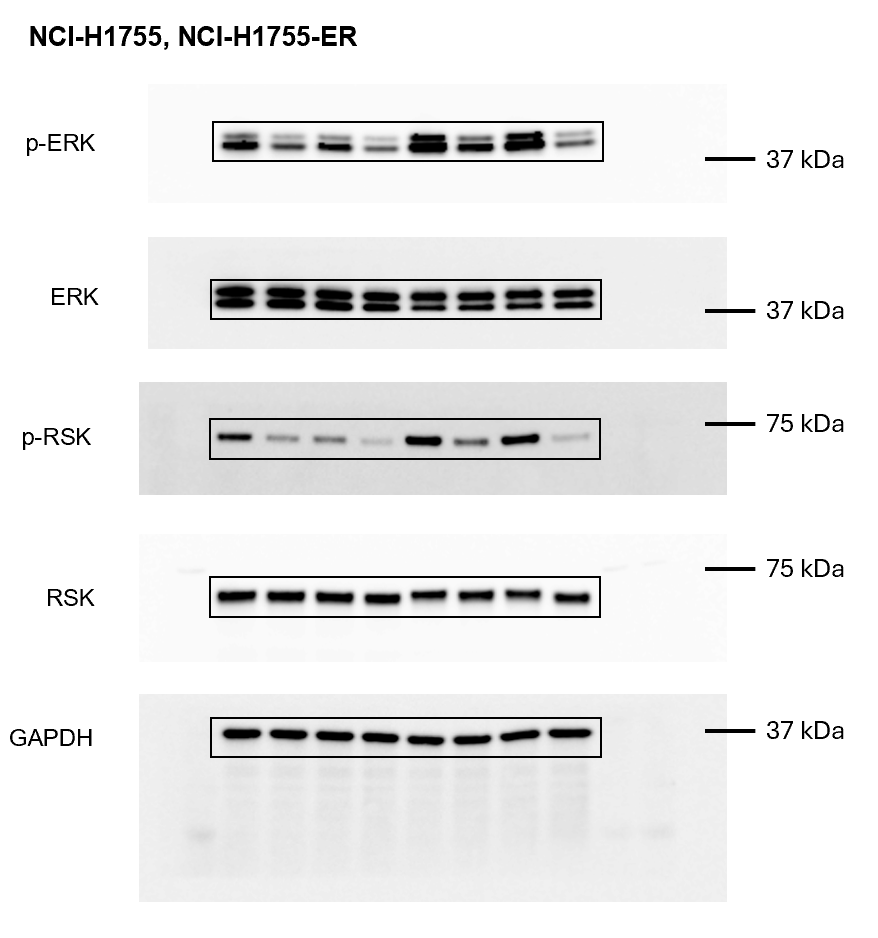


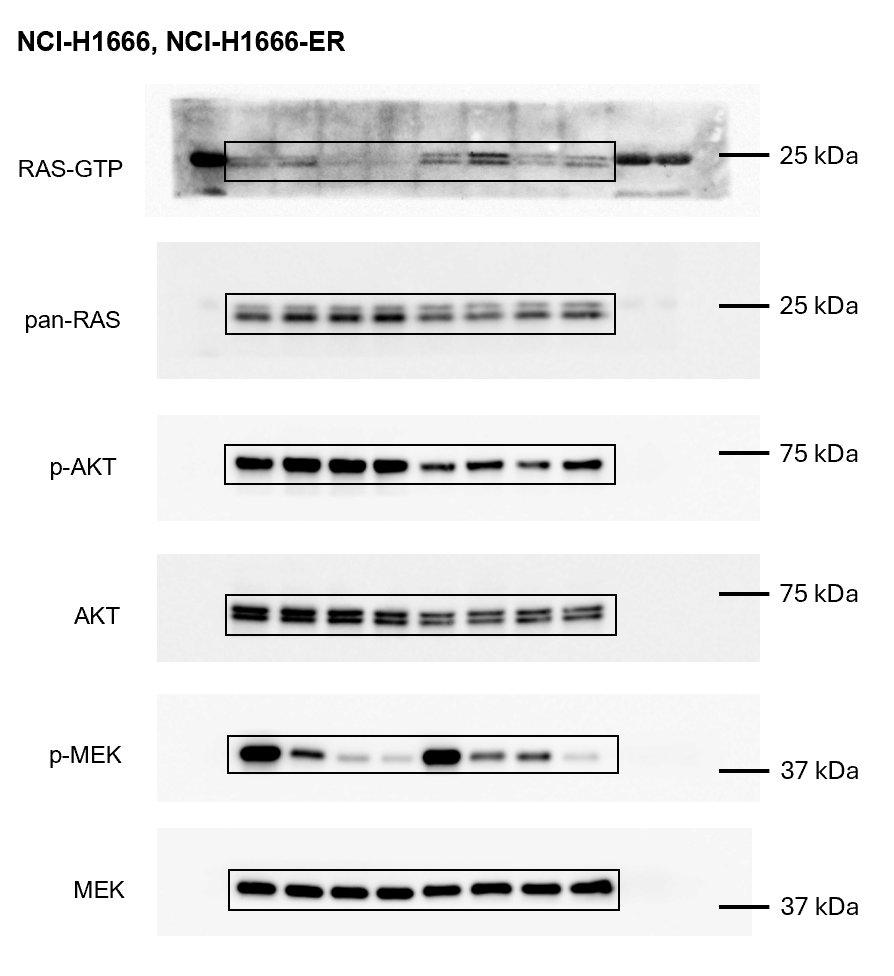

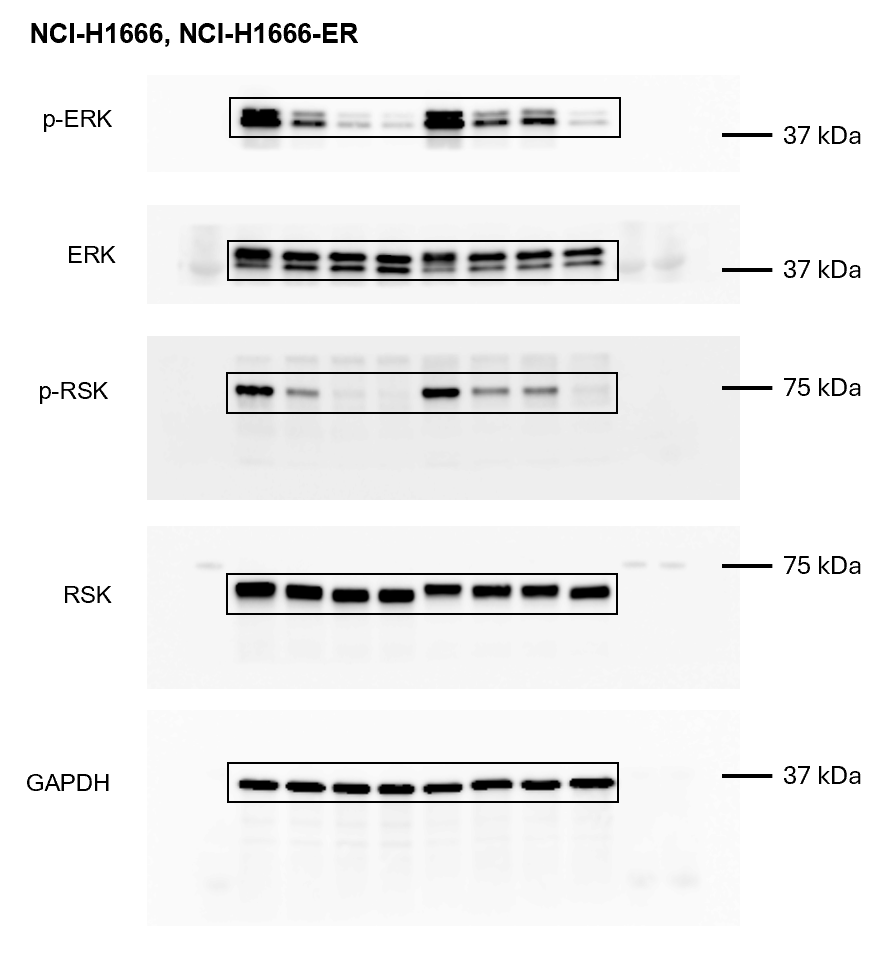


## Supplementary Figure 9c.


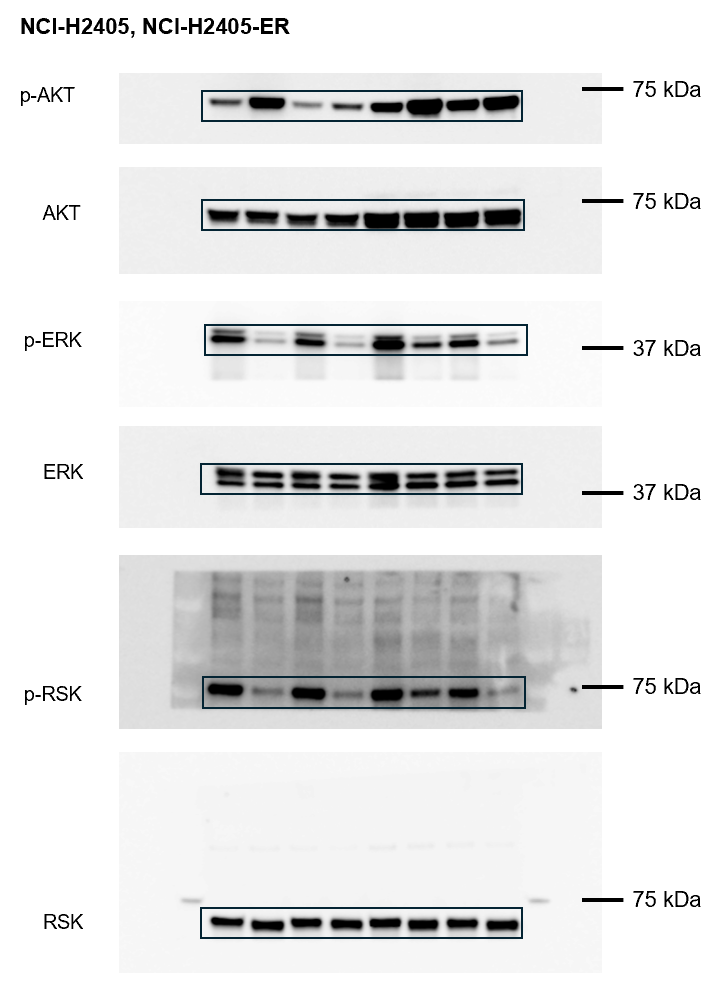

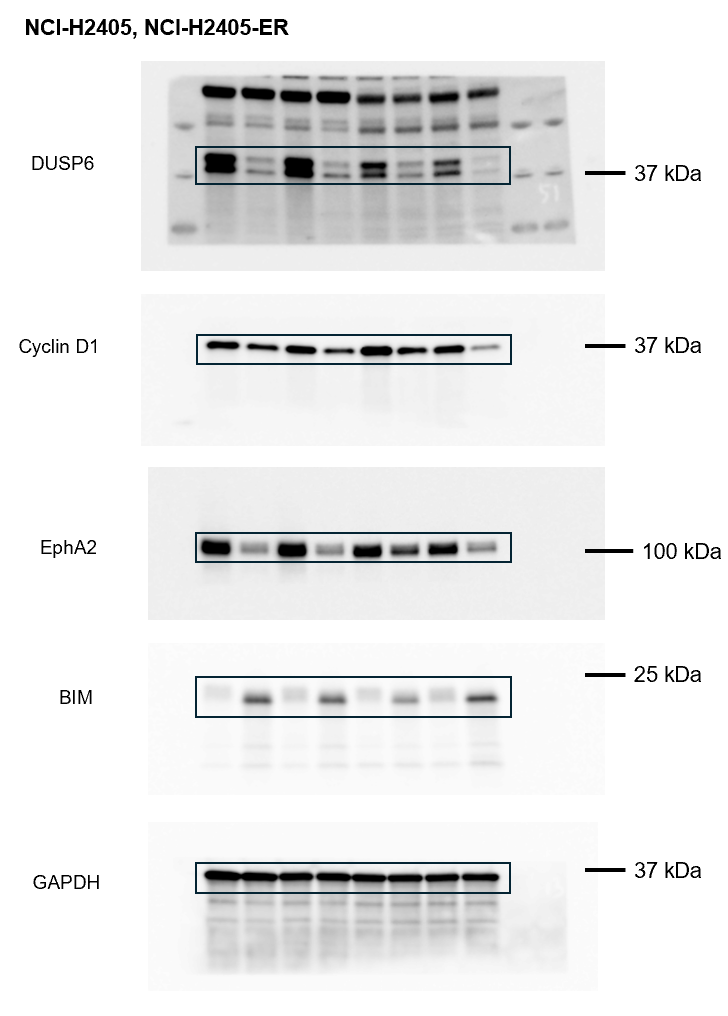


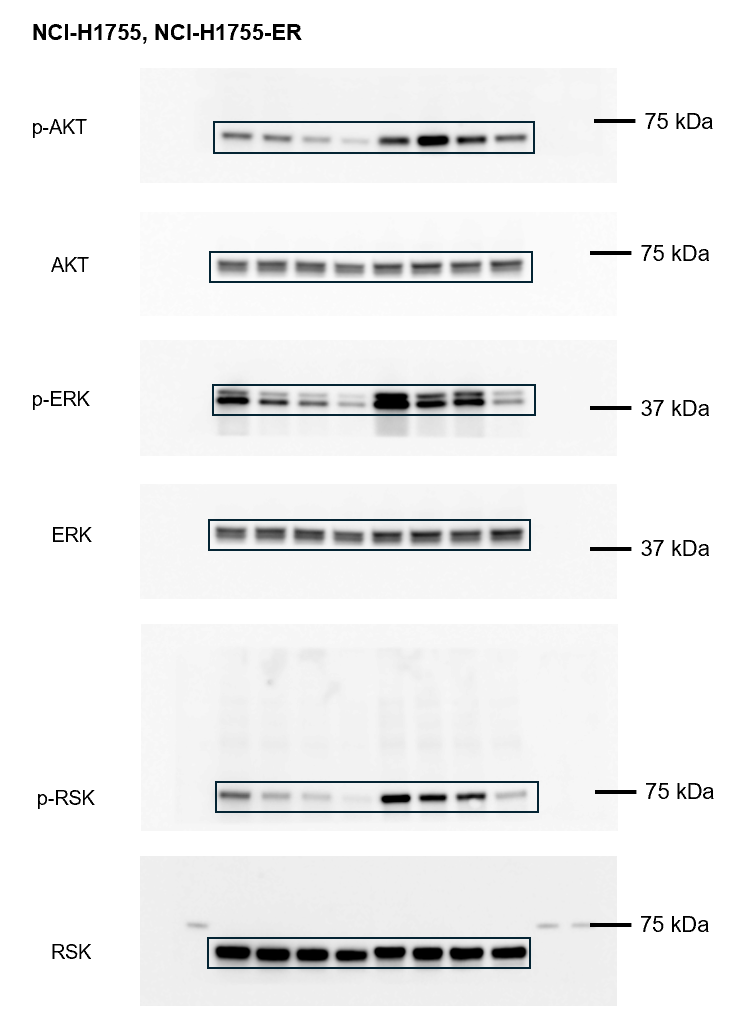

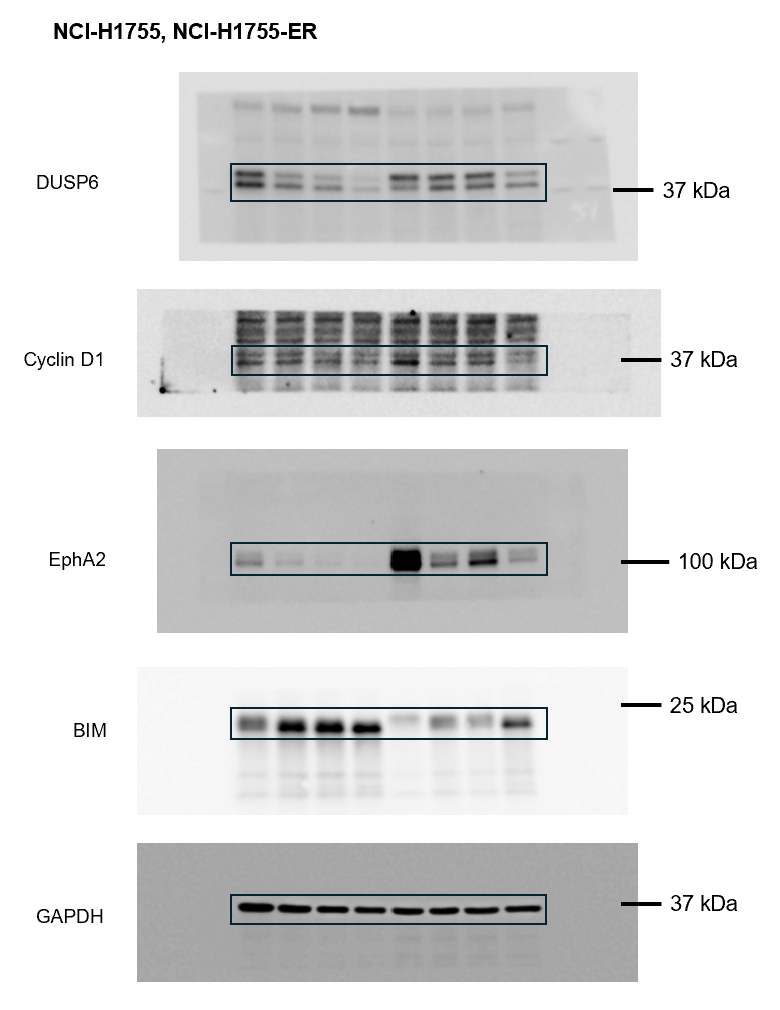


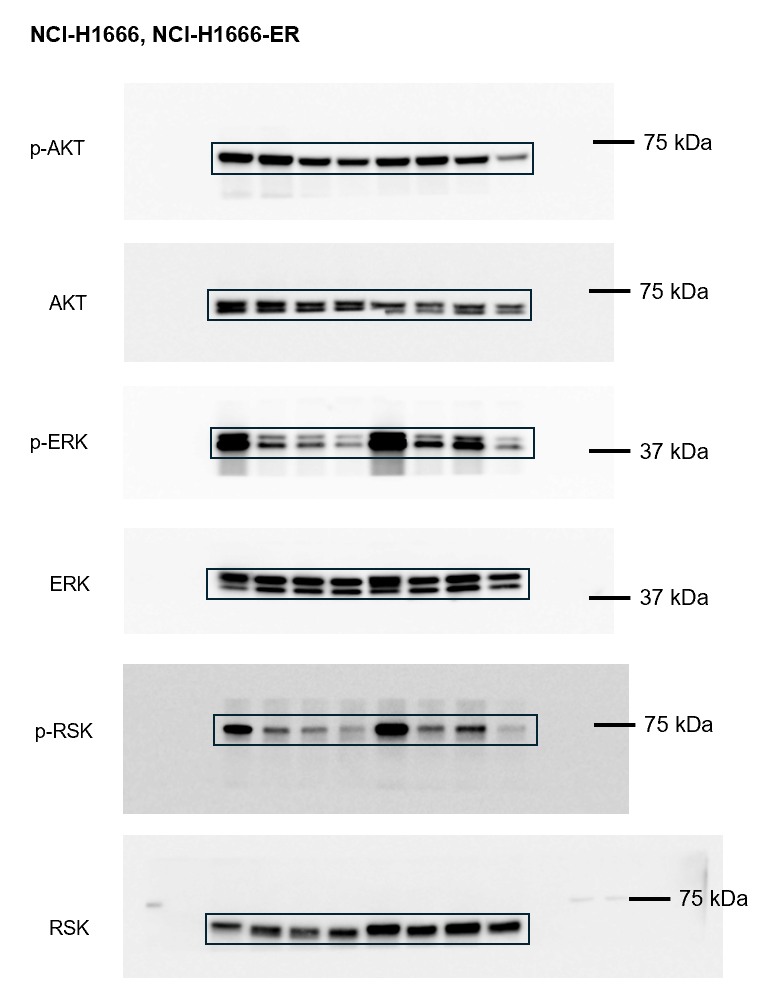

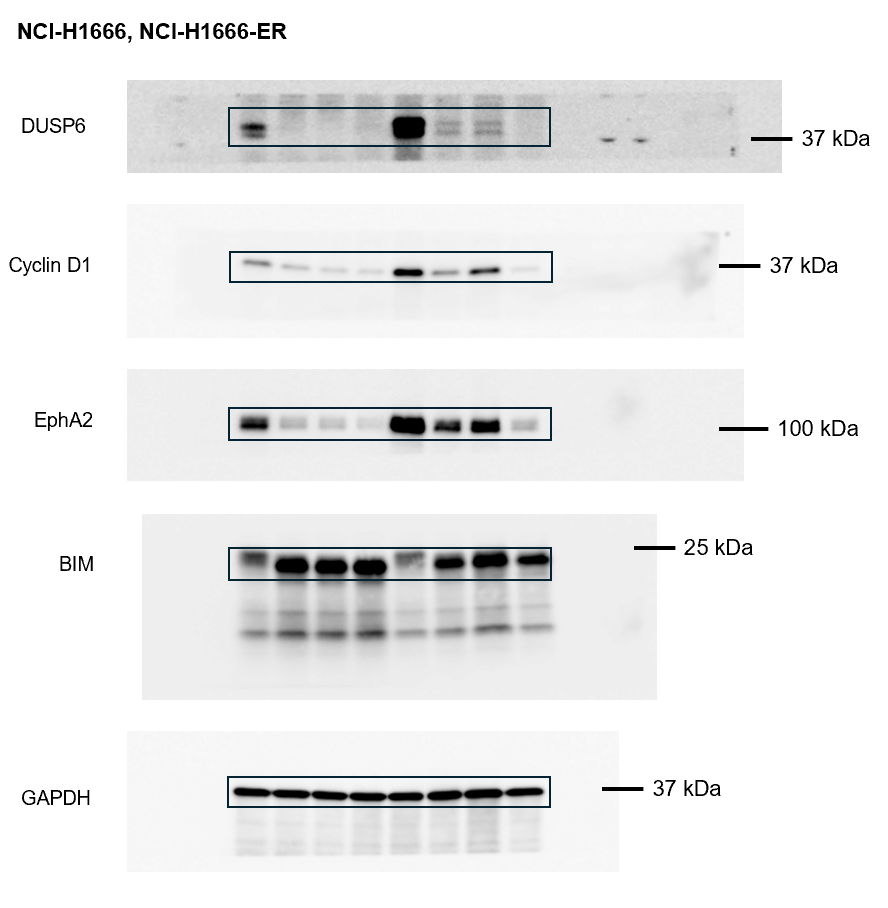


## Supplementary Figure 9d.


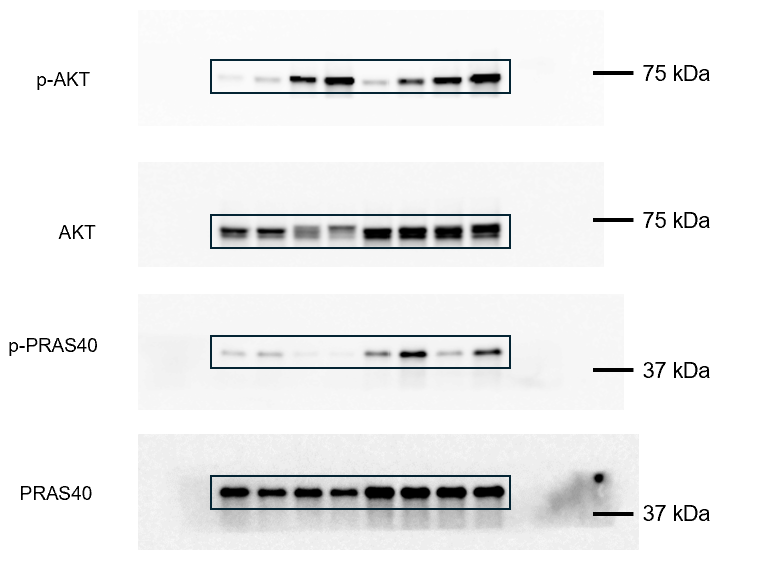

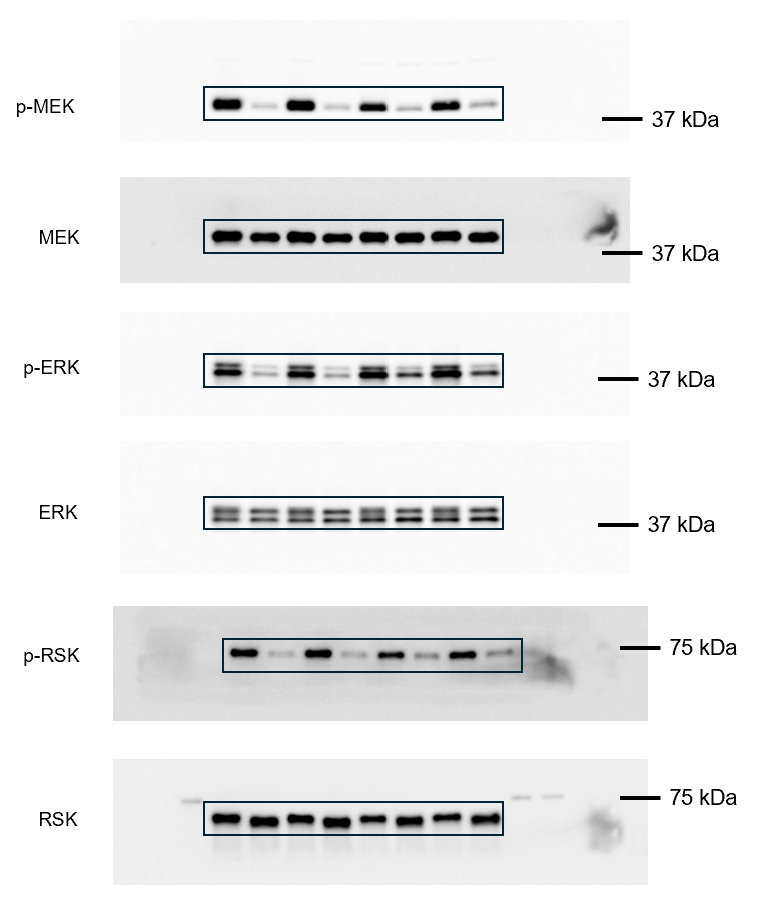


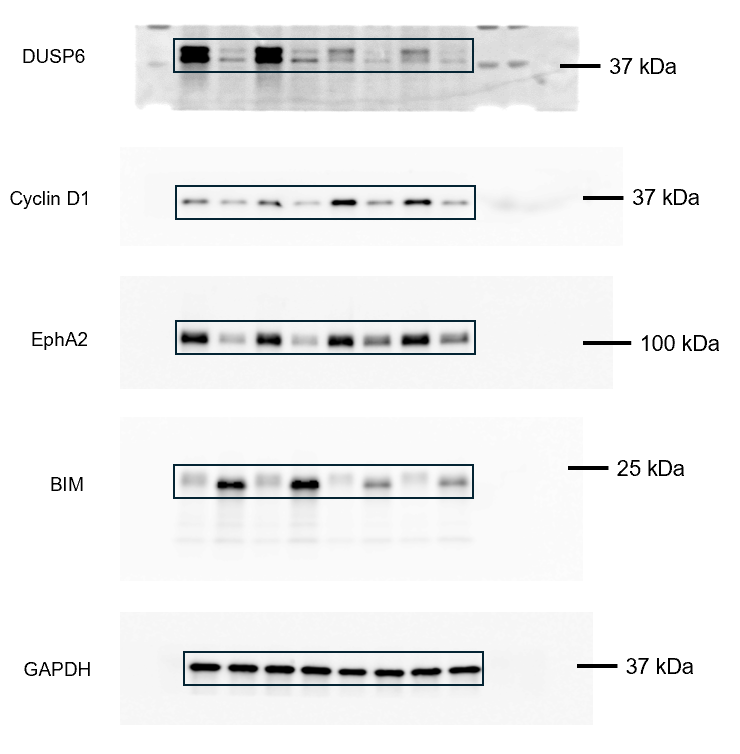


## Supplementary Figure 11b.


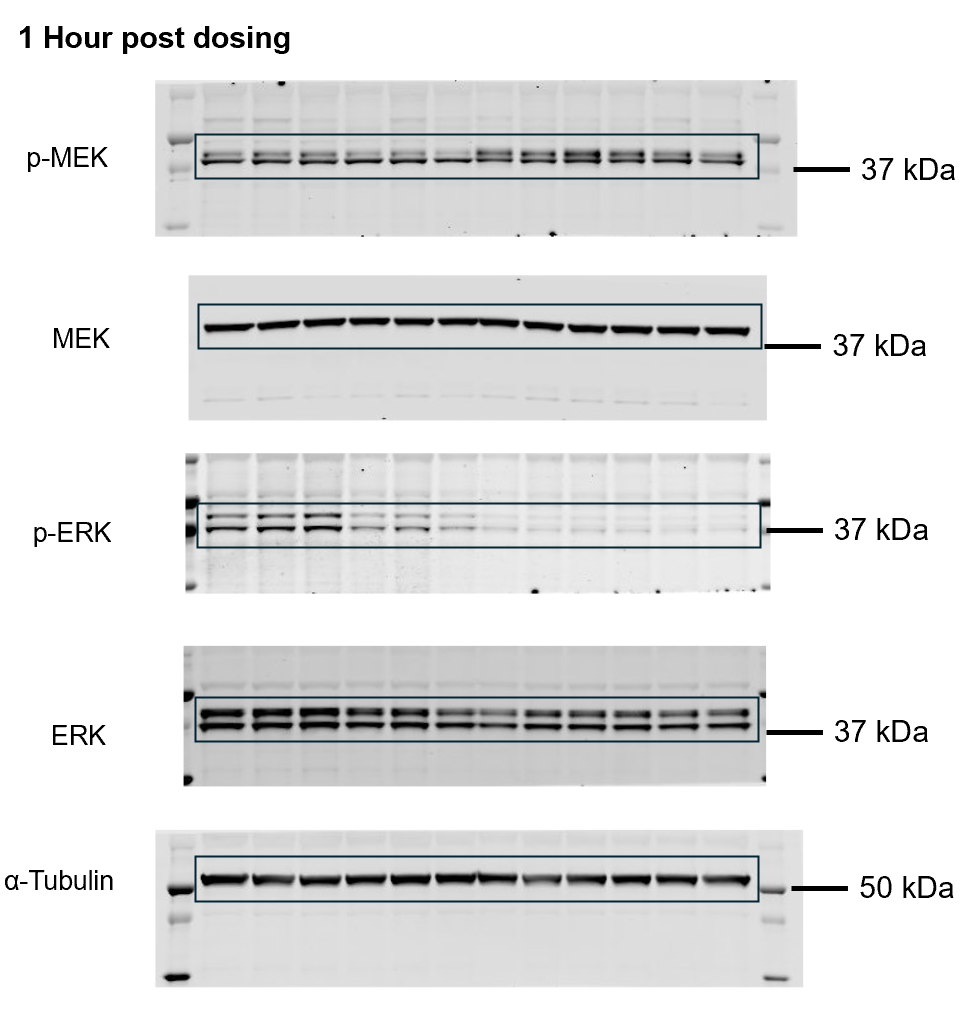


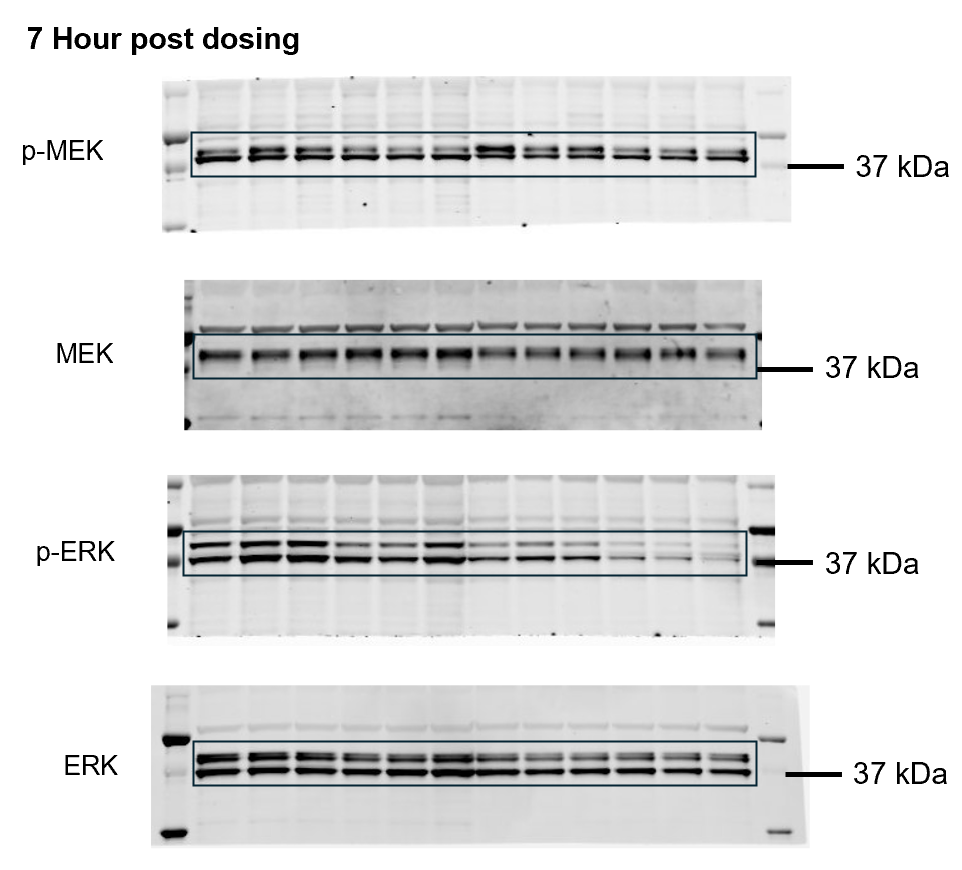

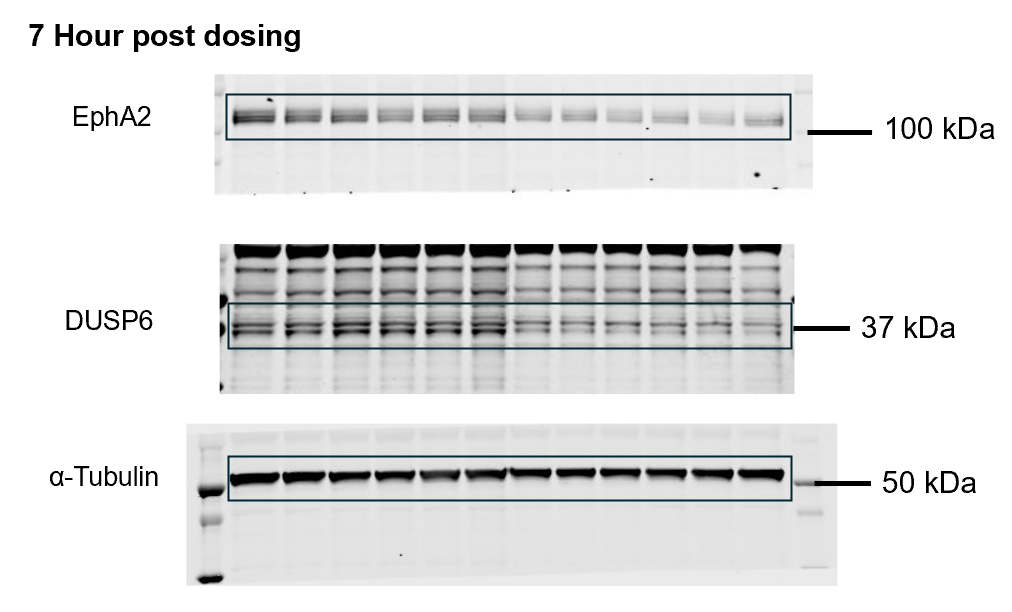


## Supplementary Figure 11c.


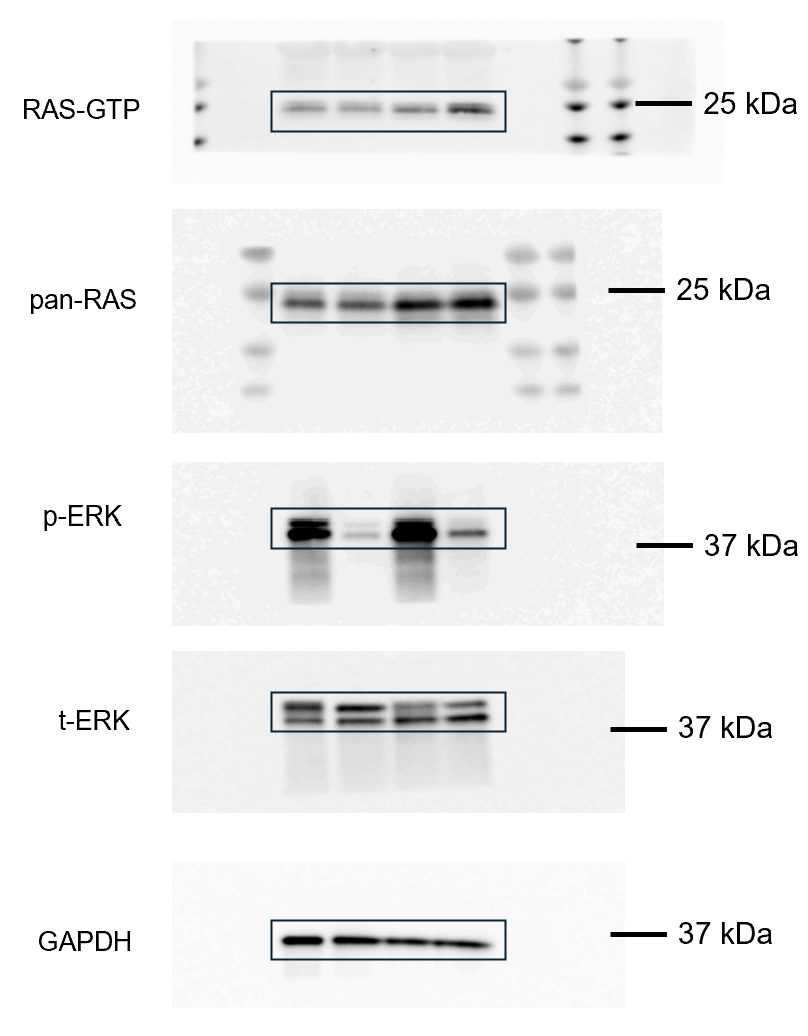


## Supplementary Figure 11d.


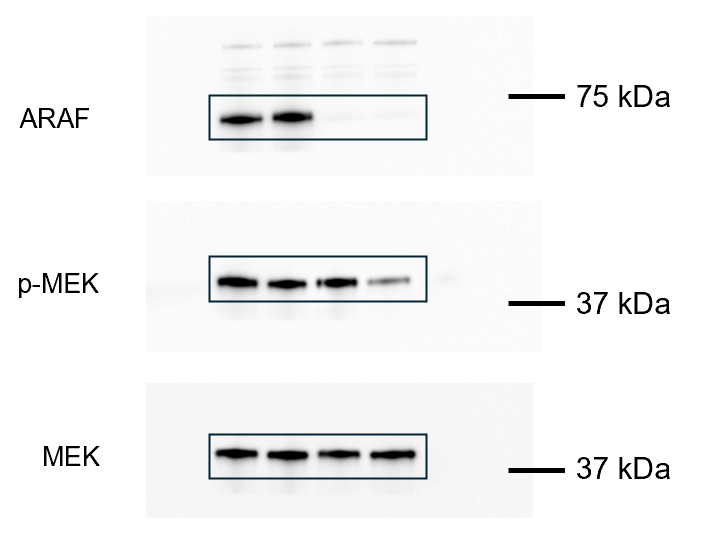

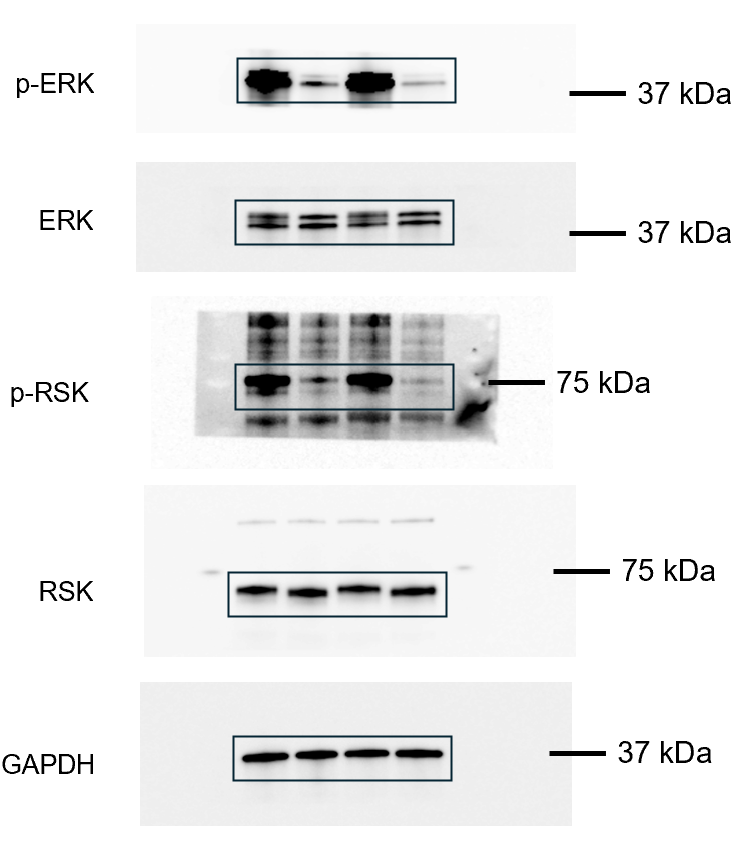

Supplement: Supplementary file 1 — Supplementary Information [file 41467_2026_69216_MOESM1_ESM.docx]
