# Supplementary material for: Pan-RAF inhibitor exarafenib targets BRAF class II/III NSCLC and reveals ARAF-KSR1 resistance and combination strategies
Source: Nat Commun. 2026 Feb 7;17:2484. doi: 10.1038/s41467-026-69216-3 (PMC12992618; doi:10.1038/s41467-026-69216-3)
Supplement: Supplementary file 2 — Description of Additional Supplementary Files [file 41467_2026_69216_MOESM2_ESM.pdf]

Title: Supplementary Data 1

Description: Treatment regimens in BRAF-mutant NSCLC patients. This file contains the complete list of first-, second-, and third-line therapies administered to patients with BRAF Class I, II, and III mutant NSCLC, as referenced in Figure 1d.

Title: Supplementary Data 2

Description: Specific concurrent RAS mutations by BRAF class. This file provides the detailed list and frequencies of specific KRAS, HRAS, and NRAS mutations identified in patients within each BRAF mutation class (Class I, II, and III), as summarized in Supplementary Figure 3e.
