# Supplementary material for: Pan-RAF inhibitor exarafenib targets BRAF class II/III NSCLC and reveals ARAF-KSR1 resistance and combination strategies
Source: Nat Commun. 2026 Feb 7;17:2484. doi: 10.1038/s41467-026-69216-3 (PMC12992618; doi:10.1038/s41467-026-69216-3)
Supplement: Supplementary file 5 — Reporting Summary [file 41467_2026_69216_MOESM5_ESM.pdf]

nature portfolio

Corresponding author(s): Tadashi Manabe, Trever Bivona

Last updated by author(s): Dec 28, 2025

Reporting Summary

Nature Portfolio wishes to improve the reproducibility of the work that we publish. This form provides structure for consistency and transparency in reporting. For further information on Nature Portfolio policies, see our Editorial Policies and the Editorial Policy Checklist.

Statistics

For all statistical analyses, confirm that the following items are present in the figure legend, table legend, main text, or Methods section.

n/a	Confirmed	
☐	☒	The exact sample size (*n*) for each experimental group/condition, given as a discrete number and unit of measurement
☐	☒	A statement on whether measurements were taken from distinct samples or whether the same sample was measured repeatedly
☐	☒	The statistical test(s) used AND whether they are one- or two-sided<br>*Only common tests should be described solely by name; describe more complex techniques in the Methods section.*
☐	☒	A description of all covariates tested
☐	☒	A description of any assumptions or corrections, such as tests of normality and adjustment for multiple comparisons
☐	☒	A full description of the statistical parameters including central tendency (e.g. means) or other basic estimates (e.g. regression coefficient) AND variation (e.g. standard deviation) or associated estimates of uncertainty (e.g. confidence intervals)
☐	☒	For null hypothesis testing, the test statistic (e.g. *F*, *t*, *r*) with confidence intervals, effect sizes, degrees of freedom and *P* value noted<br>*Give P values as exact values whenever suitable.*
☒	☐	For Bayesian analysis, information on the choice of priors and Markov chain Monte Carlo settings
☒	☐	For hierarchical and complex designs, identification of the appropriate level for tests and full reporting of outcomes
☒	☐	Estimates of effect sizes (e.g. Cohen's *d*, Pearson's *r*), indicating how they were calculated

Our web collection on statistics for biologists contains articles on many of the points above.

Software and code

Policy information about availability of computer code

Data collection	SoftMax Pro (SpectraMax M5 plate reader), ImageQuant (GE Healthcare LAS 4000)
Data analysis	GraphPad Prism 7, Adobe Illustrator (28.6), R (version 3.3.1), Fiji Image J, Microsoft Excel., SAS 9.4., Combenefit, PyMOL, AlphaFold3.

For manuscripts utilizing custom algorithms or software that are central to the research but not yet described in published literature, software must be made available to editors and reviewers. We strongly encourage code deposition in a community repository (e.g. GitHub). See the Nature Portfolio guidelines for submitting code & software for further information.

Data

Policy information about availability of data

All manuscripts must include a data availability statement. This statement should provide the following information, where applicable:

- Accession codes, unique identifiers, or web links for publicly available datasets
- A description of any restrictions on data availability
- For clinical datasets or third party data, please ensure that the statement adheres to our policy

The Whole Genome Sequencing (WGS) data generated in this study are publicly available as an NCBI Bioproject under accession number PRJNA1123823 (https://www.ncbi.nlm.nih.gov/bioproject/PRJNA1123823). The datasets generated and analyzed during the current study using the GuardantINFORM™ database are not publicly available due to the use of a third-party healthcare claims database with associated privacy and contractual restrictions. Access to the data is subject to approval by Guardant Health, Inc. Researchers interested in accessing the data for verification purposes may submit requests to Guardant Health directly. The

remaining data are available within the Article, Supplementary Information, or Source Data file. Biological material (e.g. cell lines) generated in this study is available by request from the corresponding author. Additional reagents can be made available upon reasonable request. Source data are provided as a Source Data file.

Research involving human participants, their data, or biological material

Policy information about studies with human participants or human data. See also policy information about sex, gender (identity/presentation), and sexual orientation and race, ethnicity and racism.

Reporting on sex and gender	Sex was considered as a biological variable in the study design to assess potential associations with BRAF mutation classes within the GuardantINFORM™ database analysis. Sex was determined based on the information recorded in the linked healthcare claims database. Our analysis identified a statistically significant difference in distribution, with Class I mutations being more frequently observed in females compared to males, while Class II and III mutations were more prevalent in patients with a history of tobacco use, as detailed in the Results section and Supplementary Table 6. For the clinical case studies (n=2) from the KN-8701 trial, sex is not reported to protect patient anonymity.
Reporting on race, ethnicity, or other socially relevant groupings	Race, ethnicity, or other socially relevant groupings were not collected or analyzed in this study.
Population characteristics	The genomic analysis included 2,398 patients with advanced/metastatic NSCLC harboring BRAF Class I, II, or III mutations identified in the GuardantINFORM™ database. The median age was 70 years. Covariates analyzed included age, sex, and tobacco use history. For the clinical case studies (n=2), patients were diagnosed with Stage 4 lung adenocarcinoma and had progressed on prior standard therapies. Specific demographic details (age and sex) are not reported to protect patient privacy.
Recruitment	For the genomic analysis, participants were retrospectively identified from the GuardantINFORM™ database based on having undergone Guardant360 liquid biopsy testing between March 2014 and June 2021. For the clinical case studies, patients were enrolled in the ongoing KN-8701 Phase I/Ib clinical trial (NCT04913285) at their respective study sites upon disease progression.
Ethics oversight	For the database analysis: Participants' consent was not necessary because deidentified research data sets generated by Guardant Health are approved by the Advarra Institutional Review Board with a waiver of consent. The GuardantINFORM database is a fully deidentified database that complies with sections 164.514(a)-(n)1ii of the U.S. HIPAA regarding the determination and documentation of statistically deidentified data. For the clinical case studies: The case studies from the KN-8701 trial were conducted in compliance with the Declaration of Helsinki and were approved by the Institutional Review Boards (IRBs) at the respective study sites (Stanford University School of Medicine and University of California San Diego).

Note that full information on the approval of the study protocol must also be provided in the manuscript.

Field-specific reporting

Please select the one below that is the best fit for your research. If you are not sure, read the appropriate sections before making your selection.

☒ Life sciences ☐ Behavioural & social sciences ☐ Ecological, evolutionary & environmental sciences

For a reference copy of the document with all sections, see nature.com/documents/nr-reporting-summary-flat.pdf

Life sciences study design

All studies must disclose on these points even when the disclosure is negative.

Sample size	Sample size for the clinical data analysis was determined by including all available BRAF-mutant NSCLC cases identified in the GuardantINFORM™ database. No statistical methods were used to predetermine sample size for in vitro and in vivo experiments. Sample sizes for mouse xenograft studies (n = 6 or n = 9 per group) were chosen based on previous experience with similar pharmacologic tumor growth inhibition models to ensure adequate statistical power for assessing treatment efficacy.
Data exclusions	For the GuardantINFORM™ database analysis, synonymous mutations and those associated with clonal hematopoiesis were excluded. Additionally, cases with multiple BRAF alterations across different functional classes were excluded from the specific class-based analysis to avoid duplication. No data were excluded from the in vitro or in vivo experimental analyses.
Replication	All in vitro experiments were performed at least twice independently with comparable outcomes. Representative data are shown in the figures. For in vivo studies, experiments were conducted once with sufficient group sizes (n = 6–9) to ensure statistical validity.
Randomization	For in vivo efficacy studies, animals were enrolled when tumors reached the target volume (175–300 mm^3) and were allocated into treatment groups to ensure equal mean tumor volumes across groups. For in vitro experiments and retrospective database analyses, randomization was not relevant to the study design.
Blinding	Blinding was not performed during data collection or analysis for in vivo experiments due to the open-label nature of the study design. For the retrospective database analysis, blinding was not applicable as the data were de-identified and analyzed based on objective genomic criteria.

Reporting for specific materials, systems and methods

We require information from authors about some types of materials, experimental systems and methods used in many studies. Here, indicate whether each material, system or method listed is relevant to your study. If you are not sure if a list item applies to your research, read the appropriate section before selecting a response.

Materials & experimental systems

n/a	Involved in the study
☐	☒ Antibodies
☐	☒ Eukaryotic cell lines
☒	☐ Palaeontology and archaeology
☐	☒ Animals and other organisms
☐	☒ Clinical data
☒	☐ Dual use research of concern
☒	☐ Plants

Methods

n/a	Involved in the study
☒	☐ ChIP-seq
☒	☐ Flow cytometry
☒	☐ MRI-based neuroimaging

Antibodies

Antibodies used	The following antibodies were used: pan-RAS (Cytoskeleton, AESA02, 1:1000), ARAF (Santa Cruz, sc-166771, 1:1000; Santa Cruz, sc-408, 1:1000), BRAF (Santa Cruz, sc-5284, 1:1000; Cell Signaling Technology, 14814, 1:1000), CRAF (BD Biosciences, BD-610152, 1:1000; Cell Signaling Technology, 9422, 1:1000), phospho-MEK (Ser217/221) (Cell Signaling Technology, 9154, 1:1000), phospho-MEK (Ser218/222) (Santa Cruz, sc-81503, 1:1000), MEK (BD Biosciences, BD-610122, 1:1000; Cell Signaling Technology, CST 9122, 1:1000), phospho-ERK (Thr202/Tyr204) (Cell Signaling Technology, 9101, 1:1000), ERK (Cell Signaling Technology, 9102, 1:1000), phospho-RSK (Thr359/Ser363) (Cell Signaling Technology, 9344, 1:1000), RSK (Cell Signaling Technology, 9355, 1:1000), phospho-AKT (Ser473) (Cell Signaling Technology, 4060, 1:1000), AKT (Cell Signaling Technology, 4691, 1:1000), phospho-PRAS40 (Thr246) (Cell Signaling Technology, 13175, 1:1000), PRAS40 (Cell Signaling Technology, 2610, 1:1000), DUSP6 (Cell Signaling Technology, 39441, 1:1000), Cyclin D1 (Cell Signaling Technology, 55506, 1:1000), EphA2 (Cell Signaling Technology, 6997, 1:1000), BIM (Cell Signaling Technology, 2933, 1:1000), EGFR (Cell Signaling Technology, 4267, 1:1000), AXL (Cell Signaling Technology, 8661, 1:1000), MET (Cell Signaling Technology, 8198, 1:1000), GAPDH (Santa Cruz Biotechnology, sc-365062, 1:1000), phospho-CHK2 (Thr68) (Cell Signaling Technology, 2661, 1:1000), phospho-H2A.X (Ser139) (Cell Signaling Technology, 2577, 1:1000), PARP (Cell Signaling Technology, 9542, 1:1000).
Validation	All antibodies were validated with RNAi mediated knockdown approaches or based on the expected changes in those proteins upon treatment with inhibitors. Most of the antibodies used in this study have been extensively used in previously published studies.

Eukaryotic cell lines

Policy information about cell lines and Sex and Gender in Research

Cell line source(s)	NSCLC cell lines harboring BRAF mutations (NCI-H2405, NCI-H1755, NCI-H1666, HCC364, NCI-H1395, NCI-2087, Cal-12T) and various RAS mutations (NCI-H358, NCI-H23, NCI-H2030, NCI-H1573, A-427, A549, NCI-H441, NCI-H1944, Calu-6) were used in this study. HCC364-VR1, a vemurafenib-resistant subclone, was previously generated in our laboratory. Cells were maintained in RPMI-1640 medium (Cytiva), except for A-427, which was cultured in DMEM (Cytiva). All media were supplemented with 10% fetal bovine serum (FBS; Avantor) and 1% penicillin-streptomycin (Cytiva), and cells were cultured at 37°C. in a humidified atmosphere containing 5% CO2. AALE, a normal epithelial cell line, was kindly provided by Eric Collison, University of Washington School of Medicine, and was maintained in SABM supplemented with SAGM SingleQuots (Lonza). HCC364 was kindly provided by David Solit, Memorial Sloan Kettering Cancer Center (MSKCC), New York. All other cell lines were obtained from the American Type Culture Collection (ATCC).
Authentication	All cell lines were authenticated by short tandem repeat (STR) profiling (November 2021).
Mycoplasma contamination	All cell lines tested negative for mycoplasma contamination.
Commonly misidentified lines (See ICLAC register)	N/A

Animals and other research organisms

Policy information about studies involving animals; ARRIVE guidelines recommended for reporting animal research, and Sex and Gender in Research

Laboratory animals	The study used female BALB/c nude mice (6–8 weeks old) and female athymic nude-Foxn1nu mice (6–8 weeks old).
Wild animals	The study did not involve wild animals.

Reporting on sex	Only female mice were used for in vivo experiments. Sex-based analysis was not performed for animal studies as only female mice were utilized.
Field-collected samples	The study did not involve samples collected from the field.
Ethics oversight	All animal experiments adhered to the procedures approved by the Institutional Animal Care and Use Committee (IACUC) at each respective institution where the studies were conducted: Pharmaron (Beijing, China), Champions Oncology (Hackensack, USA), XenoSTART (San Antonio, USA), and GenenDesign (Shanghai, China).

Note that full information on the approval of the study protocol must also be provided in the manuscript.

Clinical data

Policy information about clinical studies
All manuscripts should comply with the ICMJE guidelines for publication of clinical research and a completed CONSORT checklist must be included with all submissions.

Clinical trial registration	NCT04913285
Study protocol	https://clinicaltrials.gov/study/NCT04913285
Data collection	Patients were recruited for this study from August 2021.
Outcomes	This study aims to evaluate exarafenib (KIN-2787) in adults with BRAF/NRAS-mutated advanced or metastatic solid tumors. The primary outcomes include safety, pharmacokinetics (PK), and preliminary efficacy. The study will monitor adverse events and assess initial tumor response. The primary completion is estimated in December 2024.

Plants

Seed stocks	N/A
Novel plant genotypes	N/A
Authentication	N/A
